# Potent Inhibition of *E. coli* DXP Synthase by a *gem*-Diaryl Bisubstrate Analog

**DOI:** 10.1021/acsinfecdis.3c00734

**Published:** 2024-03-21

**Authors:** Lauren B. Coco, Eucolona M. Toci, Percival Yang-Ting Chen, Catherine L. Drennan, Caren L. Freel Meyers

**Affiliations:** † Department of Pharmacology and Molecular Sciences, The Johns Hopkins University School of Medicine, Baltimore, Maryland 21205, United States; ‡ Department of Chemistry, Massachusetts Institute of Technology, Cambridge, Massachusetts 02139, United States; § Howard Hughes Medical Institute, Department of Biology, Massachusetts Institute of Technology, Cambridge, Massachusetts 02139, United States

**Keywords:** 1-deoxy-d-xylulose 5-phosphate synthase, bacterial metabolic branch point, structure−activity relationship, infectious disease, drug discovery, cation−π interactions

## Abstract

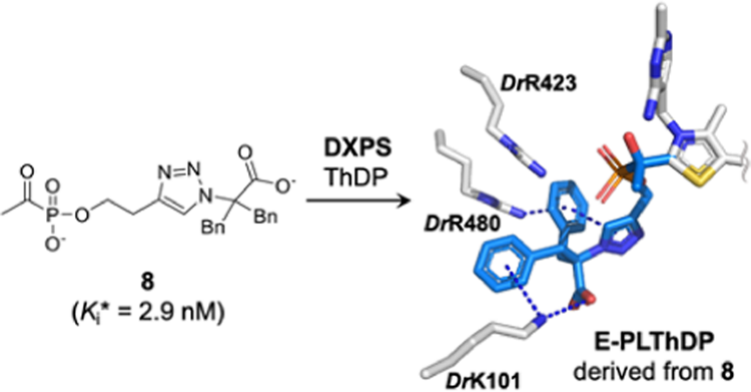

New antimicrobial strategies are needed to address pathogen resistance to currently used antibiotics. Bacterial central metabolism is a promising target space for the development of agents that selectively target bacterial pathogens. 1-Deoxy-d-xylulose 5-phosphate synthase (DXPS) converts pyruvate and d-glyceraldehyde 3-phosphate (d-GAP) to DXP, which is required for synthesis of essential vitamins and isoprenoids in bacterial pathogens. Thus, DXPS is a promising antimicrobial target. Toward this goal, our lab has demonstrated selective inhibition of *Escherichia coli* DXPS by alkyl acetylphosphonate (alkylAP)-based bisubstrate analogs that exploit the requirement for ternary complex formation in the DXPS mechanism. Here, we present the first DXPS structure with a bisubstrate analog bound in the active site. Insights gained from this cocrystal structure guided structure–activity relationship studies of the bisubstrate scaffold. A low nanomolar inhibitor (compound **8**) bearing a *gem*-dibenzyl glycine moiety conjugated to the acetylphosphonate pyruvate mimic via a triazole-based linker emerged from this study. Compound **8** was found to exhibit slow, tight-binding inhibition, with contacts to *E. coli* DXPS residues R99 and R478 demonstrated to be important for this behavior. This work has discovered the most potent DXPS inhibitor to date and highlights a new role of R99 that can be exploited in future inhibitor designs toward the development of a novel class of antimicrobial agents.

## Background

Antimicrobial resistance predates the discovery of antibiotics and is an unavoidable threat in the treatment of infectious diseases. To combat resistance, new antibacterial strategies are needed that are selective for pathogens and avoid toxicity to the host microbiome, which can negatively impact human health.^1−3^ Agents that target central metabolic processes are increasingly sought^4−15^ and have the potential to exert narrow-spectrum activities.^4,16−19^ However, this target space is relatively underexploited owing to the complexities in developing central metabolism targets and the perceived difficulties in selectively targeting bacterial metabolism over host metabolism.^4,15,16,20^ Essential to bacterial central metabolism and absent in humans, 1-deoxy-d-xylulose 5-phosphate synthase (DXPS) emerged as a promising antibacterial target with the potential to offer opportunities for developing narrow spectrum approaches.^5,21−31^

**Figure 1:**
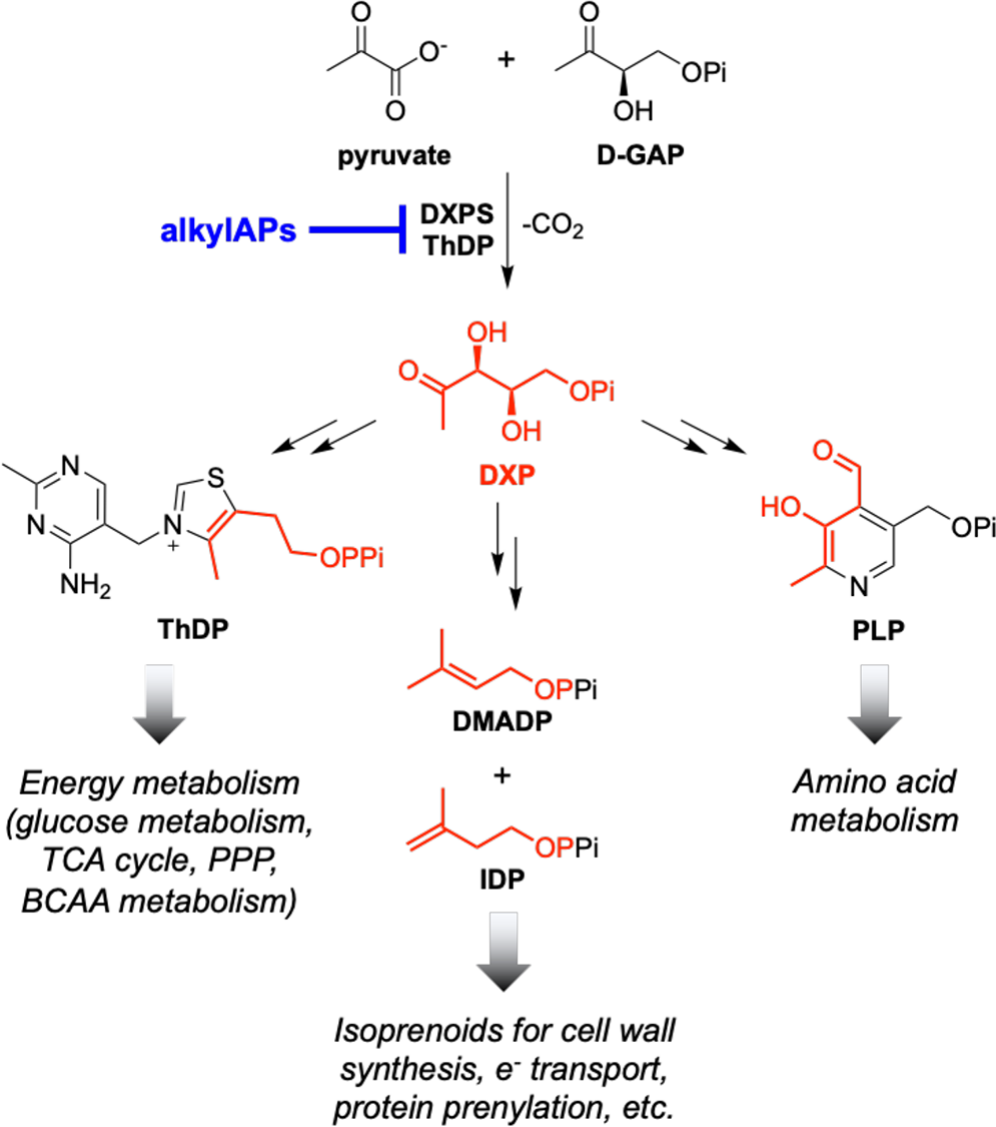
DXPS roles in bacterial central metabolism. The product DXP is used for synthesis of vitamins (ThDP and PLP) and isoprenoids. AlkylAPs inhibit DXPS catalysis, simultaneously impairing multiple metabolic pathways. Abbreviations: alkyl acetylphosphonates (alkylAPs); 1-deoxy-d-xylulose 5-phosphate (DXP) synthase (DXPS); dimethylallyl diphosphate (DMADP); isopentenyl diphosphate (IDP); methylerythritol phosphate (MEP); pyridoxal phosphate (PLP); thiamin diphosphate (ThDP); tricarboxylic acid (TCA); branched-chain amino acid (BCAA); and pentose phosphate pathway (PPP).

DXPS catalyzes the decarboxylative condensation of pyruvate and d-glyceraldehyde 3-phosphate (d-GAP) in a thiamin diphosphate (ThDP)-dependent manner to form DXP. DXP is a versatile metabolite that, depending on cellular requirements and nutrient availability, may be shuttled for use in vitamin biosynthesis (ThDP and pyridoxal phosphate) and generation of isoprenoid precursors via the methylerythritol phosphate (MEP) pathway (Figure 1). One host immune response is to starve bacterial pathogens by depleting nutrient composition at the site of infection.^13,32^ We posit that in the nutrient-limited, fluctuating host environments during infection, pathogens should be particularly sensitive to the loss of DXPS function in three essential pathways (Figure 1).

**Figure 2:**
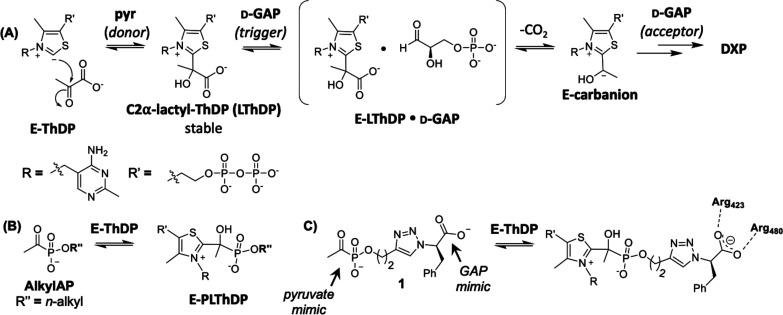
DXPS mechanism and inhibition by alkylAPs. (A) DXPS follows a ligand-gated mechanism, in which C2α-lactylThDP (LThDP) is long-lived on DXPS in the absence of d-GAP. Formation of the E-LThDP-d-GAP ternary complex induces LThDP decarboxylation. (B) Alkyl acetylphosphonates (AlkylAPs) are converted to the corresponding phosphonolactylThDP (E-PLThDP) species upon binding in the DXPS active site and reaction with ThDP. (C) Bisubstrate analog **1**. The carboxylate distal to the AP group engages the d-GAP binding pocket.

Interest in the development of DXPS as an antibacterial target has motivated studies of DXPS homologues to identify targetable features of this enzyme.^21,26,27,33−46^ In fact, DXPS has many attributes which make it a promising therapeutic target, including a large active site compared to mammalian ThDP-dependent enzymes,^36,37^ remarkable substrate promiscuity,^37,43,44,47^ and a unique mechanism in ThDP enzymology.^31,36,38,48−52^ Mechanistic studies of *Escherichia coli* DXPS elucidated a conformationally driven ligand-gated mechanism (Figure 2A), in which ternary complex formation (E-LThDP-GAP) is required for efficient decarboxylation of the first enzyme-bound intermediate in the reaction pathway, C2α-lactylThDP (E-LThDP).^31,48,49,52,53^ Unique to the DXPS mechanism, LThDP is long-lived on DXPS in a closed conformation.^31,36,38^ Binding of d-GAP causes a shift in DXPS conformation to an open form that coincides with LThDP decarboxylation.^49,52^ The resulting carbanion (E-carbanion) reacts with d-GAP as an acceptor substrate in a carboligation event to form DXP. In addition to d-GAP, DXPS responds to other inducers of LThDP decarboxylation and acceptor substrates, including O_2_, which induces LThDP decarboxylation in the absence of the natural acceptor and is subsequently reduced by the C2α-carbanion en route to peracetate.^51^

The distinctive aspects of the DXPS mechanism have guided the development of selective inhibitors of DXPS. The stable E-LThDP complex and the E-LThDP-GAP ternary complex represent enzyme states that are unique to DXPS and can be exploited for selective probe development.^24,25,27,37,54,55^ For example, alkyl acetylphosphonates (alkylAPs), including methyl acetylphosphonate (MAP), are pyruvate mimics^36,48,56−60^ that can be designed to selectively target the large DXPS active site (Figure 2B). The acetylphosphonate moiety reacts with ThDP on DXPS to form a stable, enzyme-bound phosphono-LThDP complex (E-PLThDP) that mimics the stable E-LThDP complex, similar to MAP.^36,61,62^ Enhanced inhibitory activity and selectivity of alkylAP inhibitors is achieved using bisubstrate analog scaffolds that exploit the close proximity of substrate binding sites in the DXPS active site and the requirement for ternary complex formation.^24^ The most potent analogs bear the acetylphosphonate mimic of pyruvate tethered via a triazole linker to a moiety with negative charge character, mimicking the d-GAP phosphoryl group that engages the cationic d-GAP binding pocket (Figure 2C).^24,52^ A triazole-based acetylphosphonate (TrAP) analog from this series, derived from d-phenylalanine (d-PheTrAP, **1**), displays submicromolar inhibitory activity and high selectivity for DXPS over the structurally similar ThDP-dependent enzyme pyruvate dehydrogenase (PDH).^24^ Molecular docking predicted that the PLThDP adduct derived from **1** engages the *Deinococcus radiodurans* (*Dr*) DXPS GAP binding site through interactions between the carboxylate group and two arginine residues, *Dr*R423 and R480 (Figure 2C). Reduced inhibitory activity of **1** on *Ec*R478A (analogous to *Dr*R480) offered further support that this analog behaves as a bisubstrate analog inhibitor, engaging both pyruvate and GAP binding sites.

This study aimed to gain further structural insights into the binding mode of **1** and explore modifications to this inhibitor scaffold that could impact how bisubstrate analogs engage the GAP binding pocket. Here, we report a 1.98 Å resolution *Dr*DXPS-PLThDP structure obtained from anaerobic cocrystallization of *Dr*DXPS with **1**, revealing hydrogen bonding, charge–charge, and cation−π interactions between the d-Phe triazole of **1** and *Dr*DXPS residues Y395, R423, R480, and K101, respectively. Building on structural insights, we designed and synthesized compounds modified at the α-position and bearing carboxylate, hydroxymethyl, or other aromatic substituents predicted to participate in cation–π interactions in the GAP binding pocket. Several analogs display submicromolar inhibitory activity against *Ec*DXPS; among these, we have identified a *gem*-dibenzyl TrAP analog (**8**), displaying low nanomolar potency. Using kinetics analysis and circular dichroism (CD) methods, we demonstrate inhibitor features and DXPS active site residues that are critical for potency and time dependence of inhibition by analog **8** and show that this compound is selective for DXPS by a mechanism involving PLThDP formation. The results of this study are important because they establish new features of bisubstrate analog inhibitors of DXPS that can be exploited to develop probes to investigate DXPS function in pathogens and for antibacterial design.

## Results

### Cocrystal Structure of *Dr*DXPS in the Presence of **1**

**Figure 3:**
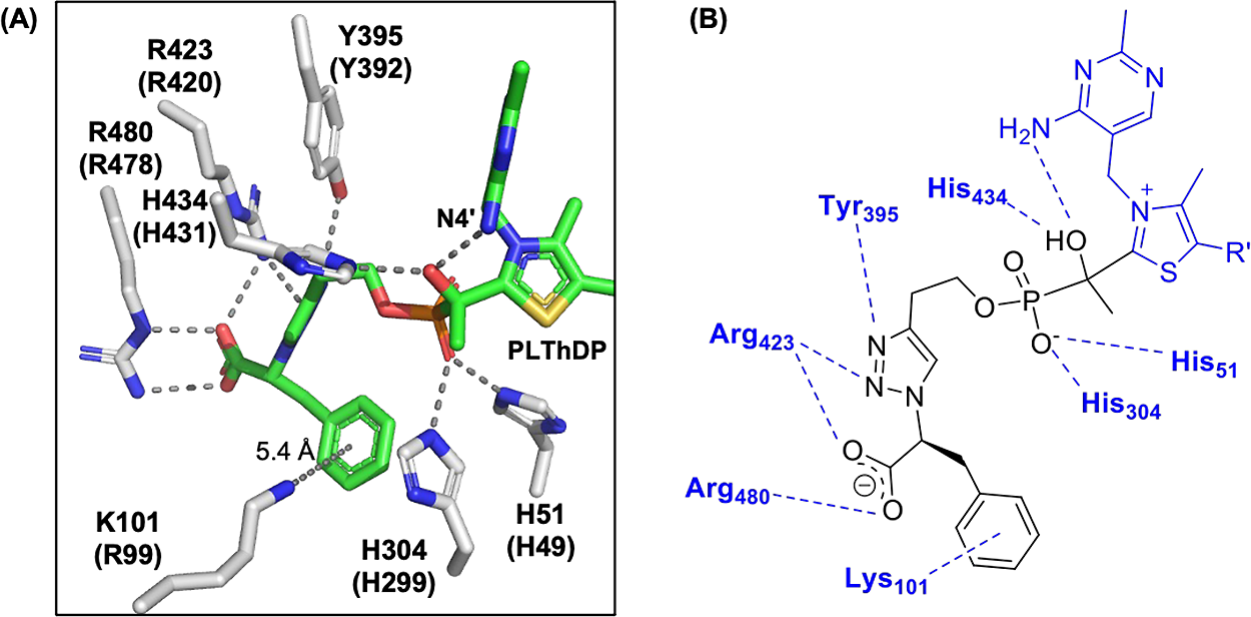
Cocrystal structure of *Dr*DXPS in the presence of **1**. (A) Structure of E-PLThDP from incubation and crystallization of *D*rDXPS in the presence of **1**, showing stabilization of the acetylphosphonate through interactions with active site His residues and interaction of the d-Phe moiety with K101 and GAP binding residues Y395, R423, and R480. DXPS residues colored in gray, PLThDP formed with **1** colored in green. Dashed lines indicate hydrogen-bonding (≤3.5 Å) and π-stacking (≤6 Å) interactions. Numbers indicate *D. radiodurans* (*E. coli*) DXPS residues. PDB code 8V9I.(B) Rendering of interactions between *Dr*DXPS and the inhibitor.

Previously, we reported the DXPS-PLThDP structure obtained from cocrystallization of *Dr*DXPS with MAP.^36^ Given that O_2_ induces LThDP decarboxylation,^51^ potentially contributing to DXPS conformational flexibility, crystallization was conducted under anaerobic conditions. Using a similar approach, *Dr*DXPS was incubated and crystallized with excess **1**, and the resulting DXPS-PLThDP structure was solved to 1.98 Å resolution in the same space group and cell dimensions as the DXPS-PLThDP structure obtained from cocrystallization of DXPS with MAP (Tables S1 and S2). The conformation of DXPS bound to **1** is similar to DXPS bound to MAP (PDB code 6OUV) with a root mean square deviation (RMSD) of 0.05 Å (of 1068 Cα in a dimer). The PLThDP adduct is evident in the active site with H51 and H304 forming favorable electrostatic interactions with the phosphonate moiety and H434 and N4′ of ThDP within hydrogen bond distance of the phosphonolactyl hydroxyl group (Figure 3). As predicted, GAP binding residues R423 and R480 form favorable electrostatic interactions with the carboxyl group of the d-Phe moiety. Y395, also implicated in d-GAP binding,^52^ and R423 form hydrogen bonds with the triazole ring (Figure 3). The benzyl group of the d-Phe moiety is in close proximity to K101, suggesting a potential cation−π interaction. A 1,2,4-butanetriol molecule from the crystallization condition is also found in the active site, in one of two subunits, and forms hydrogen bonds with the backbone carboxyl group of I301 and the phosphonate moiety of the PLThDP adduct. This and other conformational differences in the active site relative to the MAP-bound structure are shown in Figure S1.

Our recent studies of *Ec*R99 (analogous to *Dr*K101) suggest that this residue functions in an active site network that coordinates LThDP on the long-lived E-LThDP complex in its closed state;^31^ thus, this network may also be important for stabilizing PLThDP adducts. Based on the proximity of K101 to the benzyl group of **1** (Figure 3), we hypothesized that a cation−π interaction may also contribute to the potent DXPS inhibition observed for **1**. In studies presented below, we sought to test this hypothesis and explore how changing the nature of substituents engaging the GAP binding pocket influences inhibitor potency.

### Design and Synthesis of Novel Bisubstrate Analog Inhibitors

**Figure 4:**
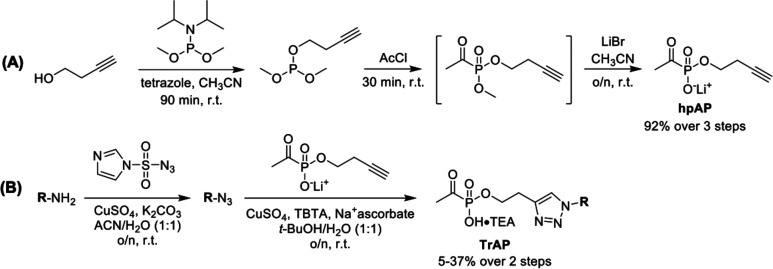
Synthesis of bisubstrate analog inhibitors. (A) Synthesis of homopropargyl acetylphosphonate (hpAP) and (B) general route for synthesis of triazole acetylphosphonate (TrAP)-based bisubstrate analogs.

Analogs were designed initially to explore the effects of changing the number, positioning, and/or electronic properties of aromatic groups and to assess the requirement for aromaticity at the position α to the carboxylate group of **1**. Inhibitors were synthesized via copper(I)-catalyzed azide–alkyne cycloaddition (CuAAC) reactions (Figure 4). The common acetylphosphonate-containing precursor, homopropargyl acetylphosphonate (hpAP), was prepared by either of two routes. Preparation of hpAP via Michaelis–Arbuzov chemistry starting from PCl_3_ and homopropargyl alcohol, as previously reported,^24^ provided hpAP in low to modest yields. In contrast, hpAP synthesis starting from dimethyl-*N*,*N*-diisopropylphosphoramidite (Figure 4A) resulted in reproducibly high yields of hpAP. Reaction of homopropargyl alcohol with dimethyl-*N*,*N*-diisopropylphosphoramidite afforded but-3-yn-1-yl dimethyl phosphite, which was immediately subjected to Michaelis–Arbuzov reaction conditions by treatment with acetyl chloride. The resulting acetylphosphonate diester was subjected to dealkylation by treatment with LiBr to give hpAP in 92% yield over three steps. This high-yielding synthesis enabled gram-scale preparation of hpAP for these studies. For CuAAC reactions, commercially available amines were converted to the corresponding azides by reaction with a sulfonyl-based diazotransfer reagent.^63^ Azides were subsequently reacted with hpAP under standard copper(I)-catalyzed azide–alkyne cycloaddition reaction conditions^24^ to afford TrAP-based bisubstrate analog inhibitors in 5–37% yield over two steps (Supporting Information).

### Inhibitor Evaluation

To determine inhibitor potency, we used the DXPS-DXP reductoisomerae (DXPS-IspC) coupled assay to measure the activity of recombinant purified *Ec*DXPS^24,25,31,44,50^ in the presence of inhibitors. Reactions were initiated by the addition of substrates following a short preincubation of the inhibitor with enzymes, and initial velocities were determined by monitoring reduced nicotinamide adenine dinucleotide phosphate (NADPH) absorbance at 340 nm. Apparent *K*_i_ values were determined by fitting initial velocity data to the Morrison equation,^64,65^ which accounts for the tight-binding nature (*K*_i_^app^/[E]_total_ < 10)^66^ of inhibitors included in this study and allows for rigorous comparison of inhibitor potencies. In the case of such inhibitors, IC_50_ values can significantly underestimate the true potency and thus were not used to compare inhibitor potencies here. Control experiments were conducted to ensure compounds presented in this study do not inhibit IspC activity in the coupled assay system (see Methods section). Inhibitor activities were rationalized based on the predictions made from molecular docking of the corresponding PLThDP adducts into the DXPS active site.

**Figure 5:**
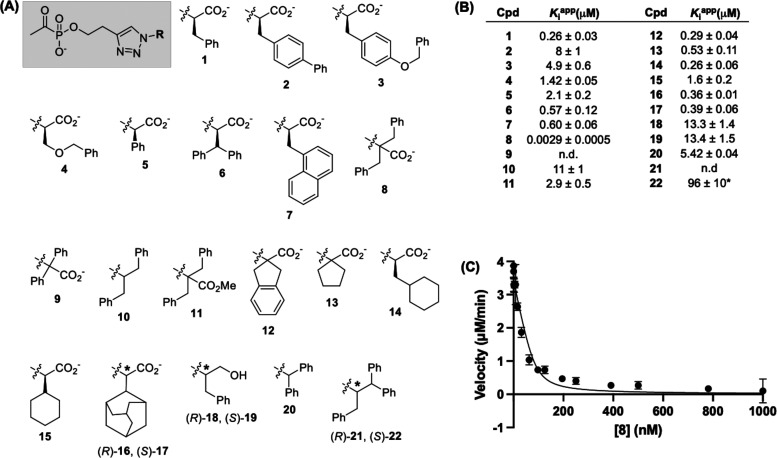
Structure–activity relationship (SAR) study of bisubstrate analog inhibitors starting from d-PheTrAP (**1**). (A) Bisubstrate analog inhibitor structures (common scaffold shaded gray). Asterisks denote chiral centers. (B) Inhibition constants determined for **1**–**8**, **10**–**20**, and **22**. Inhibitors were preincubated with *Ec*DXPS for 10 min prior to reaction initiation with substrates. (C) Inhibition data for **8** fit to the Morrison equation.

Modifications were introduced at the α–position of carboxylate **1** to investigate the effects of changing the number and/or positioning of phenyl groups (**2**–**9**, Figures 5 and S2). Incorporation of a biphenyl group (**2**, derived from *p*-phenyl-d-phenylalanine) or benzyl phenyl ether (**3**, derived from *O*-benzyl-d-tyrosine) results in reduced inhibitory activity with 30- and 19-fold increases in *K*_i_^app^ relative to **1**, respectively. Similarly, positioning the phenyl group further from the carboxylate (**4**, derived from *O*-benzyl-d-serine) increases *K*_i_^app^ by ∼5-fold. Docking of PLThDP adducts from **2**–**4** into the active site predicts a lack of engagement of the carboxyl group with the GAP binding pocket in each case, suggesting lower-affinity E-PLThDP complexes formed from **2**–**4**. PLThDP ligands generated from **3** and **4** were also predicted to adopt binding modes with the carboxy aryl group oriented away from GAP binding residues (Figure S3B–D). Interestingly, positioning phenyl closer to the carboxylate (**5**) reduces potency relative to **1** (8-fold increase in *K*_i_^app^), whereas diphenylmethyl (**6**) and naphthyl (**7**) analogs display potencies that are comparable to **1** with *K*_i_^app^ values of 0.57 ± 0.12 and 0.60 ± 0.06 μM, respectively. Docking suggests a loss of key contacts PLThDP from **5** and the GAP binding pocket, whereas adducts of **6** and **7** are predicted to have favorable interactions, with **6** suggested to make contacts with *Dr*R480 and Y395, and the **7** naphthyl group predicted to occupy a hydrophobic pocket (Figure S3E–G).

**Figure 6:**
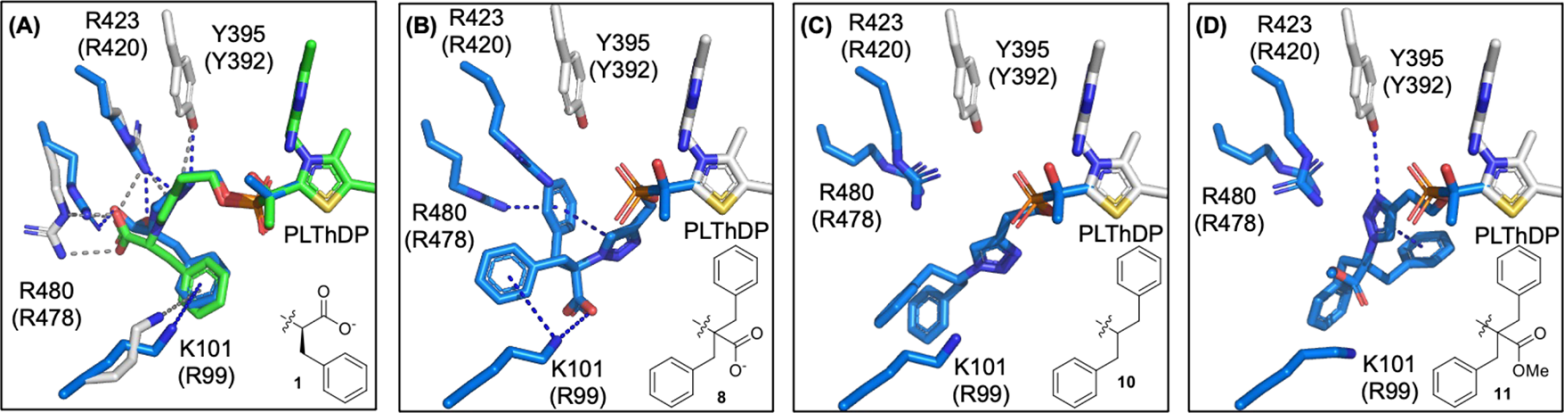
Molecular docking of PLThDP derived from **1**, **8**, **10**, and **11** into the *Dr*DXPS active site of the *Dr*DXPS-**1** crystal structure using a flexible residues approach in AutoDock Vina. (A) Docked PLThDP of **1** (blue) shows similar orientation and residue contacts (docked: blue dashed lines; crystal: gray dashed lines) as the crystal pose (residues colored gray, PLThDP colored green). (B) Docked PLThDP of **8** predicts contacts to key active site arginine residues (*Ec*R420, R478, and R99) through hydrogen bonding (≤3.5 Å) and π-stacking (≤6 Å) interactions (blue dashed lines). (C) PLThDP adduct derived from descarboxy analog **10** and (D) methyl ester **11** are not predicted to interact with active site Arg or Lys. *Dr*K101 (*Ec*R99) is predicted to be >3.5 Å from phenyl groups of **10** and **11**. Numbers indicate *D. radiodurans* (*E. coli*) DXPS residues. Blue coloring indicates structures allowed flexibility in the docking simulation (inhibitor and residues DrR423, R480, and K101). Structures colored light gray were not allowed flexibility in the docking simulation.

Compounds **8** and **9** were designed to assess the effects of an additional phenyl-containing substituent α to the carboxylate with the potential to gain additional favorable cation−π interactions. *gem*-Diphenyl analog **9** could not be prepared, and the descarboxy product was the major product isolated. In contrast, *gem*-dibenzyl compound **8** was isolable and found to display dramatically increased inhibitory activity compared to **1**, with a *K*_i_^app^ of 2.9 ± 0.5 nM (Figure 5B,C). Toward elucidating structural features contributing to the high potency of **8**, we prepared compounds **10**–**13**. Descarboxy analog **10** (*K*_i_^app^ = 11 ± 1 μM) and methyl ester **11** (*K*_i_^app^ = 2.9 ± 0.5 μM) display significantly reduced inhibitory activity relative to **8**, with ∼3800- and 1000-fold increases in *K*_i_^app^, respectively; this indicates that the negatively charged carboxylate group is critical for the nanomolar potency of **8**. Molecular docking analysis of PLThDP adducts derived from **8**, **10**, and **11** (Figure 6) suggests that **8** is the only one of these analogs predicted to maintain polar contacts with the d-GAP binding pocket plus cation−π interactions with *Dr*K101 and R478. In addition, a favorable π–π interaction is predicted between the triazole and one benzyl group of the inhibitor; this model is consistent with a high-affinity E-PLThDP complex formed from **8**. Spiro analogs **12** (*K*_i_^app^ = 0.29 ± 0.04 μM) and **13** (*K*_i_^app^ = 0.53 ± 0.11 μM) also show significantly reduced inhibitory activity compared to **8**, supporting the importance of the flexible *gem*-dibenzyl moiety predicted by molecular docking; however, they are comparable in potency to **1**.

As noted, analysis of the E-PLThDP structure derived from **1** predicted a favorable cation−π interaction between the benzyl group of **1** and *Dr*K101 (*Ec*R99), suggesting the importance of aromaticity at this position. Yet, desphenyl spiro analog **13** has comparable potency to **1**. Compounds **14**–**17** also lack aromaticity α to the carboxyl group and introduce increased steric bulk at this position (Figure 5). Interestingly, cyclohexyl analog **14** was found to have comparable inhibitory activity to **1**, providing additional evidence that aromaticity is not absolutely required for submicromolar potency. Molecular docking of PLThDP from **14** (Figure S3J) predicts an orientation that maintains several contacts with the GAP binding pocket, positioning the triazole group in close proximity to *Dr*R423 and Y395 (*Ec*R420 and Y392) and the carboxylate group within the hydrogen bond distance of *Dr*R480 (*Ec*R478). Docking of PLThDP ligands from spiro analogs **12** and **13** suggests that they may adopt similar orientations in the DXPS active site (Figure S3H,I). However, contacts to the GAP binding pocket are not predicted in either case, indicating that this docking approach may not accurately predict *E. coli* DXPS homologue contacts to **12** and **13** that underlie the observed submicromolar inhibitory activities. Similar to its α-phenyl counterpart (**5**), compound **15** shows micromolar inhibitory activity, with a 6-fold increase in *K*_i_^app^ relative to **1**. However, α-adamantyl analogs (*R*)-**16** (*K*_i_^app^ = 0.36 ± 0.01 μM) and (*S*)**-17 (***K*_i_^app^ = 0.39 ± 0.06 μM) display inhibitory activities that are comparable to **1**. Perhaps, appropriately placed steric bulk in the large DXPS active site can orient the inhibitor for favorable interactions to triazole and carboxylate groups. In line with this, docking analysis predicts that the PLThDP ligand from **15** lacks contacts to the GAP binding pocket, whereas interactions are predicted between *Dr*R423 and the triazole of **16** and **17**, as well as contacts between the carboxyl group and *Dr*K101 in both cases (Figure S3K–M).

Analogs **18**–**22** examine the replacement of the carboxyl group of **1** with hydroxymethyl (**18** and **19**), and in a preliminary step to explore modifications with the potential to introduce additional cation-π interactions, we prepared **20**–**22**. As expected, analogs **18** and **19** display significantly reduced potency relative to **1**, again confirming the importance of the negatively charged carboxylate of **1** (Figures 5 and S4). Further, **18** and **19** are equipotent, in contrast to **1** and its l-stereoisomer, whose *K*_i_^app^ values differ by 25-fold.^24^ This result suggests the carboxylate group is also required for favorable orientation of the phenyl group of **1** toward *Dr*K101 (*Ec*R99), supported by docking analysis (Figure S3N–P). Similar to **10**, diphenyl analog **20** displays reduced potency relative to **1** with ∼21-fold decrease in *K*_i_^app^. Replacement of the carboxyl group of **1** with a diphenylmethyl group ((*R*)-**21** or (*S*)-**22**) results in dramatic loss of inhibitory activity (Figures 5 and S4). Minimal inhibition was observed by **21** up to a concentration of 50 μM, and a 370-fold reduction in *K*_i_^app^ relative to **1** was observed for **22**. In addition, **22** displays a mixed inhibition mode, with α > 1 (Figure S4), supported by model discrimination analysis using Akaike’s information criterion.

### Selectivity of **8** for DXPS

The most potent inhibitor in this study, dibenzyl analog **8**, was investigated further to ascertain its selectivity for DXPS. As noted, DXPS has a large active site and follows a mechanism requiring ternary complex formation, distinct from other ThDP-dependent pyruvate decarboxylases. We hypothesized that the sterically demanding scaffold of **8**, predicted to engage both substrate binding sites, should display selectivity for DXPS. In a preliminary assessment, we tested **8** as an inhibitor of porcine pyruvate dehydrogenase (PDH) E1 subunit. As expected, **8** (*K*_i_^app^ = 2.9 nM) has negligible inhibitory activity against PDH, displaying 21% inhibition at a concentration of 1 mM (Figure S5). Under comparable conditions (2 × *K*_m_ pyruvate), less than 15.6 nM of **8** is required for 21% inhibition of DXPS; thus, the estimated selectivity ratio is >64,000-fold for DXPS over porcine PDH. For comparison, the selectivity ratio for **1** was estimated to be >15,000-fold for DXPS over porcine PDH.

### Time-Dependent Inhibition by **8**

**Figure 7:**
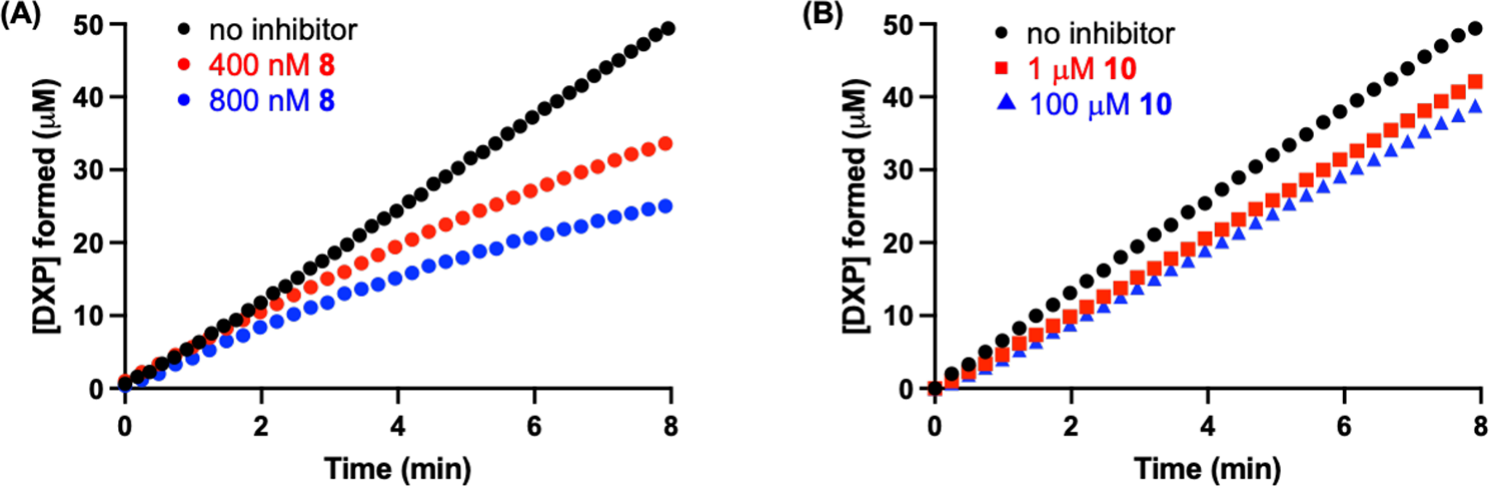
Time-dependent inhibition. (A) Representative reaction progress curves for DXP formation in the presence or absence of **8** (at 400 or 800 nM). (B) Representative reaction progress curves for DXP formation in the presence or absence of **10** (at 1 or 100 μM). Experiments were performed in triplicate (Figure S6).

We previously reported time-dependent inhibition by bisubstrate analog **1**, suggesting an induced fit mechanism,^24^ and this is consistent with our observations that ligand-dependent conformational changes of DXPS occur.^36,38^ To determine if **8** is also a slow-onset inhibitor of DXPS, reaction progress was monitored upon simultaneous addition of substrates (pyruvate and d-GAP) in the presence or absence of inhibitor. In the absence of inhibitor, the reaction progress curve is linear. In the presence of **8**, DXP formation is reduced relative to the no inhibitor control, and the progress curve is nonlinear, indicating time dependence of inhibition (Figure 7A). We have reasoned that the carboxyl group of **1** is required for time-dependent behavior, given that simple alkylAPs lacking carboxyl do not display this behavior. To assess the importance of the carboxyl group of **8**, we evaluated descarboxy analog **10** in the same manner. DXP formation in the presence of **10** is linear up to 100 μM **10** (Figures 7B and S6), suggesting that the carboxylate group is necessary for time-dependent inhibition.

### Mechanism of Inhibition by **8** Involves the Formation of a High-Affinity PLThDP Adduct

**Figure 8:**
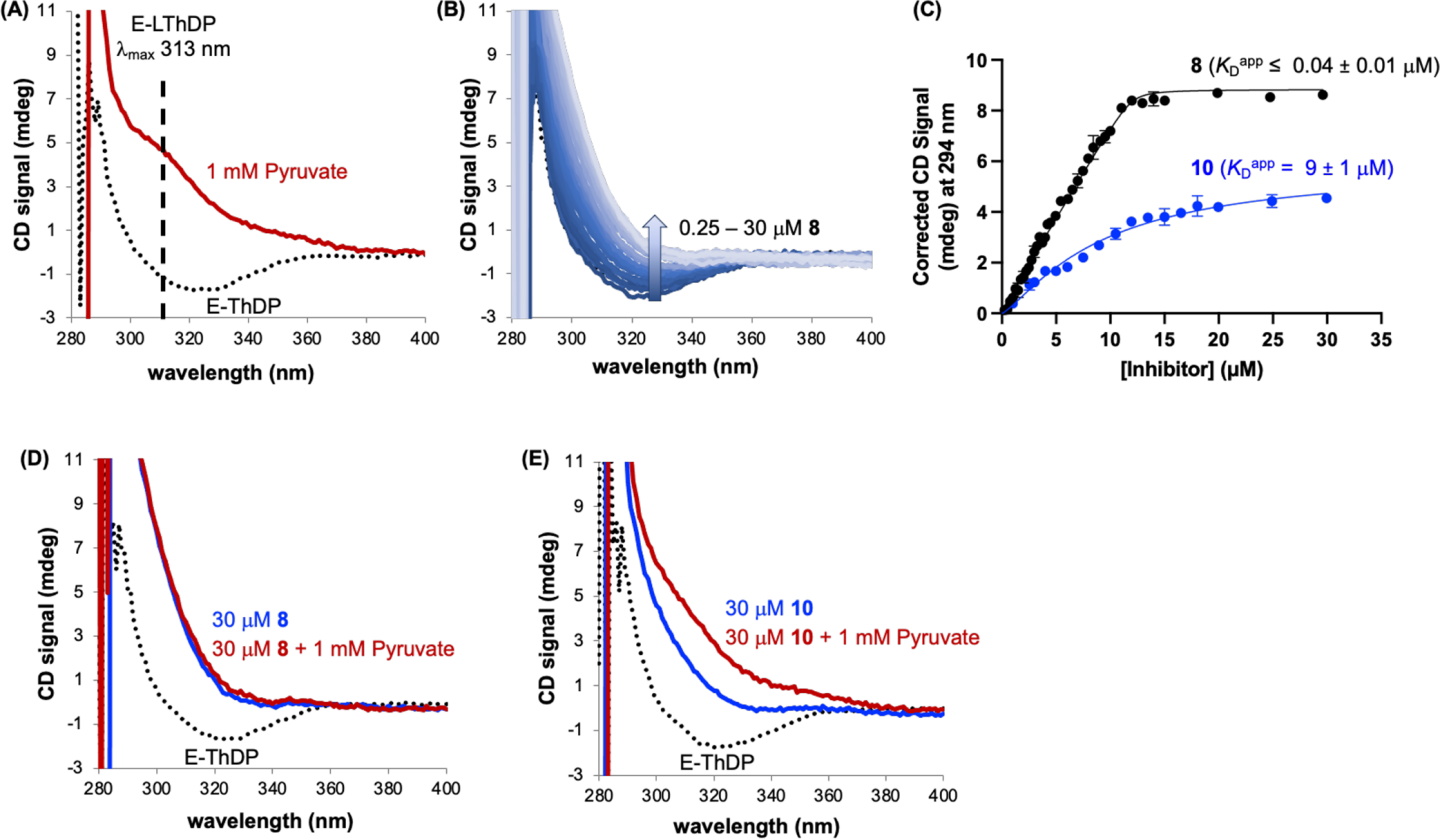
PLThDP formation from **8** or **10** on wildtype (WT) *Ec*DXPS and persistence in the presence of saturating pyruvate. (A) Addition of pyruvate to E-ThDP gives rise to a positive CD signal at 313 nm corresponding to E-LThDP. ThDP-bound DXPS, dotted black line trace; LThDP-bound DXPS, red line. (B) Addition of **8** to DXPS showing emergence of a broad positive CD signal indicative of PLThDP formation. (C) Determination of upper limit of *K*_D_^app^ for **8** and *K*_D_^app^ for **10**. Error bars represent standard error, *n* = 3. (D) Representative CD traces showing PLThDP formed on WT *Ec*DXPS in the presence of **8** (blue line), followed by addition of 1 mM pyruvate (red line). (E) Representative CD traces showing PLThDP formed on WT *Ec*DXPS in the presence of **10** (blue line), followed by addition of 1 mM pyruvate (red line).

The X-ray structure obtained from cocrystallization of *Dr*DXPS with **1** (Figure 2) indicates the formation of the PLThDP adduct by reaction of the acetylphosphonate mimic of pyruvate with the C2 carbanion of ThDP. We reasoned that **8** should also engage the pyruvate binding site and react with ThDP to form a PLThDP adduct. Circular dichroism (CD) is a powerful tool that can be used to detect LThDP and LThDP-like adducts on thiamin-dependent enzymes,^56,67−75^ including DXPS.^31,36,43,44,49−52,76^ DXPS bound to ThDP (E-ThDP) is characterized by a CD λ_min_ at 320 nm, indicative of the 4′-aminopyrimidine (AP) tautomer of ThDP (Figure 8A). Upon addition of pyruvate to E-ThDP, a new positive signal emerges at 313 nm, indicating the formation of LThDP in its 1′,4′-iminopyrimidine (IP) form. Similarly, upon addition of AP-based inhibitors to E-ThDP, the E-PLThDP adduct is formed, characterized by an increasing, broad CD signal.^69,77,78^ Determination of apparent *K*_D_ of PLThDP by plotting the CD signal corresponding to PLThDP versus [MAP] has been used as a proxy for PLThDP affinity.^77,78^ To confirm that **8** reacts with ThDP to form PLThDP in the DXPS active site, the change in CD signal upon addition of **8** (0.25–30 μM) to *Ec*DXPS (30 μM) was measured. As expected, titration of **8** resulted in an increasing, broad signal characteristic of PLThDP formation (Figure 8B). Plotting the CD signal at 294 nm versus [**8**] affords a curve that approaches the limiting asymptotes defined by infinitely tight binding (Figure 8C),^79,80^ which is expected for this nanomolar inhibitor under the conditions of high [DXPS] required for this method. Since in this case [DXPS] ≫ *K*_D_^app^, an accurate determination of *K*_D_^app^ is not possible, and data were fit to a quadratic equation to give an estimated upper limit for the value of *K*_D_^app^ (≤0.04 ± 0.01 μM). Addition of **10** to DXPS also results in an increased, broad CD signal (Figure S7A), indicating PLThDP formation. As expected, a plot of the CD signal at 294 nm versus [**10**] (Figure 8C) indicates weaker affinity of the PLThDP adduct generated from this analog (*K*_D_^app^ = 9 ± 1 μM), consistent with its reduced inhibitory activity relative to **8**.

To provide additional evidence in support of a high-affinity E-PLThDP complex generated from **8** relative to the lower-affinity E-PLThDP complex derived from descarboxy analog **10**, we investigated changes in the PLThDP CD signal that may occur upon addition of saturating pyruvate. We reasoned that if PLThDP formation is readily reversible, the addition of pyruvate should shift equilibria in favor of LThDP formation, giving rise to a more LThDP-like CD signal. When 1 mM pyruvate was added to E-PLThDP, generated by the addition of **8** (30 μM) to *Ec*DXPS (30 μM), there was a negligible shift in the CD signal (Figure 8D), consistent with the tight-binding behavior of **8**. In contrast, the addition of pyruvate to E-PLThDP generated from **10** causes a CD signal shift and an increase in amplitude, giving rise to a CD max of 313 nm, consistent with LThDP formation (Figure 8E). Together, these results offer further support for the tight-binding nature of **8** and the role of the carboxyl group in orienting **8** to engage the GAP binding pocket.

### *Ec*DXPS R99 and R478 Are Critical for Potency and Slow, Tight-Binding Behavior of **8**

**Figure 9:**
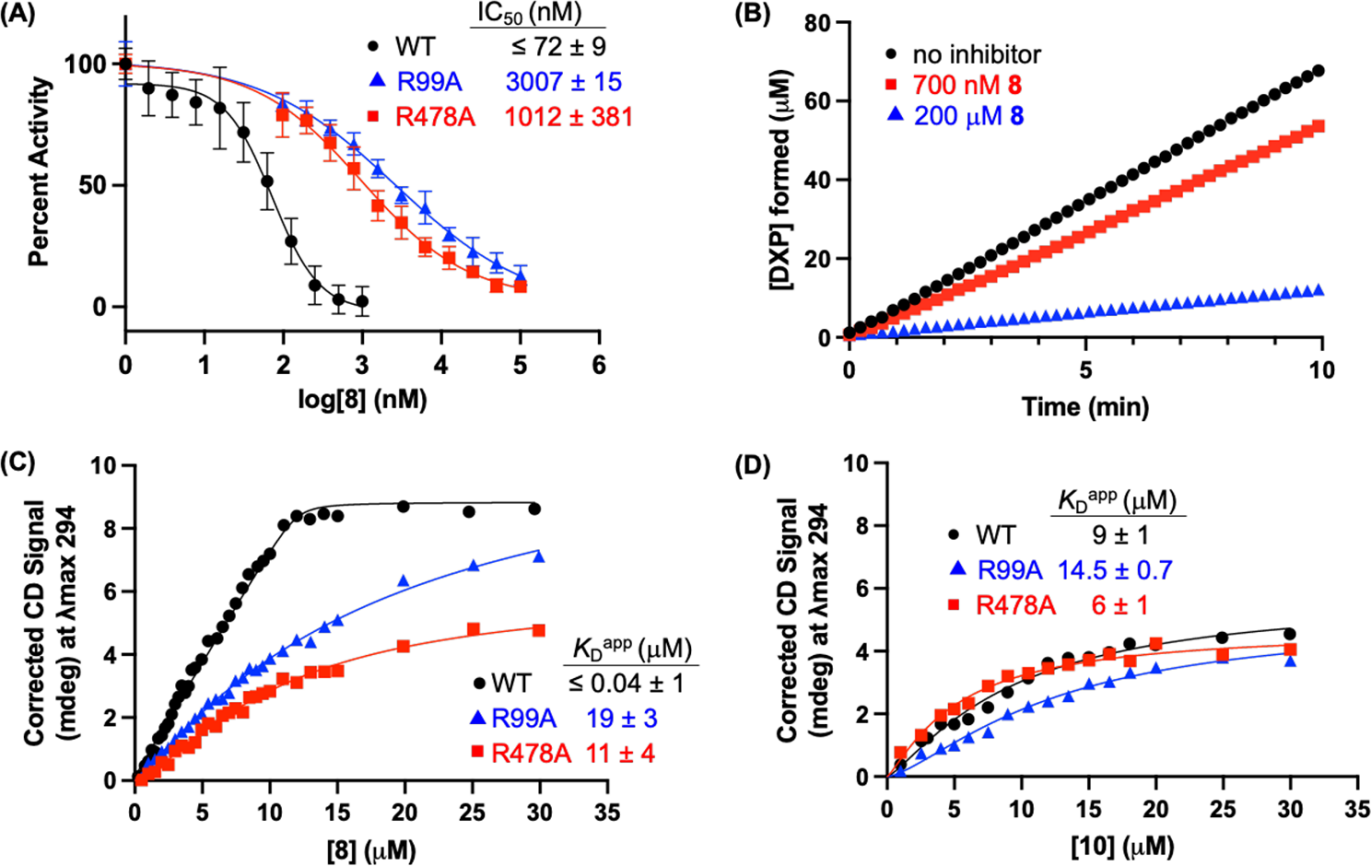
Substitution of *Ec*R99 or R478 impairs potency and binding of **8**. (A) IC_50_ determinations indicating reduced inhibitory activity of **8** against *Ec*DXPS variants relative to wildtype enzyme. Error bars represent standard error; *n* = 3. (B) Representative progress curves for DXP formation, showing that time-dependent inhibition is not observed for **8** against *Ec*R99A. (C) Representative titrations of **8** onto WT *Ec*DXPS, R99A, and R478A DXPS, showing lower affinity of PLThDP on R99A and R478A relative to wildtype. (D) Representative titrations of **10** onto WT *Ec*DXPS, R99A, and R478A DXPS, showing comparable affinity of PLThDP.

Molecular docking analysis predicted interactions between the α-*gem*-dibenzyl carboxylate moiety of **8** and *Ec*DXPS residues R99 and R478 (Figure 6). Thus, we investigated *Ec*R99A and *Ec*R478A DXPS variants (Table S3) to experimentally assess the effects of these substitutions on inhibitory activity as well as PLThDP formation and persistence. As expected, a dramatically reduced potency of **8** was observed on *Ec*R99A and R478A relative to WT *Ec*DXPS (Figure 9A). In addition, loss of time-dependent behavior was observed for **8** on R99A, evident from the linear progress curves for DXP formation on *Ec*R99A in the presence of **8** (Figures 9B and S8). This finding suggests that the ability of **8** to engage the GAP binding pocket is requisite for its time-dependent behavior and is consistent with the observed lack of time dependence observed for descarboxy analog **10** (Figure 7B), which is not predicted to interact with R99 or R478 (Figure 6C). The *Ec*R478A DXPS variant displays an exceedingly high *K*_m_^D-GAP^ (1400 ± 200 μM)^44^ and is therefore not evaluated to ascertain the role of R478 in time dependence of **8** due to the unreasonably high concentration of d-GAP (10 × *K*_m_) required for this experiment. CD analysis showed that both R99A and R478A variants catalyze PLThDP formation from either **8** or **10** as expected, evidenced by buildup of a broad positive CD signal characteristic of PLThDP adducts (Figure S7). However, in agreement with the observed loss of inhibitory activity of **8** on R99A and R478A, the apparent affinity of PLThDP generated from **8** was also significantly reduced on both variants relative to WT DXPS (Figure 9C). Conversely, the apparent affinity of PLThDP generated from **10** was comparable to WT and variants (Figure 9D), as predicted. Finally, a shift in the CD profile toward the characteristic LThDP signal was observed upon addition of pyruvate to R99A-PLThDP or R478A-PLThDP complexes generated from **8** (Figure S9). Taken together, these results strongly support a role for *Ec*R99 and *Ec*R478 in the potency and slow, tight-binding behavior of **8**. R99 was also found to contribute significantly to the affinity of PLThDP derived from **1** (Figure S7C), predicted from the crystal structure (Figure 3) and supporting a general role of R99 in stabilizing bisubstrate adducts on DXPS.

### DXPS Quaternary Structure in the Presence of **8**

**Figure 10:**
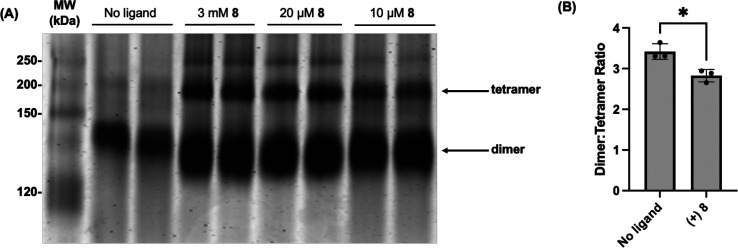
*Ec*DXPS tetramer formation in the presence of **8**. (A) Native PAGE (7.5%) showing the oligomeric state of *Ec*DXPS (20 μM) in the presence or absence of **8** at varied concentrations; *n* = 2; MW = molecular weight protein ladder (kDa). (B) Difference in the dimer/tetramer ratio of *Ec*DXPS in the presence or absence of **8** by analytical gel filtration. Error represents the standard error, *n* = 3; *p < 0.05.

DXPS is active as a homodimer. A recent study suggests that DXPS activity is regulated via ligand-induced changes to the oligomeric state.^81^ To determine the effect of **8** on quaternary structure, we conducted native polyacrylamide gel electrophoresis (PAGE) and analytical gel filtration (aGF) analyses. In the absence of the ligand, *Ec*DXPS exists mainly in the active dimer state with a small amount of tetramer observed, apparent in the native polyacrylamide gel and aGF trace (Figures 10A and S11). Incubation of *Ec*DXPS with **8** appears to induce the formation of higher-order enzyme oligomers, notably a DXPS tetramer. Similarly, aGF analysis following incubation of *Ec*DXPS with **8** shows a small but statistically significant decrease in the dimer/tetramer ratio, even in the absence of **8** in the elution buffer (Figure 10B).

## Discussion

This study focused on the bacterial central metabolic enzyme DXPS, a promising antibacterial target. Our previous work discovered the DXPS inhibitor d-PheTrAP (**1**), which is a submicromolar, time-dependent bisubstrate analog inhibitor of DXPS bearing an acetylphosphonate mimic of the donor substrate pyruvate linked to a carboxy mimic of the acceptor substrate d-GAP.^24^ Carboxyl and benzyl substituents were both found to contribute to the potency of **1**, with the carboxyl moiety making key contacts to arginine residues (*Ec*R420 and R478) in the GAP binding pocket.^24^ In the current study, we sought to gain additional structural insights into binding of **1** to DXPS toward understanding how the d-phenylalanine moiety contributes to the potency of **1** and to conduct SAR studies, exploring other substituents at the α-position.

A 1.98 Å resolution structure obtained from anaerobic cocrystallization of **1** with *Dr*DXPS offered additional insights into the molecular basis for bisubstrate analog inhibitory activity. Phosphonolactyl formation on DXPS is supported by this structure, with stabilization of the phosphonolactyl group through interactions with conserved active site His residues known to stabilize LThDP. Interactions between the d-Phe triazole moiety and GAP binding residues were also observed, as well as a potential cation−π interaction between the benzyl group of **1** and *Dr*K101 (*Ec*R99). Indeed, SAR studies supported by molecular docking analysis indicated that, generally, analogs predicted to engage residue *Ec*R99, and/or residues in the conserved GAP binding pocket (*Ec*R420, R478 and Y392), maintain submicromolar inhibitory activity against DXPS. Analog **8** emerged as the most potent analog in this series, displaying slow, tight-binding activity with a low nanomolar *K*_i_*. Bearing a *gem*-dibenzyl glycine moiety, compound **8** was predicted to adopt a binding mode allowing favorable π–π and cation−π interactions and to engage *Ec*R99 as well as GAP binding residues *Ec*R420 and R478. We confirmed that **8** makes key contacts to R478 and R99, demonstrating significantly reduced affinity of the PLThDP adduct of **8** on R478A and R99A variants and showing that R99 is required for potent, time-dependent inhibition.

The results bolster support for the carboxyl moiety as a key driver of bisubstrate analog potency in this series, with stabilizing polar contacts to Arg residues that also serve to orient the α-substituent(s) to allow multiple favorable interactions on *Ec*DXPS. The study also reveals a new targetable feature of DXPS to exploit for inhibitor design. *Ec*R99 (*Dr*K101), predicted to engage carboxyl and benzyl groups of **8** and shown to be critical for its slow, tight-binding behavior, also contributes to the submicromolar activity of **1**. Notably, an arginine residue at position *Ec*R99 is heavily conserved across DXPS homologues, with the exception of *Dr*DXPS, which has a functionally similar lysine residue in this position.^31,82^ These data suggest that targeting *Ec*R99 could be a useful strategy in the development of DXPS inhibitors. Other work from our lab suggests that *Ec*R99 functions in an active site network with a role to increase the barrier to LThDP decarboxylation in the absence of the acceptor substrate.^31^ Thus, it is conceivable that R99 plays a dual role in the E-PLThDP complex; in addition to engaging in favorable polar and/or cation–π interactions with aryl and/or carboxyl substituents of **1** and **8**, perhaps R99 also functions in a network that stabilizes the phosphonolactyl adduct formed upon binding of these bisubstrate analogs. Consistent with this idea, R99 remains within hydrogen bond distance of its neighboring residues of this active site network in the E-PLThDP structure derived from **1** and in the docked model of E-PLThDP derived from **8**.

Interestingly, analog **8** appears to shift the DXPS oligomer equilibrium, inducing higher-order oligomeric states. Di *et al*. have recently reported ligand-induced monomerization of DXPS by downstream MEP pathway intermediates,^81^ suggesting DXPS regulation by reversible reduction of DXPS dimer activity. Although the activity of the higher-order tetramer is unknown, it is conceivable that shifting the oligomer equilibrium could contribute to the inhibitory activity of this enzyme class. *Dr*DXPS is monodisperse in the dimer form,^36^ making it more amenable to crystallization than *Ec*DXPS, which has proven difficult to crystallize as the full-length protein in the presence or absence of ligands thus far. The inhibitor-induced changes to *Ec*DXPS quaternary structure observed in this work foretell similar challenges for cocrystallization of *Ec*DXPS with these analogs.

This work represents a significant advance in DXPS inhibitor development, delivering the most potent DXPS inhibitor to date and offering key insights regarding DXPS determinants of inhibitor potency that can be exploited toward the development of a novel class of antimicrobial agents.

## Methods

### General

Reagents and solvents used for chemical synthesis were purchased from commercial sources and used without further purification. Chemicals were purchased from Millipore Sigma (Sigma-Aldrich), unless otherwise noted. 4-(2-Hydroxyethyl)-1-piperazineethanesulfonic acid (HEPES) and lysogeny broth (LB) were purchased from Fisher Scientific. Inhibitors were purified by flash chromatography using a Biotage Isolera One system. Compound identify was confirmed by ^1^H and ^31^P NMR and high-resolution mass spectrometry (HRMS). NMR spectra were recorded using a JEOL or Bruker 500 MHz NMR spectrometer. ^1^H NMR chemical shifts are reported in δ (ppm) relative to chloroform-*d* at 7.27 ppm, methanol-*d*_4_ at 3.31 ppm, dimethyl sulfoxide (DMSO)-*d*_6_ at 2.50 ppm, or deuterium oxide at 4.79 ppm. ^31^P NMR chemical shifts are reported relative to an external standard of triphenylphosphine oxide (TPPO) in benzene-*d*_6_ set to 0.0 ppm. Couplings are described as singlet (s), doublet (d), triplet (t), quartet (q), or multiplet (m). High-resolution mass spectrometry was acquired on each newly reported compound at the Mass Spectrometry Laboratory, School of Chemical Sciences, University of Illinois Urbana–Champaign, IL, using a Waters Synapt G2SI with the Acquity I-class system, with flow injection using H_2_O/acetonitrile (1:1) as carrier phases. Compound purity was assessed by high-field NMR and reversed-phase analytical high-performance liquid chromatography (HPLC) (Agilent LC 1260 Infinity II system, equipped with a diode array detector).

All experiments on DXPS, except the crystallographic study, were performed using the *E. coli* protein. *E. coli* DXPS and *E. coli* IspC were overexpressed and purified as previously reported.^48,76^ An AKTA-GO fast protein liquid chromatography (FPLC) system was used for protein purification. Tecan Infinite M Nano U/V visible plate readers and a BioTek Epoch 2 microplate reader were used at 25 °C for aerobic spectrophotometric analyses. CD experiments were performed on an Applied Photophysics Chirascan V100 CD spectrometer (Surrey, U.K.). Macromolecule structural figures were created using PyMOL version 2.5.4.

### Cocrystallization of *Dr*DXPS with **1**

*Dr*DXPS was overexpressed in *E. coli* BL21 (DE3) cells harboring dxs-pET28b(+)^33^ and purified as described previously.^36^*Dr*DXPS with d-PheTrAP (**1**) was crystallized using the sitting drop crystallization method in an MBRAUN anaerobic chamber with a N_2_/H_2_ environment at room temperature. *Dr*DXPS (1.0 μL of 25.0 mg/mL) in buffer containing 10 mM HEPES, pH 8.0, 5% (v/v) glycerol, 200 mM NaCl, and 0.5 mM tris(2-carboxyethyl)phosphine (TCEP), and 1.2 equiv of **1** was mixed with 1.0 μL well solution to make a 2 μL sitting drop in a sealed well with 500 μL well solution. The well solution contained 0.056 M BES (Acros), 0.044 M triethanolamine, pH 7.5, 15% (w/v) poly(ethylene glycol) (PEG) 3000 (Rigaku Chemicals), 20% (v/v) 1,2,4-butanetriol (Alfa Aesar), 1% (w/v) nondetergent sulfobetaines (NDSB)-256 (Hampton), 25 mM l-arginine, 25 mM l-threonine, 25 mM l-histidine, and 25 mM *trans*-4-hydroxy-l-proline. Transparent plate crystals grew in 12–24 h. The crystal used to determine the structure was looped from the drop directly and flash-cooled in liquid nitrogen.

### Structure Determination and Refinement

Data were collected at the Advanced Photon Source on Northeastern Collaborative Access Team beamline 24-ID-C on a Pilatus 6MF detector. All data were indexed and scaled in HKL2000^83^ with a CC1/2 of ∼0.8 used as the indicator of where to trim the high-resolution data (Table S1). The structure of *Dr*DXPS cocrystallized with **1** has two protomers in the asymmetric unit and was determined to 1.98 Å resolution by rigid-body refinement from the 1.94 Å resolution structure of *Dr*DXPS with MAP bound (PDB ID: 6OUV).^36^ The initial model for the rigid-body refinement contains all atoms of the structure of *Dr*DXPS with MAP bound except for non-magnesium-bound water molecules, which were manually removed. Rwork/Rfree after the initial refinement was 23.8/24.9%. Refinement of atomic coordinates and B-factors was performed iteratively in Phenix Refine^84^ with model building and manual adjustments in Coot.^85^ Programs used were compiled by SBGrid.^86^ Final cycles of refinements included TLS parametrizations with one TLS group for the protein monomer. Water molecules were added manually using Fo–Fc electron density contoured to 3.0σ as the criteria. Two-fold noncrystallographic symmetry (NCS) restraints were used throughout refinement. Restraints for d-PheTrAP-TPP (**1**-TPP) were from Phenix.eLBOW.^87^ Composite-omit electron density maps calculated by phenix.composite_omit_map^84^ were used to verify the model. The residues and cofactors modeled in the final structure are given in Table S2 with refinement statistics in Table S1.

### Determination of Apparent *K*_i_

All compounds in this study were evaluated as DXPS inhibitors using the DXPS-IspC coupled assay as previously reported.^24^ For determination of apparent *K*_i_ for **1**–**8**, **10**–**17**, and **20**, *Ec*DXPS (100 nM) and the inhibitor (98 nM to 100 μM for compounds **1**–**7**, **10**–**17**, and **20**; 1 nM to 100 μM was used for compound **8**) were preincubated at 25 °C for 10 min in enzyme buffer (100 mM HEPES pH 8, 2 mM MgCl_2_, 5 mM NaCl, 1 mM ThDP, 200 μM NADPH and 2 μM IspC). The 10 min preincubation was deemed sufficient for determination of *K*_i_* for the most potent compound **8**; *K*_i_* values determined following 10 or 20 min preincubation were comparable (Figure S2C). The DXPS reaction was initiated by the addition of substrates (50 μM pyruvate and 500 μM d-GAP). The initial rate of depletion of NADPH was monitored at 340 nm and was used to calculate the initial rate of DXP formation in the presence or absence of inhibitor. Initial rates of DXP formation were plotted as a function of [inhibitor], and *K*_i_^app^ was determined by fitting the data to the Morrison equation (eq 1)^64,65^ in GraphPad Prism. This approach was deemed appropriate as the concentration of free inhibitor cannot be assumed to remain constant for most of these analogs in which *K*_i_^app^ values approach the concentration of DXPS.^88,89^ Error is reported as standard deviation from three replicates. Active enzyme concentration ([E]_T_) was determined following Copeland’s method using **8** (Figure S2B).^88^ For the most potent analog (**8**), the average *K*_i_^app^ was determined from three replicates using two separate preparations of DXPS.
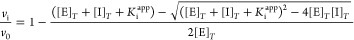
1

For determination of apparent *K*_i_ for **18**, **19**, **21**, and **22**, *Ec*DXPS (100 nM) and IspC (2 μM) were preincubated with varying concentrations of inhibitor in enzyme reaction buffer (100 mM HEPES pH 8, 2 mM MgCl_2_, 5 mM NaCl, 1 mM ThDP, and 200 μM NADPH) for 10 min at 25 °C. The coupled assay was initiated by adding 20 μL of a 10× substrate stock to the preincubated E + I mixture to give a final volume of 200 μL. The final concentration of d-GAP was held constant at 500 μM, and [pyruvate] was added to final concentrations between 0 and 250 μM. The initial rate of NADPH depletion in the IspC-coupled reaction was monitored at 340 nm and was used to calculate the initial rate of DXP formation in the presence or absence of inhibitor. Initial rates were plotted as a function of [pyruvate]. *K*_i_^app^ was determined by nonlinear regression analysis in GraphPad Prism, in which the data were fit to a competitive inhibition model (**18**, **19**, and **21**). For **22**, the data were best fit to a mixed inhibition model. Model discrimination analysis employing the Akaike information criterion was performed as well as Lineweaver–Burk analysis to determine the mixed model as the best fit. In all cases, error bars represent the standard deviation from three replicates.

### Evaluation of IspC Inhibition by Bisubstrate Analog Inhibitors of DXPS

Control experiments were conducted to ensure compounds presented in this study are not inhibiting IspC activity in the coupled assay system. The IspC substrate, DXP, was prepared by incubating DXPS (1 μM) with substrates (4 mM pyruvate and 4 mM d-GAP) in enzyme buffer (2 mM MgCl_2_, 5 mM NaCl, 1 mM ThDP, and 100 mM HEPES pH 8) for 1 h at 37 °C. DXPS was removed using a 10 kDa molecular weight cutoff centrifugal filter. The concentration of DXP in the resulting mixture was quantified using an end point IspC assay. Briefly, 2, 4, 6, and 8 μL of the DXP-containing reaction mixture was incubated with NADPH (200 μM) in 320 μL of reaction buffer (2 mM MgCl_2_ and 100 mM HEPES pH 8) in the presence of IspC (2 μM) for 30 min at 25 °C. The change in absorbance relative to a (−) DXP control was measured and used to determine the concentration of DXP present in the reaction mixture, which was in turn used to calculate the concentration of the DXP stock solution.

Initially, bisubstrate analog inhibitors at concentrations causing ∼90% reduction of the DXPS reaction velocity (as determined by the coupled assay) were evaluated as inhibitors of IspC at a low IspC concentration of 150 nM and in the presence of DXP at 2 × *K*_m_. In this condition, several analogs reduced the initial velocity of IspC-catalyzed NADPH consumption by >50%. As low [IspC] does not reflect the coupled assay condition, where IspC is maintained at a higher concentration of 2 μM to ensure rapid conversion of DXP, inhibitors were re-evaluated using higher [IspC]. In this control, intermediate inhibitor concentrations found to reduce the initial velocity of the DXPS reaction by ∼25–60% in the coupled assay (Figure S10A) were selected, and the DXPS reaction velocities determined from the coupled assay were compared using 2 or 4 μM IspC (DXPS was held constant at 100 nM). Initial velocities determined using 2 or 4 μM IspC were not significantly different (*p* > 0.05 by the Mann–Whitney *t*-test, Figure S10B), indicating that these inhibitors of DXPS do not inhibit IspC under the conditions of the coupled assay. Thus, changes in initial velocity of the DXPS-catalyzed reaction are attributed solely to inhibition of DXPS.

### Molecular Docking Analyses

Molecular docking analyses to rationalize inhibitor potencies were performed using a flexible residue approach in AutoDock Vina.^90,91^ The PLThDP adduct derived from the DXPS-catalyzed reaction between the acetylphosphonate-based bisubstrate analog and the ThDP cofactor was prepared in Chem3D and aligned in the active site of the *Dr*DXPS structure with inhibitor bound to ThDP in its PLThDP form using PyMol (version 2.5.4). AutoDock Tools (version 1.5.7) was used to prepare the E-PLThDP inhibitor structure for docking using a flexible residue approach, where inhibitor and residues *Dr*R423, R480, and K101 were made flexible, and flexible pdbqt files were generated. A docking grid with a size of 18 Å^3^ was used, encompassing the entire DXPS active site. AutoDock Vina was used to perform docking runs on a 6-Core Intel Core i7 computer processor. Nine outputs were generated by AutoDock Vina, the most frequently adopted pose was used to rationalize potencies. PyMol (version 2.5.4) was used to visualize outputs and generate figures.

### Time-Dependent Inhibition of WT *Ec*DXPS by **8** or **10**

*Ec*DXPS (100 nM) and IspC (2 μM) were preincubated in enzyme reaction buffer (100 mM HEPES pH 8, 2 mM MgCl_2_, 5 mM NaCl, 1 mM ThDP, and 200 μM NADPH) with 250 μM d-GAP for 10 min at 25 °C. The reaction was initiated by the addition of pyruvate (1 mM) and inhibitor (400 nM and 800 nM **8**; 1 μM and 100 μM **10**). The reaction mixture was incubated at 25 °C, and the absorbance of NADPH at 340 nm was monitored. The change in the absorbance of NADPH over time was used to calculate [DXP] (μM) produced over time. Experiments were performed in triplicate.

### IC_50_ Determinations for **8** on Wildtype and *Ec*DXPS Variants

*Ec*DXPS and IspC were preincubated with inhibitor for 10 min at 25 °C in assay reaction buffer (100 mM HEPES pH 8, 2 mM MgCl_2_, 5 mM NaCl, 1 mM ThDP, and 200 μM NADPH). Enzyme concentrations for variants were as follows: 200 nM DXPS (wildtype *Ec*DXPS) and 4 μM IspC; 400 nM DXPS (R99A and R478A *Ec*DXPS) and 6 μM IspC. The reaction was initiated by the addition of substrates at final concentrations of 2 × *K*_m_ pyruvate and 10 × *K*_m_d-GAP. NADPH consumption was monitored at 340 nm for 10 min at 25 °C. The initial rate of NADPH consumption was used to determine the initial rate of DXP formation catalyzed by DXPS. Experiments were performed in triplicate and error bars represented standard error.

### Circular Dichroism (CD) to Determine Apparent *K*_D_ of Inhibitors and Examine Persistence of the PLThDP Signal upon Addition of Pyruvate

CD titrations of **8** and **10** onto WT, R99A, and R478A *Ec*DXPS variants were conducted at 25 °C under aerobic conditions in 1.5 mL solutions containing 30 μM *Ec*DXPS in 50 mM HEPES pH 8, 100 mM NaCl, 1 mM MgCl_2_, and 0.2 mM ThDP. Scans were obtained from 280 to 400 nm with a 0.5 s averaging time and 1 nm step following a titration of inhibitor (**8**: 0.25–30 μM on WT, 0.5–30 μM on R99A and R478A; **10** 2.5–30 μM on WT, R99A, and R478A), with a 5 min preincubation of inhibitor with DXPS prior to scanning. The 5 min preincubation was deemed sufficient since no difference in the CD signal was observed with a longer (20 min) preincubation (data not shown). The CD signals at 294 nm determined from the difference spectra obtained by subtracting the signal of E-ThDP at 294 nm were plotted as a function of [inhibitor] and fit to (1) the quadratic equation described in eq 2 (**8** on WT DXPS), where *E* is the concentration of DXPS taking into account stoichiometric binding and CD_max_ is the maximal CD signal, or (2) a one-site specific binding curve (all other experiments) using GraphPad Prism Version 9 to calculate an apparent *K*_D_. Experiments were conducted in triplicate and the standard error was calculated.
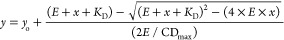
2

At the conclusion of the titration described above, the CD signal at 313 nm (previously characterized for LThDP) was recorded. Pyruvate (1 mM) was then added to the mixture, and a CD scan of the resulting solution was conducted as described above, to detect shifts in the CD spectrum that may correlate to LThDP formation. Experiments were conducted in triplicate.

### Native PAGE to Investigate the DXPS Quaternary Structure

*Ec*DXPS (20 μM) in buffer (2 mM MgCl_2_, 5 mM NaCl, 1 mM ThDP, 100 mM HEPES pH 8) was preincubated with **8** (10, 20, or 3 mM) or no inhibitor for 30 min at 25 °C. Samples were then diluted 2-fold with 2× loading dye (100 mM Tris HCl pH 8, 20% glycerol, 200 mM dithiothreitol (DTT), 0.01% bromophenol blue). Samples (15 μL) were then loaded on a 7.5% PAGE gel. Gel electrophoresis was conducted at 4 °C for 30 min at 120 V, followed by 3 h at 150 V. Protein bands were stained with Protostain Blue. Experiments were performed in triplicate.

### Assessment of DXPS Oligomerization State in the Presence or Absence of 8 Using Analytical Gel Filtration Column Chromatography

Analytical gel filtration was performed on an AKTA-GO FPLC system held at 4 °C fitted with a Cytiva Superdex 200 Increase 5/150 GL column. A 200 μL solution containing 29 μM *Ec*DXPS in 25 mM HEPES, pH 8, 100 mM NaCl, 1 mM MgCl2, 1% glycerol, 10 μM ThDP, and 29 μM **8** or no inhibitor was centrifuged at 13,000 rpm for 10 min to remove particulates. The resulting supernatant was loaded into a 10 μL loop attached to the FPLC via gastight syringes. The solution was loaded onto the column by flowing 50 μL of running buffer (25 mM HEPES, pH 8, 100 mM NaCl, 1 mM MgCl_2_, 1% glycerol, 10 μM ThDP) through the loop. DXPS was analyzed by isocratic elution from the column at a flow of 0.15 mL/min with 1.5 column volumes (4.5 mL). The dimer/tetramer ratio was calculated from the peak area (mL mAu) of the dimer (1.7 mL retention) peak divided by the peak area (mL mAu) of the tetramer (1.4 mL retention) peak. Experiments were conducted in triplicate. Error bars represent standard error. Statistical significance testing of differences in the dimer/tetramer ratios was conducted using an unpaired *t*-test with Welch correction in GraphPad Prism version 9. A *p*-value <0.05 indicates statistical significance.

## Data Availability

Coordinates for the crystal structure of *Dr*DXPS bound to **1** have been deposited to the Protein Data Bank PDB ID 8V9I.

## Supporting Information Available

Data collection and refinement statistics and additional figures of crystal structure of *Dr*DXPS bound to **1**; kinetics of DXP formation on WT, R99A, and R478A *Ec*DXPS; determination of *K*_i_^app^ values; molecular docking analyses; inhibitory activity of **8** against PDH; reaction progress curves for DXP formation by WT DXPS and variants in the presence or absence of **8** or **10**; CD titrations of **8** and **10** on WT DXPS and variants; CD analysis of PLThDP from **8** in the presence or absence of pyruvate on WT DXPS and variants; IspC inhibition by bisubstrate analogs; synthesis of diazotransfer reagent, hpAP, and general procedure for the synthesis of azide precursors and inhibitors; inhibitor purities; compound characterization, including ^31^P and ^1^H NMR, HPLC, and HRMS; and oligomerization of DXPS in the presence or absence of **1** or **8** (PDF)
PDB validation report (PDF)

## Abbreviations

DXPS: 1-deoxy-d-xylulose 5-phosphate synthase
d-GAP: d-glyceraldehyde 3-phosphate
ThDP: thiamin diphosphate
LThDP: C2α-lactylthiamin diphosphate
PLP: pyridoxal phosphate
DMADP: dimethylallyl diphosphate
IDP: isopentenyl diphosphate
PDH: pyruvate dehydrogenase
MAP: methylacetylphosphonate
PLThDP: phosphono-lactylthiamin diphosphate
IspC: 1-deoxy-d-xylulose 5-phosphate reductoisomerase
CD: circular dichroism
AP: 4′-aminopyrimidine tautomer of ThDP
IP: 1′,4′-iminopyrimidine tautomer of ThDP
AlkylAPs: alkyl acetylphosphonates
TrAP: triazoleAP
MEP: methylerythritol phosphate
PPP: pentose phosphate pathway
*Ec*: *Escherichia coli*
*Dr*: *Deinococcus radiodurans*
hpAP: homopropargylAP
CuAAC: copper(II)-catalyzed azide–alkyne cycloaddition
IspC: DXP reductioisomerase
FPLC: fast protein liquid chromatography
HEPES: 4-(2-hydroxyethyl)-1-piperazineethanesulfonic acid
HRMS: high-resolution mass spectrometry
TCEP: tris(2-carboxyethyl)phosphine
TCA: tricarboxylic acid
BCAA: branched-chain amino acid
