# Supplementary material for: Potent Inhibition of E. coli DXP Synthase by a gem-Diaryl Bisubstrate Analog
Source: ACS Infect Dis. 2024 Mar 21;10(4):1312–26. doi: 10.1021/acsinfecdis.3c00734 (PMC11019550; doi:10.1021/acsinfecdis.3c00734)
Supplement: Supplementary file 1 — id3c00734_si_001.pdf [file id3c00734_si_001.pdf]

Supporting Information

Potent inhibition of *E. coli* DXP synthase by a gem-diaryl bisubstrate analog

Lauren B. Coco[†§], Eucolona M. Toci[†§], Percival Yang-Ting Chen[‡], Catherine L. Drennan[‡ǁ#], and Caren L. Freel Meyers[†#]

[†]Department of Pharmacology and Molecular Sciences, Johns Hopkins University School of Medicine, Baltimore, Maryland 21205, United States.

[§]These authors contributed equally.

[‡]Department of Chemistry, Massachusetts Institute of Technology, Cambridge, MA 02139, USA.

[ǁ]Howard Hughes Medical Institute, Department of Biology, Massachusetts Institute of Technology, Cambridge, MA 02139, USA

[#]Corresponding authors Caren Freel Meyers, cmeyers@jhmi.edu; Catherine L. Drennan, cdrennan@mit.edu

Table of Contents

Table S1. Data collection and model refinement statistics for the structure of *Dr*DXPS bound to PLThDP formed in the presence of 1 .... S2

Table S2. Residues and cofactors modeled in each chain (1-629) of the structure of *Dr*DXPS bound to PLThDP formed in the presence of 1 .... S3

Table S3. Kinetics of DXP formation on WT, R99A, and R478A DXPS S3

Figure S1. Comparing active sites of *Dr*DXPS with PLThDP adducts of MAP and 1 bound S4

Figure S2. Determination of active [E] using 8 and K_i^{app} by the Morrison equation for 1-7, 10-17 and 20 against WT DXPS S5

Figure S3. Molecular docking analyses of TrAP analogs into the *Dr*DXPS active site S6

Figure S4. Determination of apparent K_i values S7

Figure S5. Evaluation of inhibitory activity of 8 against mammalian PDH S8

Figure S6. Replicate progress curves for DXP formation in the presence or absence of inhibitors 8 or 10 .... S8

Figure S7. Representative CD titrations of 1, 8 and 10 on WT DXPS and variants S9

Figure S8. Replicates of reaction progress curves for DXP formation on R99A in the presence or absence of 8 .... S10

Figure S9. Representative CD traces illustrating behavior of the CD signal corresponding to PLThDP formed from 8 on WT and variants S10

Figure S10. Evaluation of IspC inhibition by bisubstrate analog inhibitors of DXPS S11

Figure S11. Analytical gel filtration of WT DXPS in the presence and absence of 8 .... S11

Synthesis of diazotransfer reagent S12

Synthesis of hpAP. S12

General procedure for synthesis of azides. S12

General procedure for synthesis of triazole acetylphosphonate (TrAP) inhibitors S13

Determination of inhibitor purity S13

Compound Characterizations. S13

^{31}P and ^{1}H NMR, HPLC, and HRMS for bisubstrate analog inhibitors S17

References S59

Table S1. Data collection and model refinement statistics for the structure of *Dr*DXPS bound to PLThDP formed in the presence of **1**.

	*Dr*DXPS with D-PheTrAP bound
Beamline	APS 24-ID-C
Space group	$P2_12_12_1$
Cell dimensions (Å)	a = 78.62, b = 125.32, c = 152.02
Wavelength (Å)	0.9791
Resolution (Å) †	100.-1.98 (2.05-1.98)
# unique reflections	103920
Completeness (%) †	99.1 (92.2)
Redundancy †	6.3 (3.9)
**<I/σI>** †	19.4 (2.1)
R_{sym} †	0.092 (0.619)
$CC_{1/2}$ †	(0.808)
Resolution (Å)	66.6-1.98
# unique reflections	103784
R_{work} (%) / R_{free} (%)	16.0/18.1
RMS bond lengths (Å)	0.003
RMS bond angles (°)	0.62
Number of Atoms/Molecules	
Protein atoms	8935
**1**-ThDP	2
Water molecules	580
Na^+	2
1,2,4-butanetriol	1
Average B-factor (Å^2)	40.8
Protein atoms	40.8
**1**-TTP	35.9
Water molecules	40.5
Na^+	31.3
1,2,4-buatnetriol	60.5
Ramachandran plot	
Favored (%)	97.16
Allowed (%)	2.84
Outliers (%)	0.00
Rotamer outliers (%)	0.65

†Values in parentheses indicate the highest-resolution bin.

Table S2. Residues and cofactors modeled in each chain (1-629) of the structure of *Dr*DXPS bound to PLThDP formed in the presence of **1**.

Chain	*Dr*DXPS with D-PheTrAP (**1**) bound
**A**	Residues 8-208, 217-224, 244-629<br>1 **1**-ThDP, 1 Na^+<br>1 1,2,4-butanetriol
**B**	Residues 8-200, 244-626<br>1 **1**-ThDP, 1 Na^+

Table S3. Characterization of the kinetics of DXP formation on WT, R99A, and R478A *Ec*DXPS. Error represents standard error, where $n = 3$. WT, wild-type. [a] Johnston *et al.*[1]

DXPS	K_m^{Pyr} (μM)	K_m^{GAP} (μM)	k_{cat} (s^{-1})	k_{cat}/K_m^{Pyr} ($μM^{-1} s^{-1}$)	k_{cat}/K_m^{GAP} ($μM^{-1} s^{-1}$)
WT	21.6 ± 0.9	14 ± 2	1.00 ± 0.03	(2.910 ± 0.002) x 10^{-2}	(7 ± 1) x 10^{-2}
R99A	39 ± 2	120 ± 30	0.40 ± 0.02	(1.03 ± 0.08) x 10^{-2}	(3.5 ± 0.8) x 10^{-3}
R478A	42 ± 3 [a]	1400 ± 200 [a]	119 ± 20 [a]	(4.3 ± 0.3) x 10^{-2} [a]	(1.7 ± 0.2) x 10^{-3} [a]

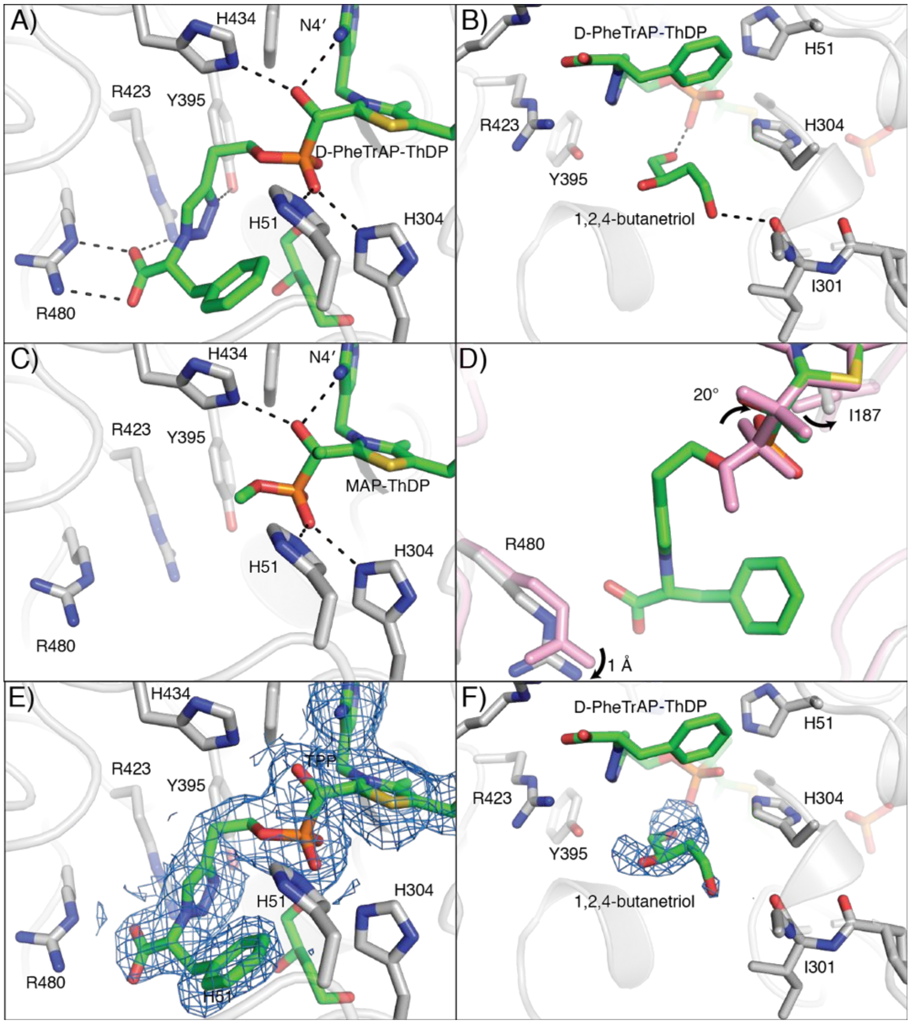

Figure S1. Comparing active sites of *Dr*DXPS with PLThDP adducts of MAP and **1** bound & composite omit electron density maps of **1** (D-PheTrAP) and 1,2,4-butantriol. **A**) Structure of E-PLThDP from incubation and crystallization of *Dr*DXPS in the presence of **1**. The phosphonolactyl moiety is stabilized by the same set of interactions as in the E-PLThDP structure from crystrallization of DXPS with MAP (panel C). **B**) 1,2,4-Butanetriol, from the crystallization condition, is also bound in the active site of the structure of *Dr*DXPS with **1** bound. 1,2,4-butanetriol forms hydrogen bonds with the phosphonate of **1** and the backbone of I301. **C**) Structure of E-PLThDP from incubation and crystallization of *Dr*DXPS in the presence of MAP (PDB code 6OUV). The phosphonate moiety forms salt bridges with H51 and H304; H434 and N4′ of ThDP stabilize the hydroxyl moiety. **D**) The steric effect of D-PheTrAP (**1**) expands the active site. The expansion shifts the guanidine moiety of R480 away from the active site by 1 Å, and the side chain of I187 is also in a different rotamer to prevent clashing with the phosphonate. The phosphonate moiety is rotated by 20° by the steric effect of the D-Phe triazole moiety. **E**) Composite omit map contoured to 1.0σ in blue mesh for the E-PLThDP adduct formed from incubation of DXPS with **1**. **F**) Composite omit map contoured to 1.0σ in blue mesh for 1,2,4-butanetriol in the structure of DXPS with **1** bound. In panels **A-C, E,** and **F**, amino acids in

*Dr*DXPS are colored gray; ThDP adducts are colored green. In panel **D**, the structure of *Dr*DXPS with **1** bound is in the same color scheme as panel **A-C**; the structure of *Dr*DXPS with MAP bound is colored pink.

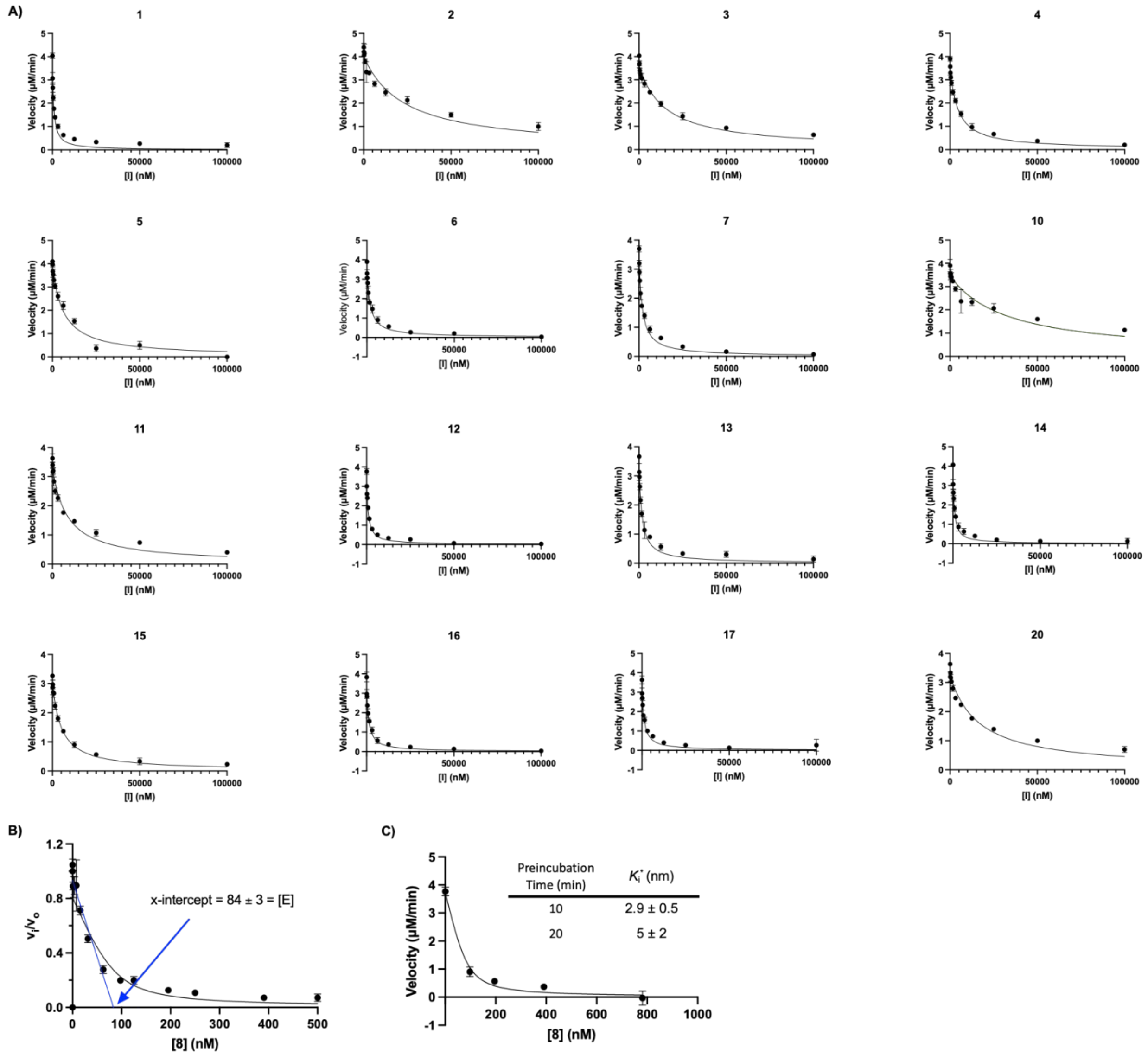

Figure S2. Determination of apparent K_i by the Morrison equation for **1-7**, **10-17** and **20** against WT *Ec*DXPS. **A**) Morrison curve fits. **B**) Determination of active [E] for the Morrison equation following Copeland's method using **8**.[2] **C**) Morrison curve fit for **8** against WT *Ec*DXPS following a 20 min preincubation of **8** with WT *Ec*DXPS. K_i^* values determined following a 10 min or 20 min preincubation are comparable. Error bars represent standard error from three replicates.

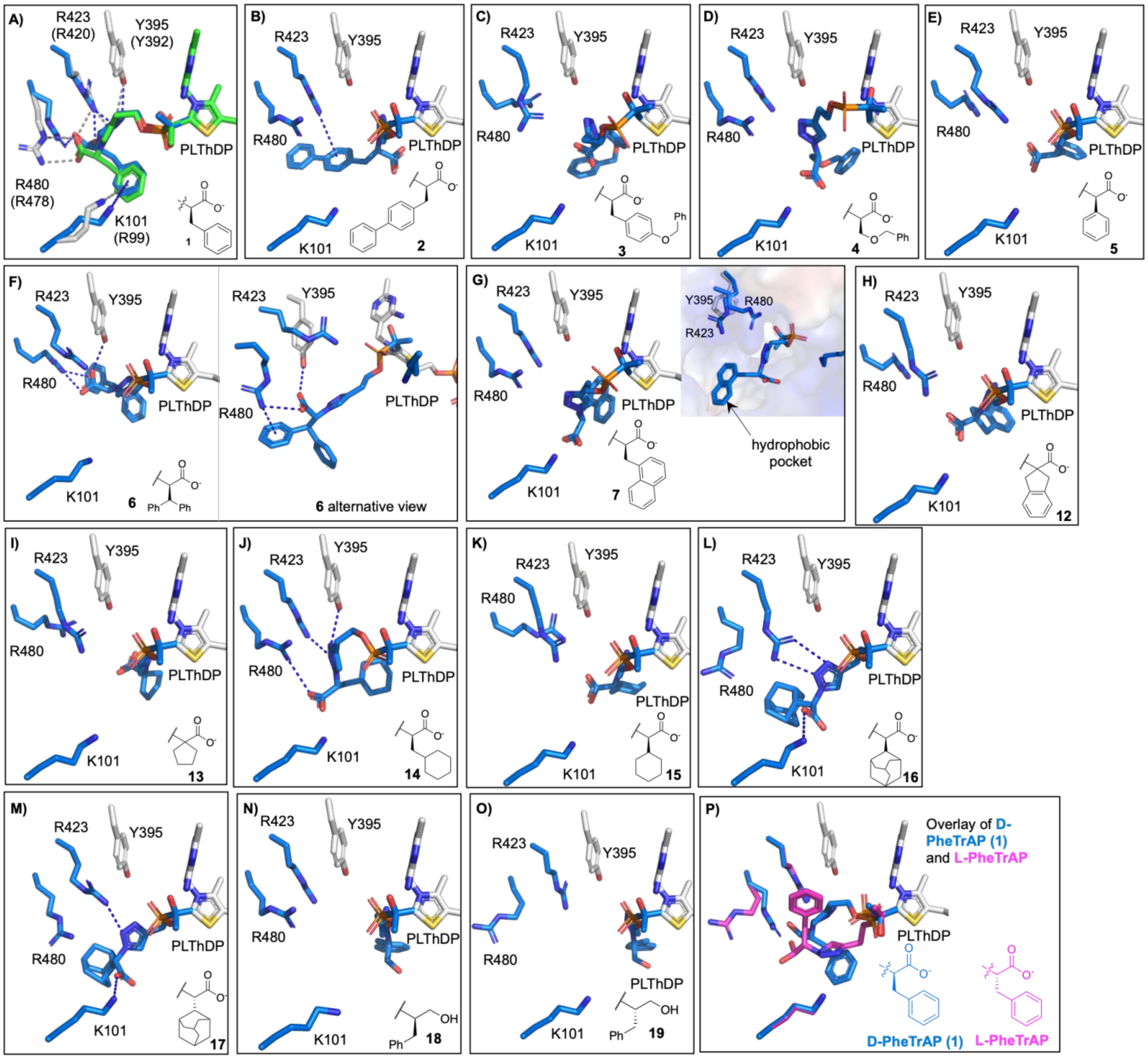

Figure S3. Molecular docking analyses of TrAP analogs into the *Dr*DXPS active site of the *Dr*DXPS-**1** crystal structure using a flexible residues approach in AutoDock Vina. Residue contacts and intramolecular interactions are indicated by dashed blue lines (hydrogen bond interactions ≤ 3.5 Å and π-based interactions ≤ 6 Å). Numbers indicate *D. radiodurans* (*E. coli*) DXPS residues. In panel **A**) docked PLThDP of **1** (blue) shows similar orientation and residue contacts (docked: blue dashed lines; crystal: grey dashed lines) as the crystal pose (residues colored grey, PLThDP colored green). Throughout, blue coloring indicates structures allowed flexibility in the docking simulation (inhibitor and residues *Dr*R423, R480, and K101). Structures colored

light grey were not allowed flexibility in the docking simulation. In panel **P**) structures allowed flexibility in the docking simulation of L-PheTrAP are colored pink.

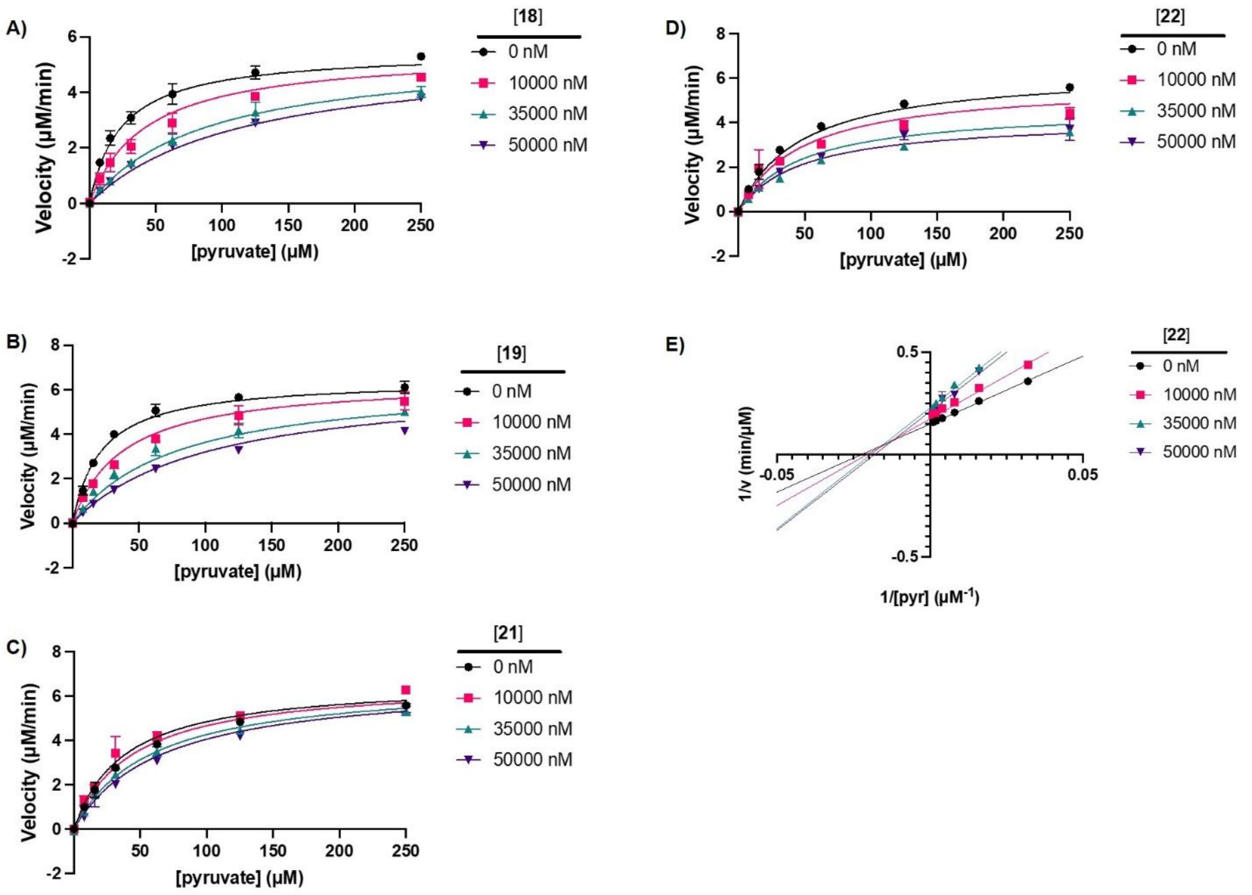

Figure S4. Determination of apparent K_i values of **18, 19, 21,** and **22** with *Ec*DXPS. Data were analyzed in GraphPad Prism and fit to a competitve model for **18** and **19 (A, B**) or mixed inhibition model for **22**. (**C**) Data for **21** were fit to a competitive model, although neglible inhibition was oberserved. (**D**). Double reciprocal plot of kinetic data for **22**, (**E**) supports a mixed inhibition mode. Error bars represent standard error from three replicates.

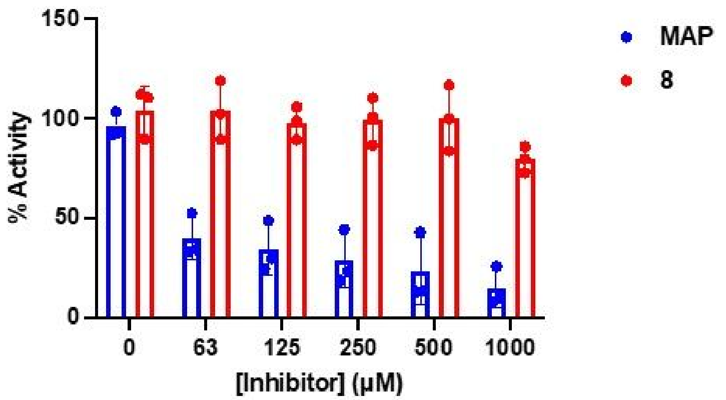

Figure S5. Evaluation of inhibitory activity of **8** against mammalian porcine pyruvate dehydrogenase (PDH). Porcine PDH (0.01 U/mL) and inhibitor (**8** or MAP positive control) were preincubated for 10 minutes at 25 ºC in enzyme buffer (2 mM $MgCl_2$, 5 mM L-cysteine, 1 mM ThDP, 300 μM TCEP, 100 mM HEPES pH 8, 2.5 mM NAD^+, and 100 μM coenzyme A). The reaction was initiated by addition of pyruvate (60 μM). The change in absorbance at 340 nm over time was measured and used to calculate the initial rate of NADH formation. The % activity of PDH in the presence of **8** or MAP was plotted using GraphPad Prism version 9. There is 21% inhibition of PDH activity observed in the presence of compound **8** at a concentration of 1 mM. Experiments were performed in triplicate. Error bars represent standard error.

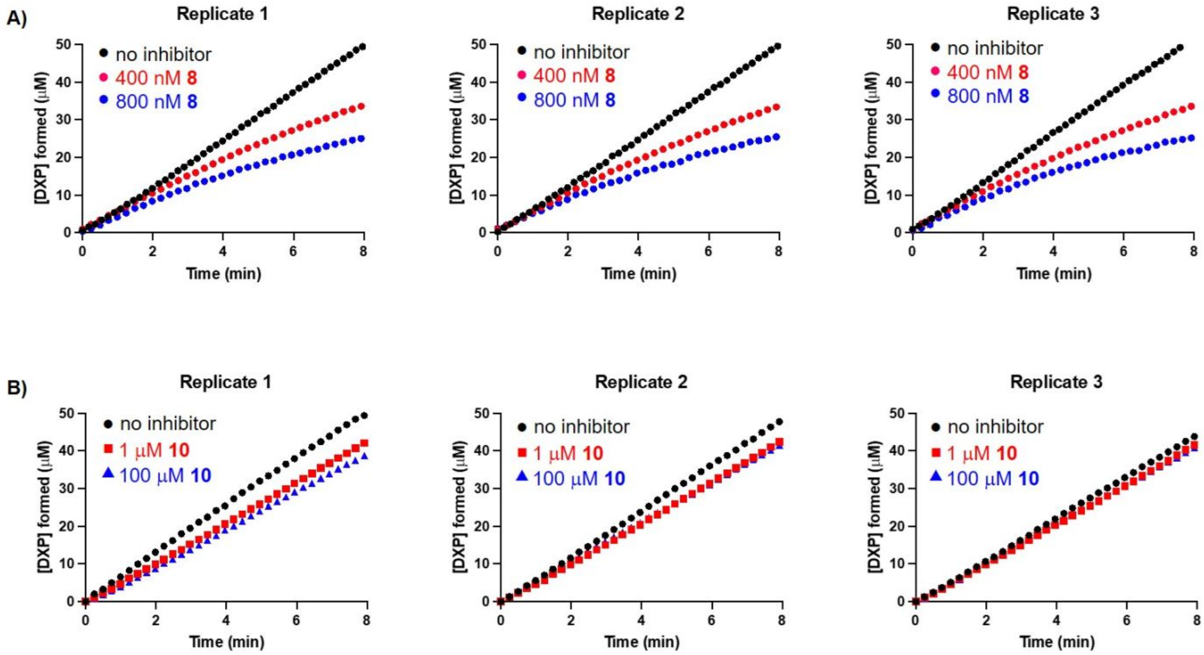

Figure S6. Replicate progress curves for DXP formation in the presence or absence of inhibitors **8** or **10** with *Ec*DXPS. **A**) Progress curves for formation of DXP in the presence of **8** display time-dependence of inhibition. **B**) Progress curves for formation of DXP in the presence of descarboxyl analog **10** are linear.

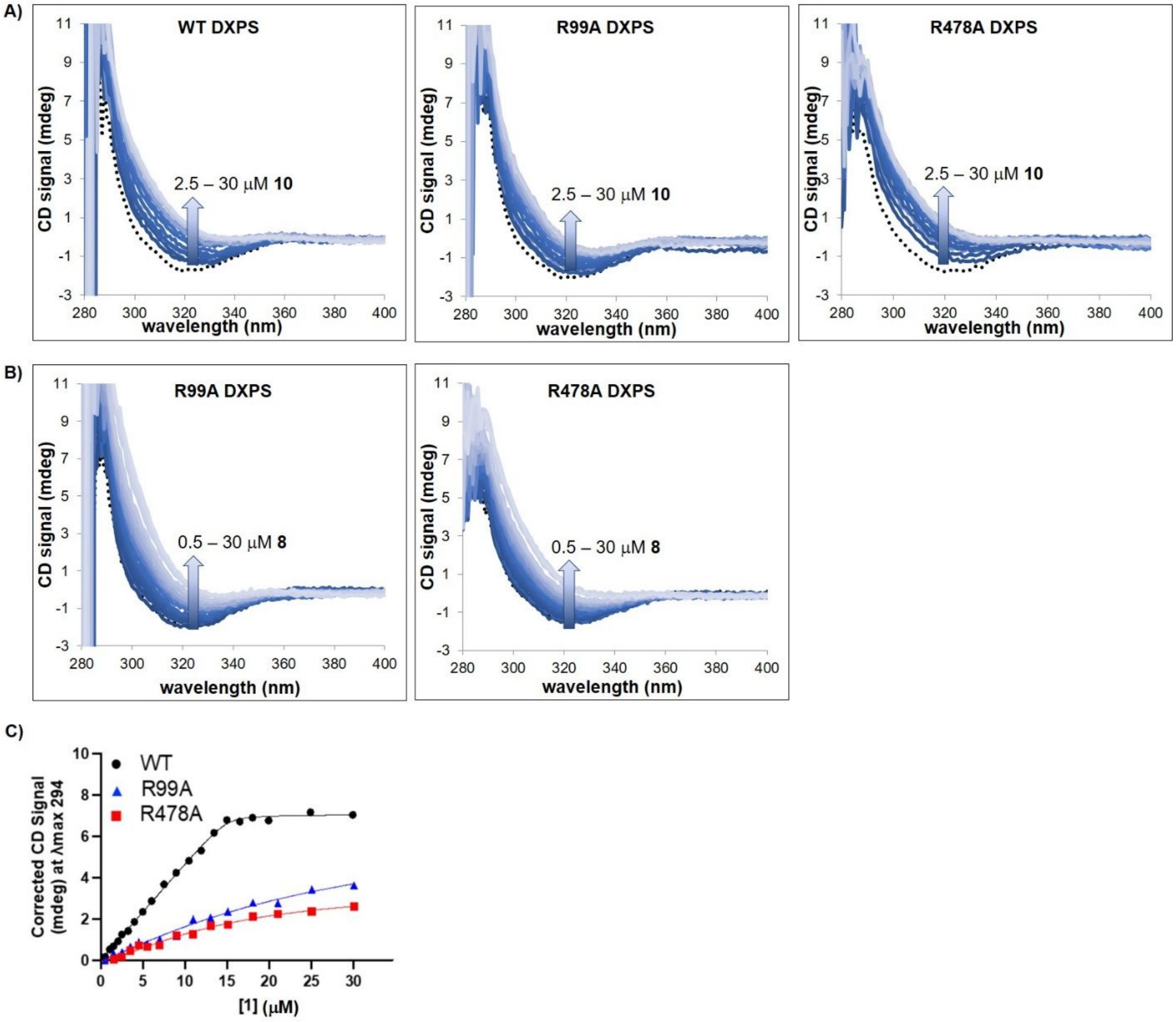

Figure S7. Representative CD titrations of **1, 8** and **10** on WT *Ec*DXPS and variants showing formation of CD signal corresponding to PLThDP. **A**) Titration of **10** onto WT (left), R99A (middle), and R478A (right) *Ec*DXPS, n = 3. **B**) Titration of **8** onto R99A (left) and R478A, n = 3. **C**) Representative titrations of **1** onto WT *Ec*DXPS, R99A and R478A DXPS showing lower affinity of PLThDP on R99A and R478A relative to wildtype.

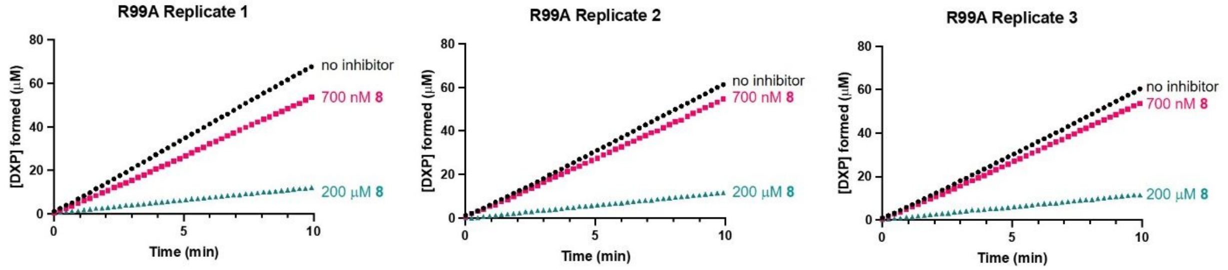

Figure S8. Replicates of reaction progress curves for DXP formation on *Ec*R99A in the presence or absence of **8**. Reactions were initiated by simultaneous addition of substrates (780 μM pyruvate and 2400 μM D-GAP) in the presence or absence of **8** (700 nM or 200 μM) at 25 °C. Reaction progress was measured using the DXPS-IspC coupled assay. Experiments were performed in triplicate as shown.

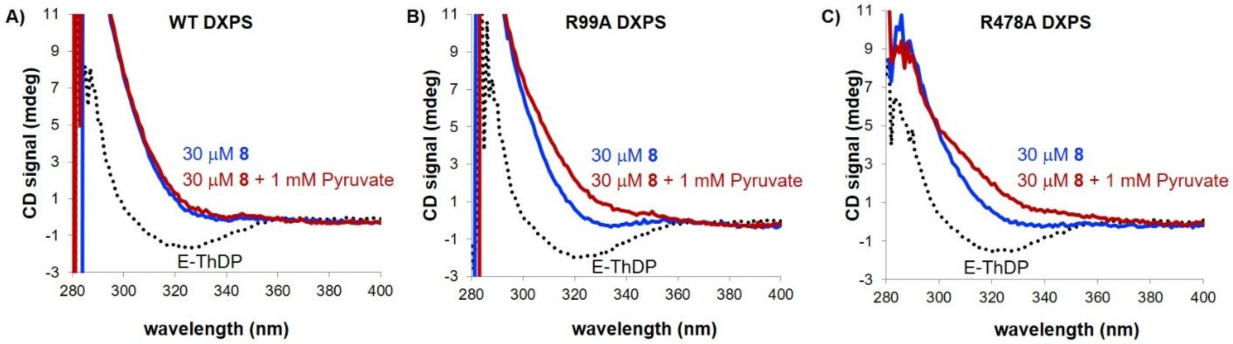

Figure S9. Representative CD traces illustrating behavior of the CD signal corresponding to PLThDP formed from **8** on WT and *Ec*DXPS variants. **A**) CD traces showing lack of a significant shift in CD signal following addition of pyruvate (1 mM, red trace) to E-PLThDP formed from **8** (blue trace) on WT *Ec*DXPS. **B**) Observed shift toward a LThDP-like CD signal following addition of pyruvate to E-PLThDP formed from **8** on *Ec*R99A DXPS. **C**) Observed shift toward a LThDP-like CD signal following addition of pyruvate to E-PLThDP formed from **8** on *Ec*R478A DXPS.

A)

Inhibitor	[I] (μM)	Inhibitor	[I] (μM)
**1**	0.8	**13**	1.6
**2**	25	**14**	0.8
**3**	12.5	**15**	6.3
**4**	3.1	**16**	0.8
**5**	6.3	**17**	0.8
**6**	1.6	**18**	50
**7**	1.6	**19**	50
**8**	0.031	**20**	12.5
**10**	25	**21**	50
**11**	6.3	**22**	50

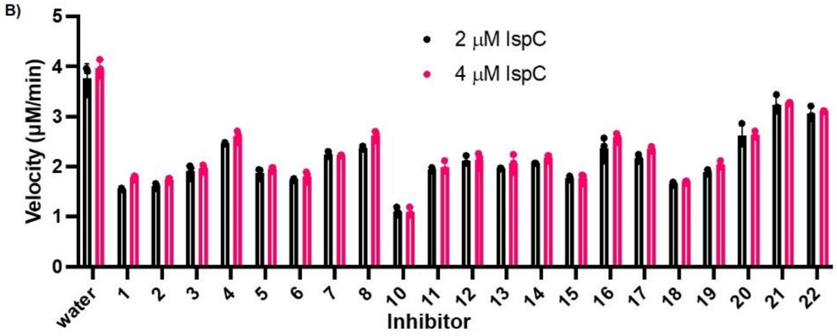

Figure S10. Evaluation of IspC inhibition by bisubstrate analog inhibitors of DXPS. **A**) Inhibitor concentrations used to detect inhibitory activity of bisubstrate analogs against IspC in the DXPS-IspC coupled assay. **B**) DXPS reaction rates determined from the coupled assay, using 100 nM *Ec*DXPS and 2 mM or 4 mM IspC. Initial velocities determined using 2 mM or 4 mM IspC were not significantly different ($p > 0.05$ by the Mann-Whitney t-test). Error bars represent standard error from three replicates.

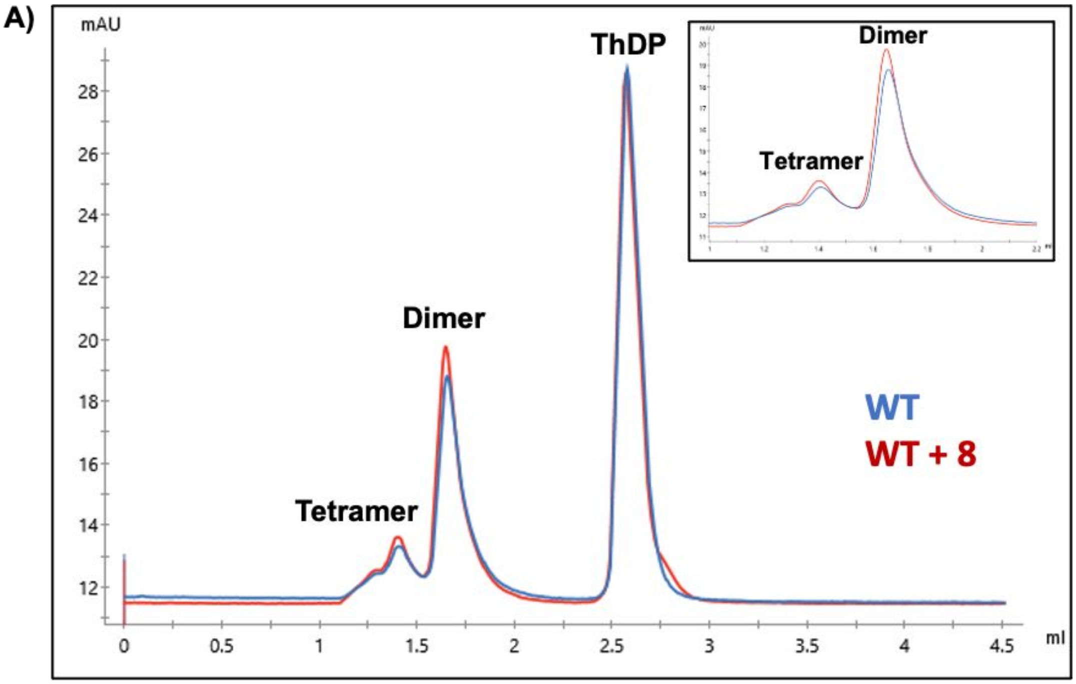

B)

	Dimer (mL*mAU)	Tetramer (mL*mAU)	Dimer:Tetramer ratio
**WT**	1.14 ± 0.03	0.34 ± 0.02	3.4 ± 0.2
**WT + 8**	1.28 ± 0.02	0.45 ± 0.03	2.8 ± 0.2

Figure S11. Analytical gel filtration of WT *Ec*DXPS in the presence or absence of **8**. **A**) Representative analytical gel filtration traces monitoring the dimeric and tetrameric DXPS elution region for WT alone (blue) and WT + **8** (red). **B**) Table indicated peak areas associated with tetramer and dimer peak elutions. Error represents standard error, where $n = 3$.

Synthesis of diazotransfer reagent. The diazotransfer reagent was prepared as previously reported.[3] White solid, 41% yield.

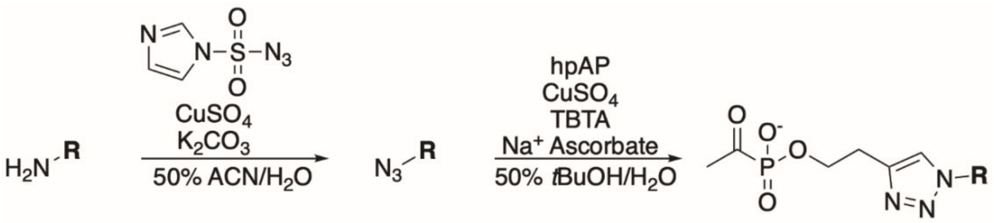

Synthesis of homopropargylAP (hpAP). The common alkyne intermediate hpAP was prepared by either of two routes. Preparation of hpAP from PCl_3 and homopropargyl alcohol was conducted according to our previous report,[4] based on work by Fang, *et al.*[5] A higher yielding, more reproducible synthesis of hpAP was developed using phosphormidite-based chemistry. Briefly, to a flame-dried flask charged argon was added 3-butyn-1-ol (14.3 mmol, 1 eq, 1.0 g). Tetrazole was added dropwise (14.3 mmol, 1 eq, 31.8 mL of 0.45 M in acetonitrile) followed by slow dropwise addition of dimethyl-*N*,*N*-diisopropylphosphoramidite (14.3 mmol, 1 eq, 3.29 mL). The reaction mixture stirred for 90 minutes at ambient temperature under argon. Dichloromethane (50 mL) was added, and salts were removed by vacuum filtration. The filtrate was washed three times with aqueous NaOH (1 M, 50 mL) then dried over $MgSO_4$, filtered, and concentrated under vacuum. The but-3-yn-1-yl dimethyl phosphite was used without further purification or characterization.

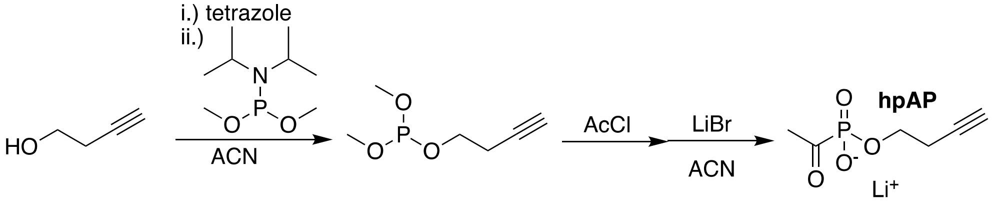

Acetyl chloride (67.9 mmol, 5 eq, 4.8 mL) was added to a flame-dried flask charged with argon. But-3-yn-1-yl dimethyl phosphite (13.6 mmol, 1 eq) was then added dropwise very slowly by syringe. The reaction mixture was stirred for 30 minutes at ambient temperature, and then excess acetyl chloride was removed under vacuum. The remaining oil was dissolved in anhydrous acetonitrile (13.6 mL). Lithium bromide (13.6 mmol, 1 eq 1.181 g, stored at 130 °C) was quickly weighed and added in one portion. The reaction mixture was stirred at ambient temperature under argon overnight. The product, hpAP, precipitated and was isolated via vacuum filtration, washed with acetonitrile, and dried under vacuum.

General procedure for synthesis of azides. Amine or amino acid (1 mmol, 1 eq) was suspended in 4 mL 50% acetonitrile/water. Potassium carbonate (3.2 mmol, 3.2 eq, 441 mg), copper (II) sulfate pentahydrate (0.1 mmol, 0.1 eq, 25 mg), and diazotransfer reagent (1.2 mmol, 1.2 eq, 325 mg) were added to the suspension in that order. The reaction mixture was covered loosely and allowed to stir overnight at room temperature. The reaction mixture was acidified with concentrated HCl (5 mL) and extracted with ethyl acetate (3 × 50 mL). The organic layers were combined, dried over $MgSO_4$, filtered, and concentrated under vacuum. The azides were used

without further characterization assuming full conversion (100 %) of the amine to azide, although % conversion was not empirically determined.

General procedure for synthesis of triazole acetylphosphonate (TrAP) inhibitors. Azide (1 mmol, 1 eq) and hpAP (0.5 mmol, 0.5 eq, 88 mg) were dissolved in 4 mL 50% *tert*-butanol/water. Copper (II) sulfate hexahydrate (0.05 mmol, 0.05 eq, 12 mg), and tris((1-benzyl-4-triazolyl)methyl)amine (TBTA, 0.1 mmol, 0.1 eq, 53 mg) were added to the solution in that order. The headspace of the reaction vessel was flushed with argon. Then, sodium ascorbate (0.2 mmol, 0.2 eq, 40 mg) was added in one portion, and the reaction vessel was immediately capped tightly. The reaction proceeded overnight at room temperature with vigorous stirring. The reaction mixture was then flash frozen and lyophilized to dryness. The crude powder was redissolved in a minimal amount of water and purified by C18 flash chromatography (50 mM triethylammonium acetate (TEAA) pH 6 and acetonitrile). Compounds eluted between 20-40% 50 mM TEAA:acetonitrile. CuAAC reactions were not optimized for individual inhibitors, and yields ranged from 5% to 37%. This was due to incomplete consumption of hpAP in some cases.

Determination of inhibitor purity. Reversed phase (RP) HPLC was used to determine purity of each new DXPS inhibitor (compounds **2**-**8**, **10**-**22**) presented in this study. A fresh 1 mM solution of inhibitor in water prepared immediately prior to injection. Then, 5 uL of the 1 mM inhibitor solution was injected onto a ZORBAX 80Å extend-C18 column (4.6 x 50 mm, 3.5 µm) with a flow rate of 1 mL/min. Mobile phases used include water + 0.1% trifluoroacetic acid (solvent A) and acetonitrile (solvent B). The following method was used: 30 seconds at 5% B, ramp to 95% B over 4 minutes, and hold 95% B for 54 seconds. Compounds were evaluated at 235 nm, unless otherwise indicated, using the HPLC diode array detector (DAD).

Compound Characterizations.

But-3-yn-1-yl acetylphosphonate (lithium salt) (hpAP). ^{1}H NMR (500 MHz, D_2O) δ 3.99 (dt, *J* = 7.6, 6.3 Hz, 2H), 2.54 (td, *J* = 6.3, 2.7 Hz, 2H), 2.42 (d, *J* = 4.7 Hz, 3H), 2.36 (t, *J* = 2.6 Hz, 1H). 31P NMR (202 MHz, D_2O) δ -26.73. HRMS (ESI-TOF) m/z calculated for $C_6H_8O_4P$ [M-H]$^-$ 175.0166; found 175.0160. White solid, 92% yield (over 3 steps).

(*R*)-3-([1,1'-biphenyl]-4-yl)-2-(4-(2-((acetyloxidophosphoryl)oxy)ethyl)-1*H*-1,2,3-triazol-1-yl)propanoate 2(triethylammonium) (**2**). ^{1}H NMR (500 MHz, MeOD) δ 7.92 (s, 1H), 7.47 (d, J = 7.7 Hz, 2H), 7.37 (d, J = 7.8 Hz, 2H), 7.31 (t, J = 7.6 Hz, 2H), 7.20 (t, J = 7.4 Hz, 1H), 7.11 (d, J = 7.9 Hz, 2H), 5.24 (dd, J = 10.9, 4.5 Hz, 1H), 4.12 – 3.92 (m, 1H), 3.64 – 3.32 (m, 3H), 3.05 (q, J = 7.3 Hz, 21H), 2.95 (t, J = 6.9 Hz, 2H), 2.24 (d, J = 4.0 Hz, 3H), 1.19 (t, J = 7.3 Hz, 31H). ^{31}P NMR (202 MHz, MeOD) δ -26.35. HRMS (ESI-TOF) m/z calculated for $C_{21}H_{20}N_3O_6P$ [M-H]$^-$ 441.1101; found 442.1163. Light yellow oil, 7% yield.

(*R*)-2-(4-(2-((acetyloxidophosphoryl)oxy)ethyl)-1*H*-1,2,3-triazol-1-yl)-3-(4-(benzyloxy)phenyl)propanoate 2(triethylammonium) (**3**). ^{1}H NMR (500 MHz, D_2O) δ 7.83 (s, 1H), 7.49 – 7.39 (m, 5H), 7.03 (d, J = 8.2 Hz, 2H), 6.89 (d, J = 8.2 Hz, 2H), 5.34 – 5.29 (m, 1H), 5.11 (s, 2H), 4.05 (q, J = 6.6 Hz, 2H), 3.61 – 3.30 (m, 2H), 3.20 (q, J = 7.3 Hz, 13H), 2.99 (t, J = 6.5 Hz, 2H), 2.25 (d, J = 4.6 Hz, 2H), 1.28 (t, J = 7.3 Hz, 18H). ^{31}P NMR (202 MHz, D_2O) δ -

26.84. HRMS (ESI-TOF) m/z calculated for $C_{22}H_{22}N_3O_7P$ $[M-H]^-$ 471.1206; found 472.1269. Light brown oil, 6% yield.

(*R*)-2-(4-(2-((acetyloxidophosphoryl)oxy)ethyl)-1*H*-1,2,3-triazol-1-yl)-3-(benzyloxy)propanoate 2(triethylammonium) (**4**). ^{1}H NMR (500 MHz, D_2O) δ 7.96 (s, 1H), 7.44 (dd, *J* = 5.0, 2.8 Hz, 3H), 7.36 – 7.26 (m, 2H), 5.50 (dd, *J* = 8.6, 4.2 Hz, 1H), 4.71 – 4.51 (m, 2H), 4.33 – 4.24 (m, 2H), 4.20 (q, *J* = 6.6 Hz, 1H), 3.25 (qd, *J* = 7.3, 1.8 Hz, 11H), 3.13 (t, *J* = 6.3 Hz, 2H), 2.35 (dd, *J* = 4.7, 1.8 Hz, 2H), 1.33 (td, *J* = 7.4, 1.8 Hz, 16H). ^{31}P NMR (202 MHz, D_2O) δ -26.34. HRMS (ESI-TOF) m/z calculated for $C_{16}H_{19}N_3O_7P$ $[M-H]^-$ 396.0966; found 396.0967. Light yellow oil, 22% yield.

(*R*)-2-(4-(2-((acetyloxidophosphoryl)oxy)ethyl)-1*H*-1,2,3-triazol-1-yl)-2-phenylacetate 2(triethylammonium) (**5**). ^{1}H NMR (500 MHz, D_2O) δ 7.73 (s, 1H), 7.49 – 7.43 (m, 5H), 6.31 (s, 1H), 4.06 (q, *J* = 6.2 Hz, 1H), 3.17 (qd, *J* = 7.3, 1.1 Hz, 13H), 3.00 (t, *J* = 6.1 Hz, 2H), 2.13 (dd, *J* = 4.6, 1.1 Hz, 2H), 1.25 (td, *J* = 7.4, 1.1 Hz, 19H). ^{31}P NMR (202 MHz, D_2O) δ -26.52. HRMS (ESI-TOF) m/z calculated for $C_{14}H_{14}N_3O_6P$ $[M-H]^-$ 351.0631; 352.0698. Light brown oil, 7% yield.

(*R*)-2-(4-(2-((acetyloxidophosphoryl)oxy)ethyl)-1*H*-1,2,3-triazol-1-yl)-3,3-diphenylpropanoate 2(triethylammonium) (**6**). ^{1}H NMR (500 MHz, D_2O) δ 8.04 (s, 1H), 7.62 – 7.56 (m, 2H), 7.46 (t, *J* = 7.7 Hz, 2H), 7.40 – 7.33 (m, 3H), 7.27 (dd, *J* = 8.4, 6.8 Hz, 2H), 7.24 – 7.15 (m, 1H), 5.97 (d, *J* = 12.2 Hz, 1H), 4.95 (d, *J* = 12.3 Hz, 1H), 4.06 (q, *J* = 6.5 Hz, 2H), 3.24 (q, *J* = 7.4 Hz, 14H), 2.98 (t, *J* = 6.4 Hz, 2H), 2.29 (d, *J* = 4.6 Hz, 2H), 1.32 (t, *J* = 7.3 Hz, 19H). ^{31}P NMR (202 MHz, D_2O) δ -26.61. HRMS (ESI-TOF) m/z calculated for $C_{21}H_{20}N_3O_6P$ $[M-H]^-$ 441.1101; 442.1168. Brown oil, 13% yield.

(*R*)-2-(4-(2-((acetyloxidophosphoryl)oxy)ethyl)-1*H*-1,2,3-triazol-1-yl)-3-(naphthalen-1-yl)propanoate 2(triethylammonium) (**7**). ^{1}H NMR (500 MHz, D_2O) δ 8.04 (d, *J* = 8.5 Hz, 1H), 7.91 (d, *J* = 8.0 Hz, 1H), 7.76 (d, *J* = 8.1 Hz, 1H), 7.67 (s, 1H), 7.62 – 7.50 (m, 2H), 7.30 (dd, *J* = 8.4, 7.1 Hz, 1H), 7.13 (d, *J* = 7.0 Hz, 1H), 5.51 (dd, *J* = 11.1, 4.5 Hz, 1H), 4.15 (dd, *J* = 14.5, 4.6 Hz, 1H), 3.89 – 3.83 (m, 2H), 3.73 (dd, *J* = 14.5, 11.2 Hz, 1H), 3.16 (qd, *J* = 7.3, 1.2 Hz, 15H), 2.87 (q, *J* = 6.1 Hz, 2H), 2.25 (dd, *J* = 4.6, 1.2 Hz, 3H), 1.24 (td, *J* = 7.3, 1.2 Hz, 20H). ^{31}P NMR (202 MHz, D_2O) δ -26.45. HRMS (ESI-TOF) m/z calculated for $C_{19}H_{18}N_3O_6P$ $[M-H]^-$ 415.0944; 416.1011. Brown oil, 23% yield.

2-(4-(2-((acetyloxidophosphoryl)oxy)ethyl)-1*H*-1,2,3-triazol-1-yl)-2-benzyl-3-phenylpropanoate 2(triethylammonium) (**8**). ^{1}H NMR (500 MHz, D_2O) δ 7.46 (s, 1H), 7.08 (dd, J = 5.0, 1.9 Hz, 6H), 6.83 (dd, J = 6.8, 2.9 Hz, 4H), 3.81 (q, J = 6.8 Hz, 2H), 3.58 – 3.21 (m, 4H), 3.04 (q, J = 7.3 Hz, 13H), 2.78 (t, J = 6.8 Hz, 2H), 2.20 (d, J = 4.6 Hz, 2H), 1.12 (t, J = 7.3 Hz, 18H). ^{31}P NMR (202 MHz, D_2O) δ -26.47. HRMS (ESI-TOF) m/z calculated for $C_{22}H_{22}N_3O_6P$ $[M-H]^-$ 455.1257; found 456.1326. Light brown oil, 15% yield.

2-(1-(1,3-diphenylpropan-2-yl)-1*H*-1,2,3-triazol-4-yl)ethyl acetylphosphonate triethylammonium (**10**). ^{1}H NMR (500 MHz, D_2O) δ 7.69 (d, *J* = 5.3 Hz, 1H), 7.25 (d, *J* = 11.3 Hz, 5H), 7.08 (d, *J* = 6.9 Hz, 4H), 5.01 (s, 1H), 3.95 (d, *J* = 7.7 Hz, 2H), 3.41 (d, *J* = 13.9 Hz, 2H), 3.27 (s, 2H), 3.22 – 3.15 (m, 5H), 2.86 (d, *J* = 6.2 Hz, 2H), 2.28 (d, *J* = 6.2 Hz, 2H), 1.27 (h, *J* =

5.6 Hz, 8H). ^{31}P NMR (202 MHz, D_2O) δ -26.61. HRMS (ESI-TOF) m/z calculated for $C_{21}H_{23}N_3O_4P$ [M-H]$^-$ 412.1432; found 412.1421. Light yellow oil, 30% yield.

2-(1-(2-benzyl-1-methoxy-1-oxo-3-phenylpropan-2-yl)-1*H*-1,2,3-triazol-4-yl)ethyl acetylphosphonate (**11**). ^{1}H NMR (500 MHz, MeOD) δ 7.73 (s, 1H), 7.20 (dd, J = 5.0, 1.9 Hz, 6H), 6.90 – 6.84 (m, 5H), 4.05 (q, J = 6.9 Hz, 2H), 3.82 – 3.65 (m, 8H), 3.33 (p, J = 1.6 Hz, 22H), 3.00 (t, J = 6.9 Hz, 2H), 2.32 (d, J = 4.1 Hz, 3H). ^{31}P NMR (202 MHz, MeOD) δ -26.27. HRMS (ESI-TOF) m/z calculated for $C_{23}H_{25}N_3O_6P$ [M-H]$^-$ 470.1486; found 471.1506. White solid, 35% yield.

2-(4-(2-((acetyloxidophosphoryl)oxy)ethyl)-1*H*-1,2,3-triazol-1-yl)-2,3-dihydro-1*H*-indene-2-carboxylate 4(triethylammonium) acetate (**12**). ^{1}H NMR (500 MHz, METHANOL-*D_4*) δ 7.98 (d, *J* = 2.9 Hz, 1H), 7.19 (dt, *J* = 7.2, 3.3 Hz, 2H), 7.10 (dd, *J* = 5.6, 3.1 Hz, 2H), 4.13 – 4.04 (m, 2H), 3.99 – 3.80 (m, 5H), 3.11 (qd, *J* = 7.4, 2.7 Hz, 22H), 3.02 – 2.95 (m, 2H), 2.24 (d, *J* = 3.9 Hz, 3H), 1.89 (d, *J* = 3.1 Hz, 3H), 1.24 (td, *J* = 7.4, 2.8 Hz, 34H). HRMS (ESI-TOF) m/z calculated for $C_{16}H_{16}N_3O_6P$ [M-H]$^-$ 377.0788; found 378.0855. Brown oil, 25% yield.

1-(4-(2-((acetyloxidophosphoryl)oxy)ethyl)-1*H*-1,2,3-triazol-1-yl)cyclopentane-1-carboxylate 3(triethylammonium) (**13**). ^{1}H NMR (500 MHz, D_2O) δ 7.95 (s, 1H), 4.15 (q, J = 6.4 Hz, 2H), 3.21 (q, J = 7.3 Hz, 24H), 3.07 (t, J = 6.4 Hz, 3H), 2.61 – 2.38 (m, 4H), 2.31 (d, J = 4.6 Hz, 3H), 1.91 (d, J = 20.9 Hz, 4H), 1.61 (d, J = 6.0 Hz, 4H), 1.29 (t, J = 7.3 Hz, 35H). ^{31}P NMR (202 MHz, D_2O) δ -27.23. HRMS (ESI-TOF) m/z calculated for $C_{12}H_{16}N_3O_6P$ [M-H]$^-$ 329.0788; found 330.0854. Dark brown oil, 14% yield.

(*R*)-2-(4-(2-((acetyloxidophosphoryl)oxy)ethyl)-1*H*-1,2,3-triazol-1-yl)-3-cyclohexylpropanoate 2(triethylammonium) (**14**). ^{1}H NMR (500 MHz, D_2O) δ 7.96 (s, 1H), 5.23 (dd, J = 11.3, 4.5 Hz, 1H), 4.17 (q, J = 6.4 Hz, 2H), 3.21 (q, J = 7.3 Hz, 13H), 3.08 (t, J = 6.3 Hz, 2H), 2.33 (d, J = 4.6 Hz, 3H), 2.20 – 2.00 (m, 2H), 1.85 –1.48 (m, 7H), 1.29 (t, J = 7.3 Hz, 19H), 1.21 – 0.87 (m, 8H). ^{31}P NMR (202 MHz, MeOD) δ -34.80. HRMS (ESI-TOF) m/z calculated for $C_{15}H_{22}N_3O_6P$ [M-H]$^-$ 371.1257; found 372.1332. Light yellow oil, 18% yield.

(*R*)-2-(4-(2-((acetyloxidophosphoryl)oxy)ethyl)-1*H*-1,2,3-triazol-1-yl)-2-cyclohexylacetate 2(triethylammonium) (**15**). ^{1}H NMR (500 MHz, D_2O) δ 8.00 (s, 1H), 4.86 (d, J = 8.9 Hz, 2H), 4.16 (q, J = 6.3 Hz, 2H), 3.21 (q, J = 7.3 Hz, 17H), 3.08 (t, J = 6.3 Hz, 2H), 2.31 (d, J = 4.6 Hz, 3H), 2.17 (d, J = 10.1 Hz, 1H), 1.82 – 1.57 (m, 6H), 1.28 (t, J = 7.3 Hz, 25H), 1.17 (dt, J = 26.6, 12.4 Hz, 2H), 1.04 – 0.90 (m, 0H). ^{31}P NMR (202 MHz, D_2O) δ -26.53. HRMS (ESI-TOF) m/z calculated for $C_{14}H_{20}N_3O_6P$ [M-H]$^-$ 357.1101; found 358.1164. Light yellow oil, 27% yield.

(2*R*)-2-(4-(2-((acetyloxidophosphoryl)oxy)ethyl)-1*H*-1,2,3-triazol-1-yl)-2-(adamantan-2-yl)acetate 2(triethylammonium) (**16**). ^{1}H NMR (500 MHz, D_2O) δ8.21 (s, 1H), 4.81 (s, 1H), 4.21 – 4.13 (m, 1H), 3.21 (q, J = 7.4 Hz, 13H), 3.08 (t, J = 6.3 Hz, 2H), 2.30 (d, J = 4.6 Hz, 2H), 1.97 (s, 3H), 1.71 (t, J = 12.6 Hz, 6H), 1.59 (d, J = 12.3 Hz, 3H), 1.43 (d, J = 11.7 Hz, 3H), 1.28 (t, J = 7.4 Hz, 18H). ^{31}P NMR (202 MHz, D_2O) δ -26.85. HRMS (ESI-TOF) m/z calculated for $C_{18}H_{24}N_3O_6P$ [M-H]$^-$ 409.1414; found 410.1476. Light yellow oil, 9% yield.

(2*S*)-2-(4-(2-((acetyloxidophosphoryl)oxy)ethyl)-1*H*-1,2,3-triazol-1-yl)-2-(adamantan-2-yl)acetate 2(triethylammonium) (**17**). 1H NMR (500 MHz, D_2O) δ 8.21 (s, 1H), 4.81 (s, 2H), 4.17 (qd, J = 6.4, 2.5 Hz, 1H), 3.20 (q, J = 7.3 Hz, 13H), 3.08 (t, J = 6.3 Hz, 2H), 2.30 (d, J = 4.6 Hz, 2H), 1.97 (s, 3H), 1.70 (t, J = 12.5 Hz, 6H), 1.59 (d, J = 12.3 Hz, 3H), 1.43 (d, J = 12.0 Hz, 3H), 1.28 (t, J = 7.3 Hz, 19H). ^{31}P NMR (202 MHz, D_2O) δ -26.87. HRMS (ESI-TOF) m/z calculated for $C_{18}H_{24}N_3O_6P$ $[M-H]^-$ 409.1414; found 410.1477. Light yellow oil, 15% yield.

(*R*)-2-(1-(1-hydroxy-3-phenylpropan-2-yl)-1*H*-1,2,3-triazol-4-yl)ethyl acetylphosphonate 2(triethylammonium) acetate salt (**18**). ^1H NMR (500 MHz, METHANOL-D_4) δ 7.91 (s, 1H), 7.30 – 7.11 (m, 3H), 7.12 – 7.06 (m, 2H), 5.04 (ddd, *J* = 11.6, 9.4, 6.0 Hz, 1H), 4.51 – 4.41 (m, 2H), 4.07 (q, *J* = 6.5 Hz, 2H), 3.29 – 3.22 (m, 2H), 2.97 (t, *J* = 6.5 Hz, 2H), 2.31 (d, *J* = 4.1 Hz, 2H), 1.98 (s, 3H), 1.28 (t, *J* = 7.3 Hz, 10H). ^{31}P NMR (202 MHz, METHANOL-D_4) δ -25.79. HRMS (ESI-TOF) m/z calculated for $C_{15}H_{19}N_3O_5P$ $[M-H]^-$ 352.1068; found 352.1062. Light brown oil, 5% yield.

(*S*)-2-(1-(1-hydroxy-3-phenylpropan-2-yl)-1*H*-1,2,3-triazol-4-yl)ethyl acetylphosphonate triethylammonium (**19**). ^1H NMR (500 MHz, D_2O) δ 7.86 (s, 1H), 7.32 – 7.18 (m, 3H), 7.11 – 7.02 (m, 2H), 4.92 (dq, J = 11.4, 5.2 Hz, 1H), 4.12 – 4.04 (m, 4H), 3.29 (dd, J = 14.1, 4.7 Hz, 1H), 3.20 (q, J = 7.3 Hz, 6H), 3.15 – 3.06 (m, 1H), 2.99 (t, J = 6.3 Hz, 2H), 2.31 (d, J = 4.6 Hz, 2H), 1.28 (t, J = 7.3 Hz, 7H). ^{31}P NMR (202 MHz, D_2O) δ -26.93. HRMS (ESI-TOF) m/z calculated for $C_{15}H_{19}N_3O_5P$ $[M-H]^-$ 352.1068; found 353.1091. Light yellow oil, 27% yield.

2-(1-benzhydryl-1*H*-1,2,3-triazol-4-yl)ethyl acetylphosphonate triethylammonium (**20**). ^1H NMR (500 MHz, D_2O) δ 7.82 (s, 1H), 7.46 (d, J = 7.3 Hz, 5H), 7.25 (s, 5H), 7.20 (s, 1H), 4.12 (s, 2H), 3.19 (d, J = 8.6 Hz, 7H), 3.04 (s, 2H), 2.21 (s, 3H), 1.28 (dt, J = 8.7, 4.4 Hz, 10H). ^{31}P NMR (202 MHz, D_2O) δ -27.17. HRMS (ESI-TOF) m/z calculated for $C_{19}H_{19}N_3O_4P$ $[M-H]^-$ 384.1119; found 384.1113. Light yellow oil, 25% yield.

(*R*)-2-(1-(1,1,3-triphenylpropan-2-yl)-1*H*-1,2,3-triazol-4-yl)ethyl acetylphosphonate triethylammonium (**21**). ^1H NMR (500 MHz, D_2O) δ 7.75 (d, J = 8.0 Hz, 3H), 7.52 (t, J = 7.6 Hz, 2H), 7.43 – 7.31 (m, 3H), 7.24 – 7.16 (m, 6H), 7.11 (t, J = 7.4 Hz, 1H), 6.94 (dd, J = 7.5, 2.1 Hz, 2H), 5.69 (td, J = 11.5, 3.6 Hz, 1H), 3.88 (q, J = 6.5 Hz, 2H), 3.21 (q, J = 7.3 Hz, 9H), 2.79 (t, J = 6.4 Hz, 2H), 2.29 (d, J = 4.6 Hz, 2H), 1.28 (t, J = 7.3 Hz, 10H). ^{31}P NMR (202 MHz, D_2O) δ -27.11. HRMS (ESI-TOF) m/z calculated for $C_{27}H_{27}N_3O_4P$ $[M-H]^-$ 488.1745; found 488.1743. Light yellow oil, 37% yield.

(*S*)-2-(1-(1,1,3-triphenylpropan-2-yl)-1*H*-1,2,3-triazol-4-yl)ethyl acetylphosphonate triethylammonium (**22**). ^1H NMR (500 MHz, D_2O) δ 7.72 (d, J = 6.9 Hz, 3H), 7.50 (t, J = 7.6 Hz, 2H), 7.36 (dd, J = 25.1, 7.5 Hz, 3H), 7.25 – 7.14 (m, 5H), 7.10 (t, J = 7.4 Hz, 1H), 6.92 (dd, J = 7.1, 2.5 Hz, 2H), 5.66 (td, J = 11.4, 3.6 Hz, 1H), 4.74 (d, J = 11.8 Hz, 1H), 3.87 (q, J = 6.5 Hz, 2H), 3.18 (q, J = 7.3 Hz, 8H), 2.77 (t, J = 6.4 Hz, 2H), 2.28 (d, J = 4.6 Hz, 2H), 1.27 (t, J = 7.3 Hz, 9H). ^{31}P NMR (202 MHz, D_2O) δ -27.32. HRMS (ESI-TOF) m/z calculated for $C_{27}H_{27}N_3O_4P$ $[M-H]^-$ 488.1745; found 488.1739. Light yellow oil, 36% yield.

^{31}P and ^{1}H NMR, HPLC, and HRMS for bisubstrate analog inhibitors.

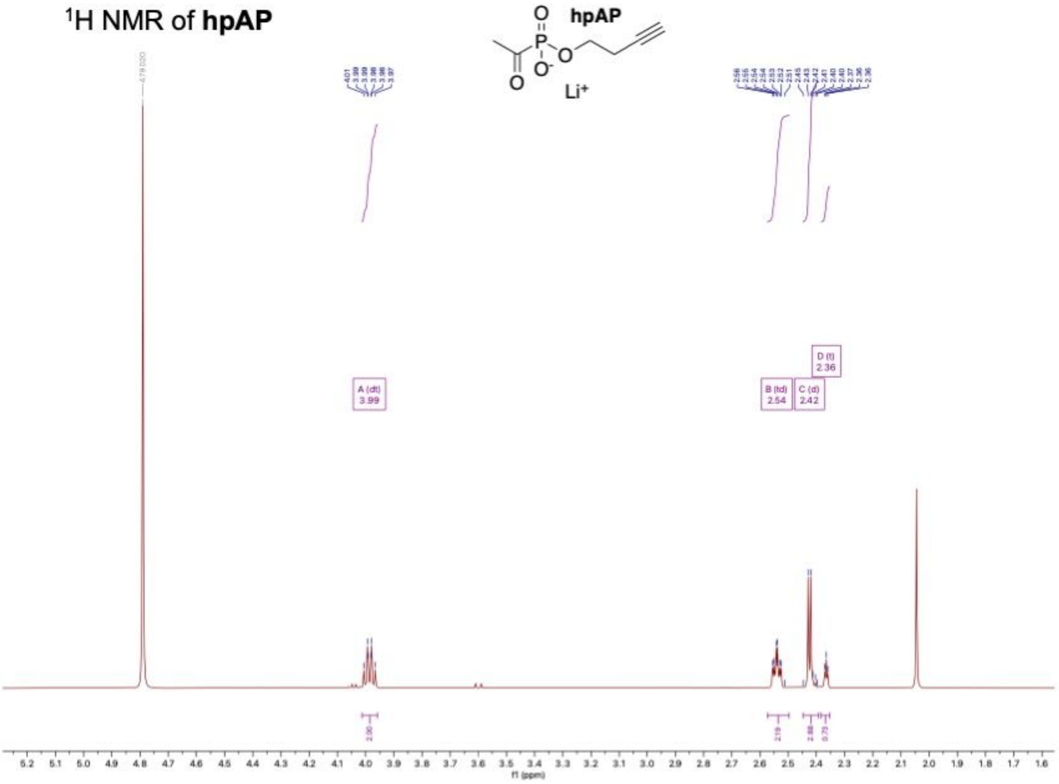

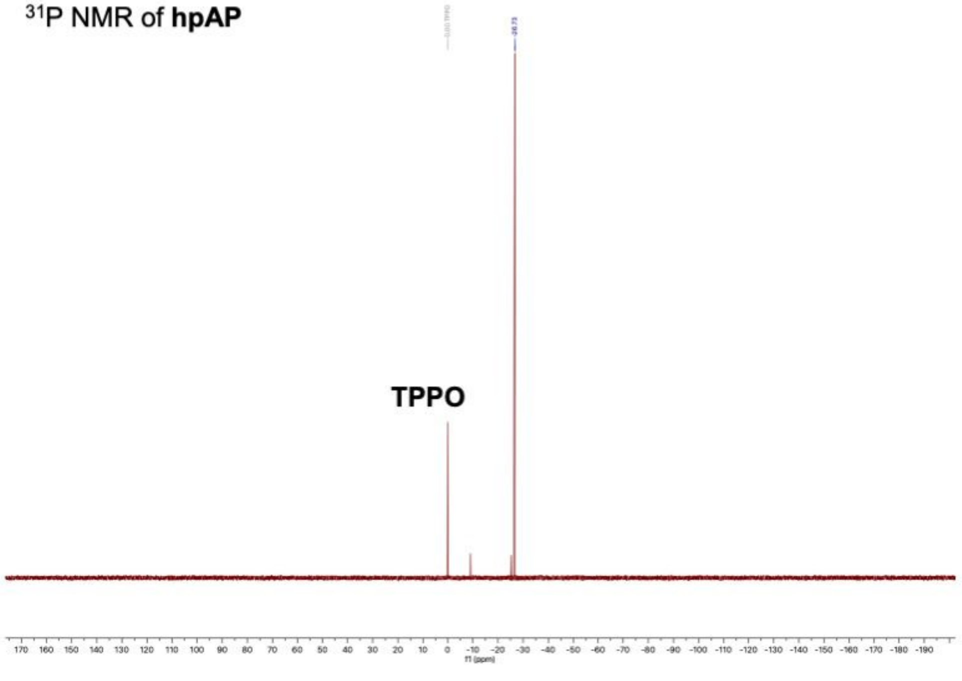

RP-HPLC (335 nm) of **hpAP**

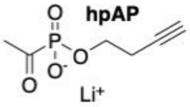

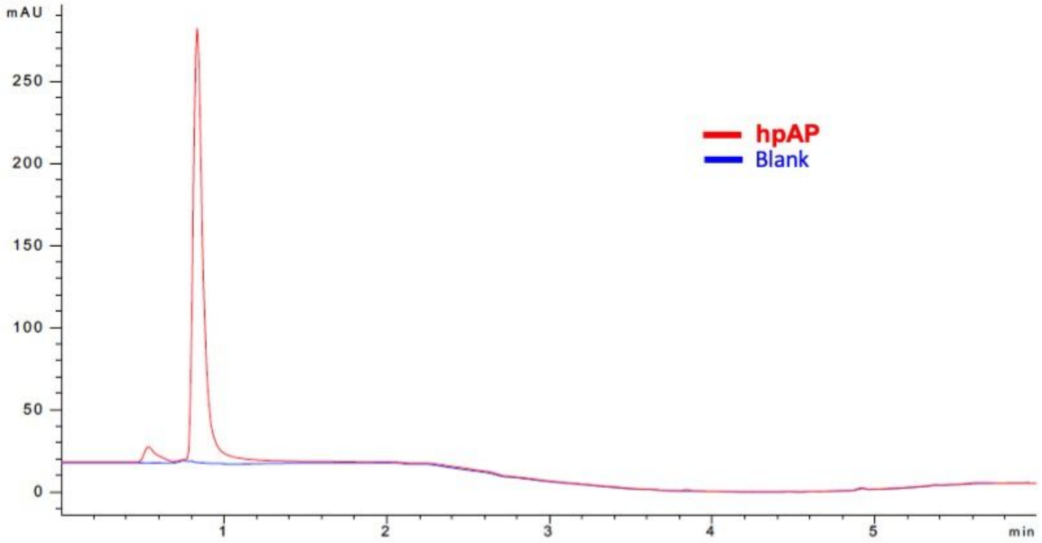

HRMS of **hpAP**

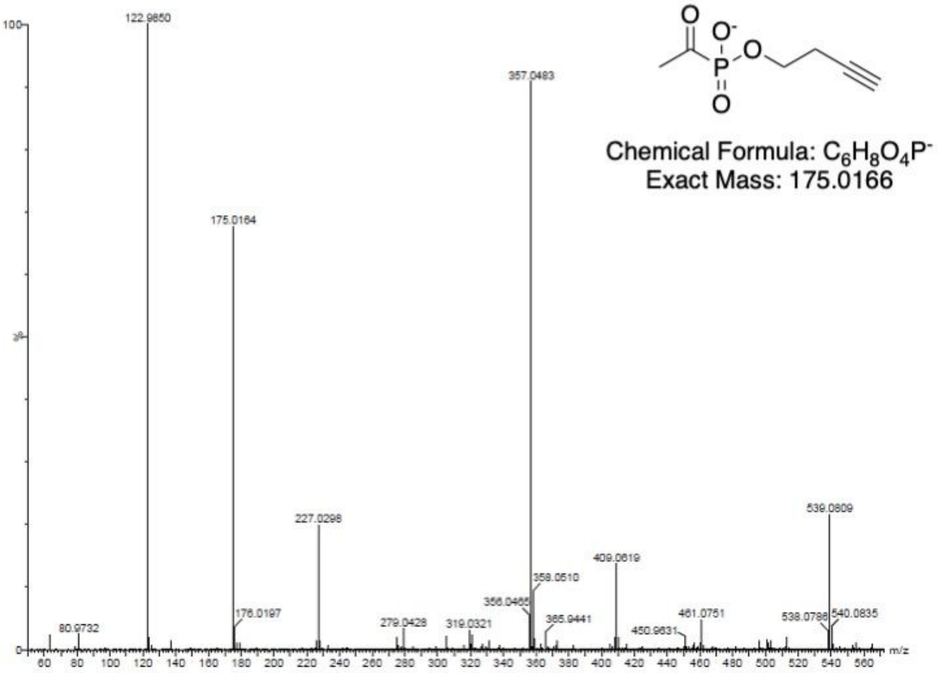

¹H NMR of **2**

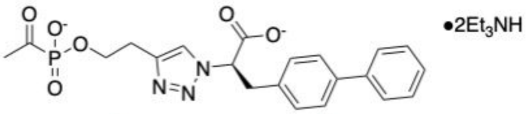

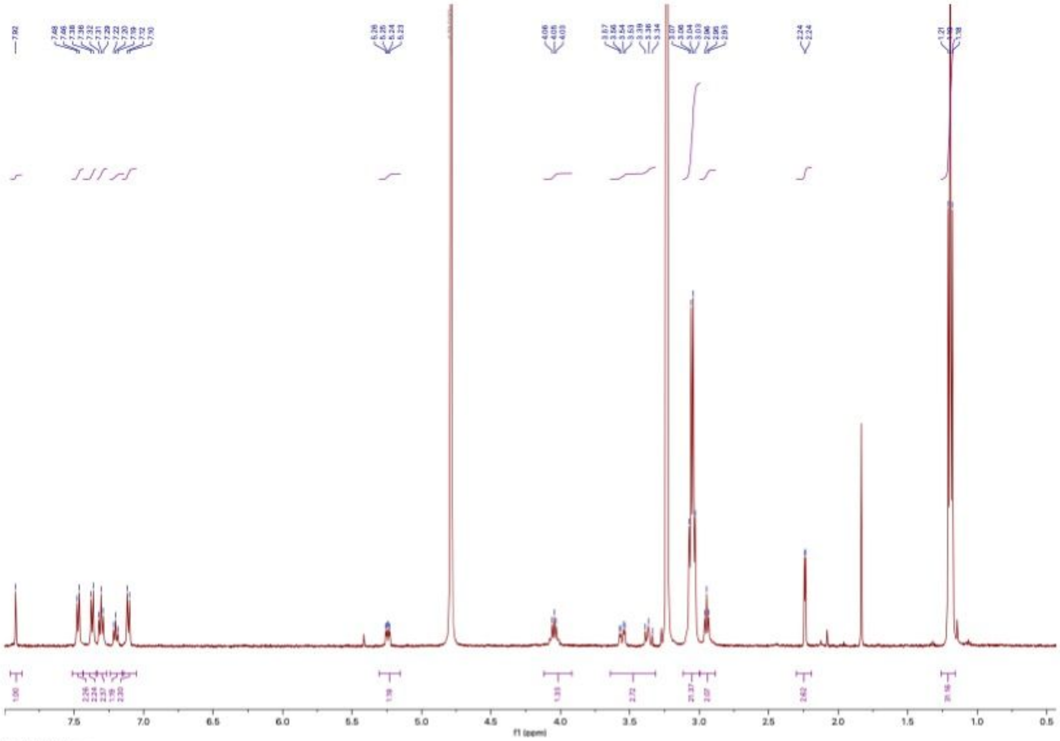

leb-111-12-31p-tppo

³¹P NMR of **2**

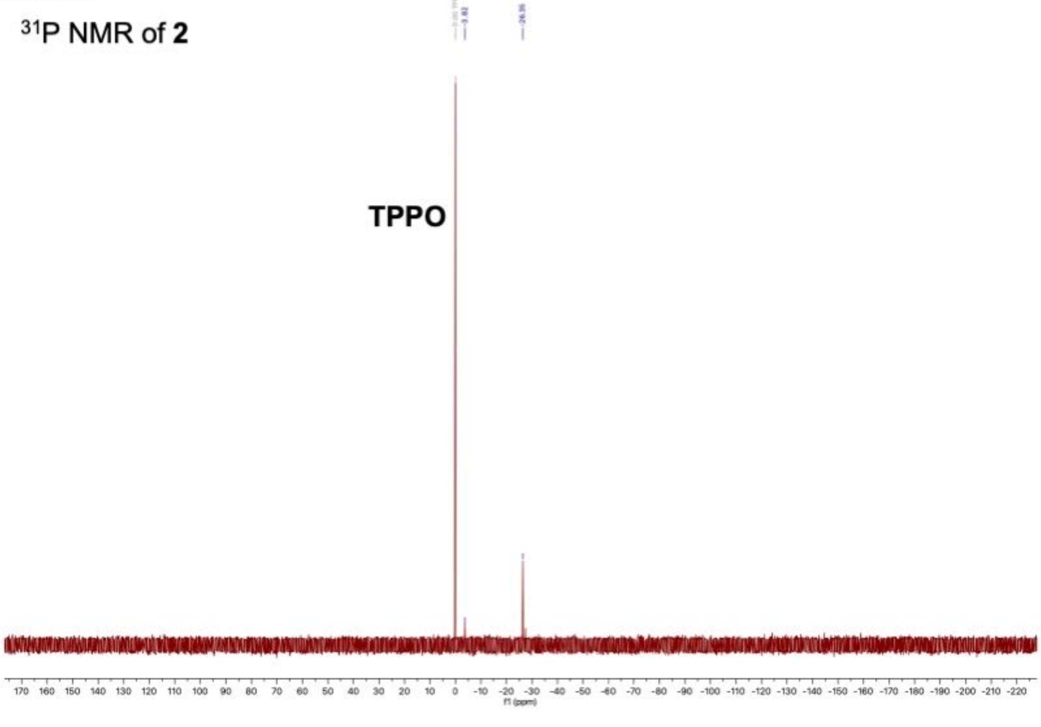

RP-HPLC of **2**

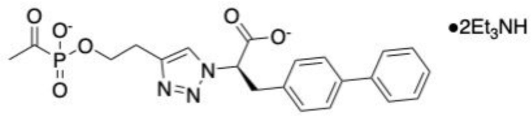

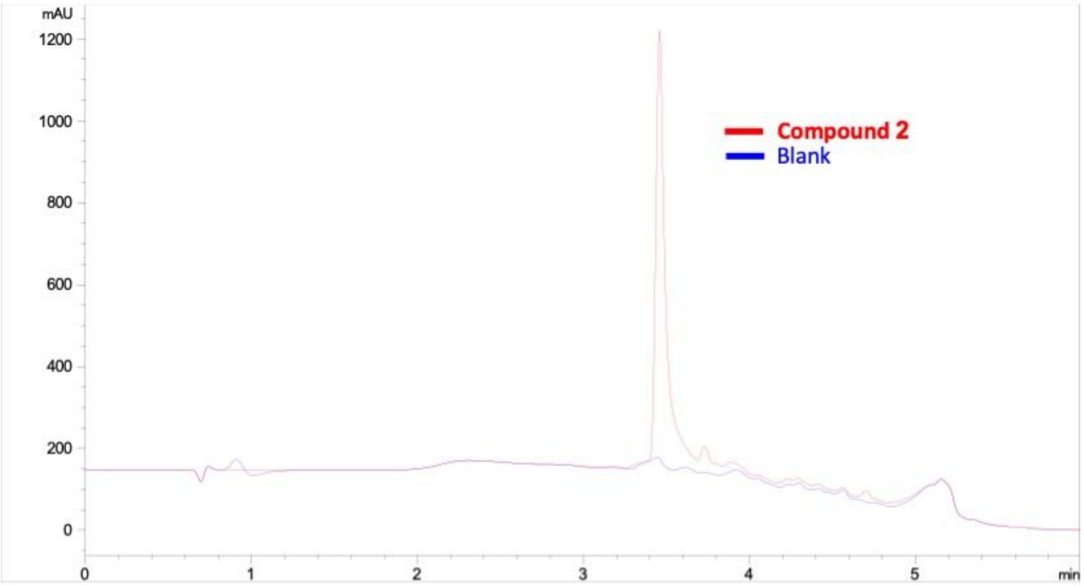

HRMS of **2**

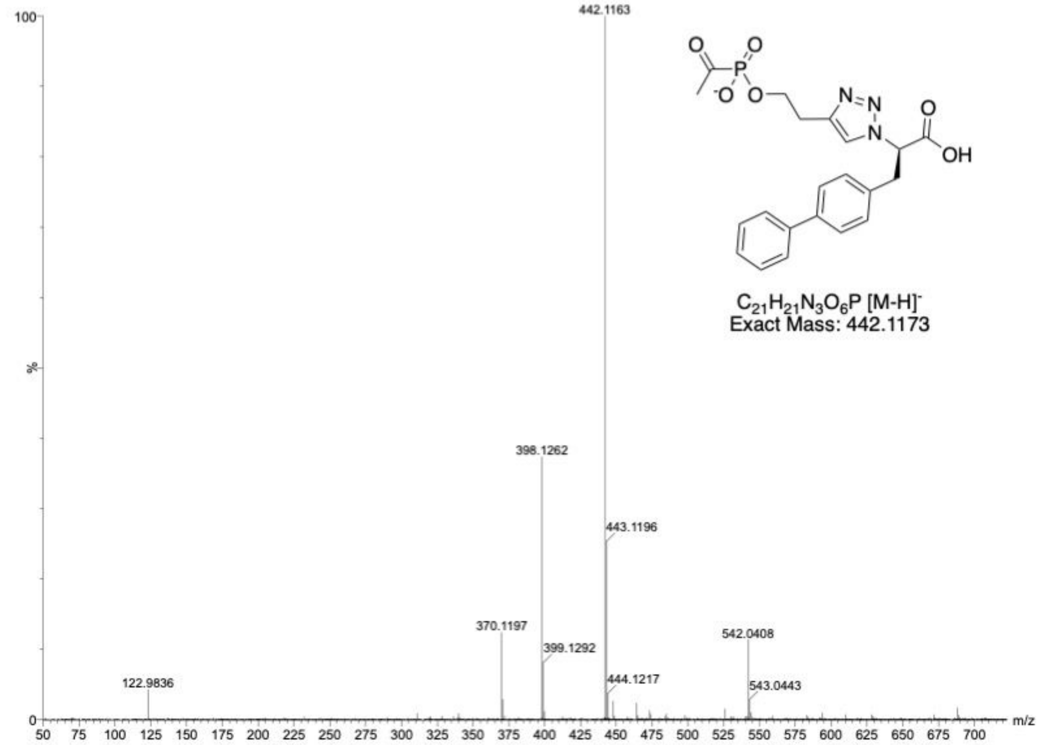

^{1}H NMR of **3**

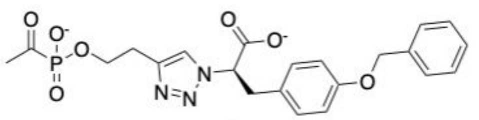

•2Et_3NH

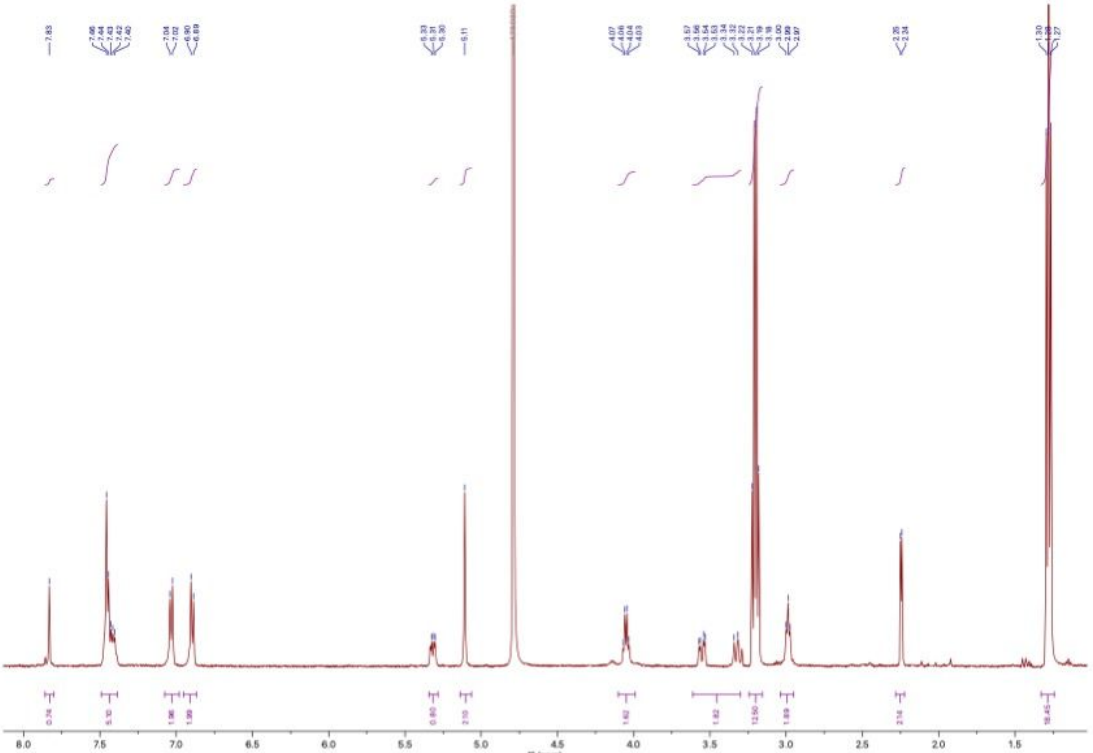

^{31}P NMR of **3**

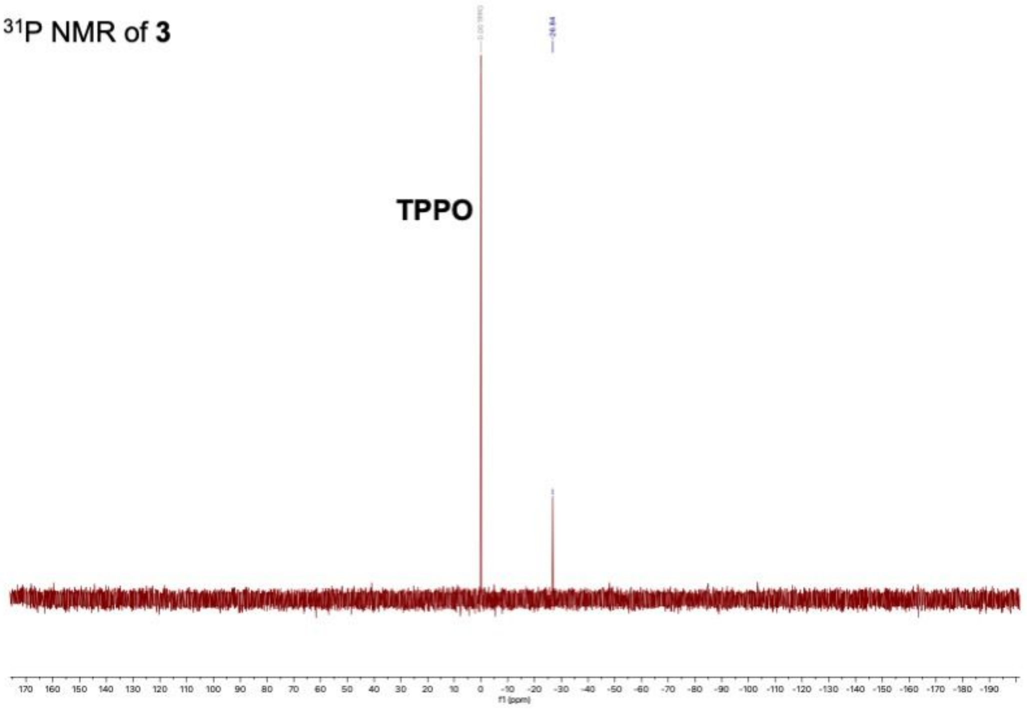

RP-HPLC of **3**

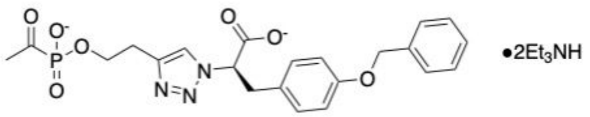

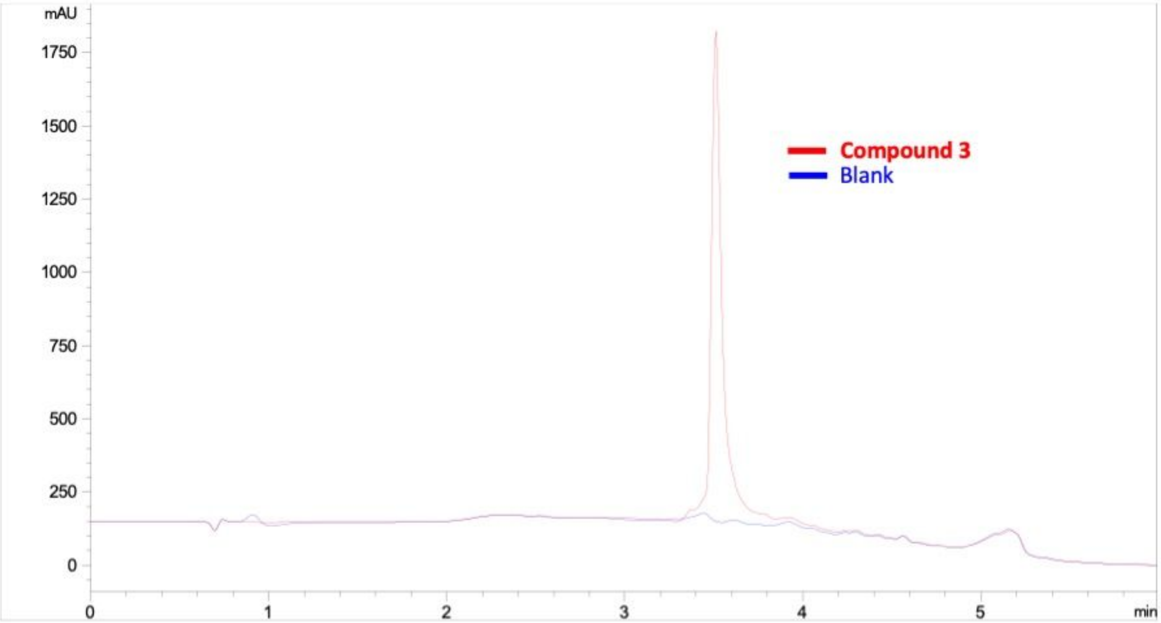

HRMS of **3**

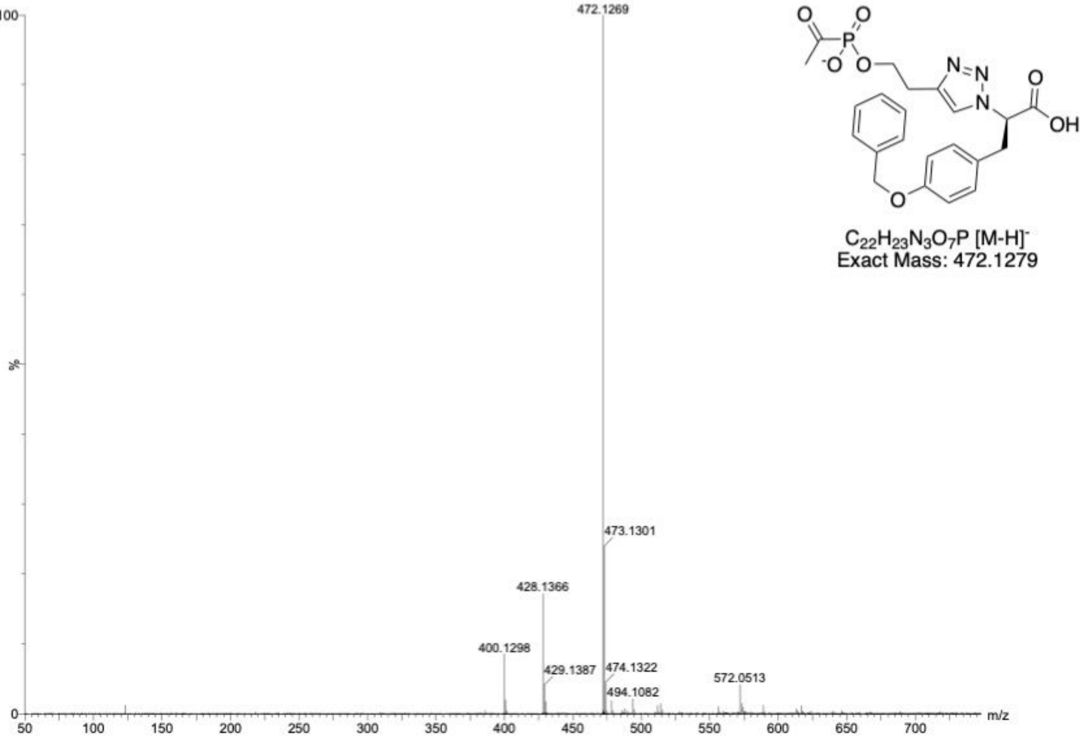

$C_{22}H_{23}N_3O_7P$ [M-H]$^-$
Exact Mass: 472.1279

^{1}H NMR of **4**

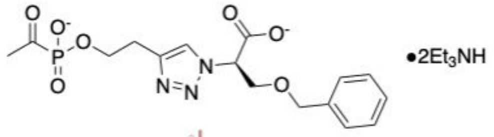

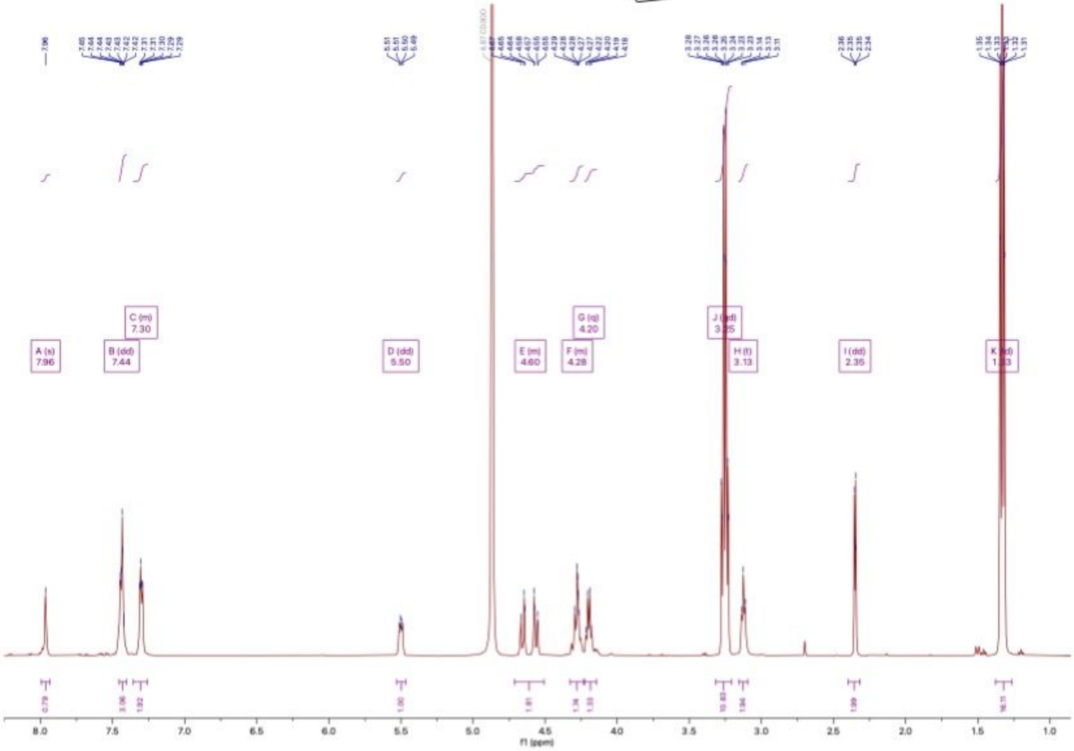

^{31}P NMR of **4**

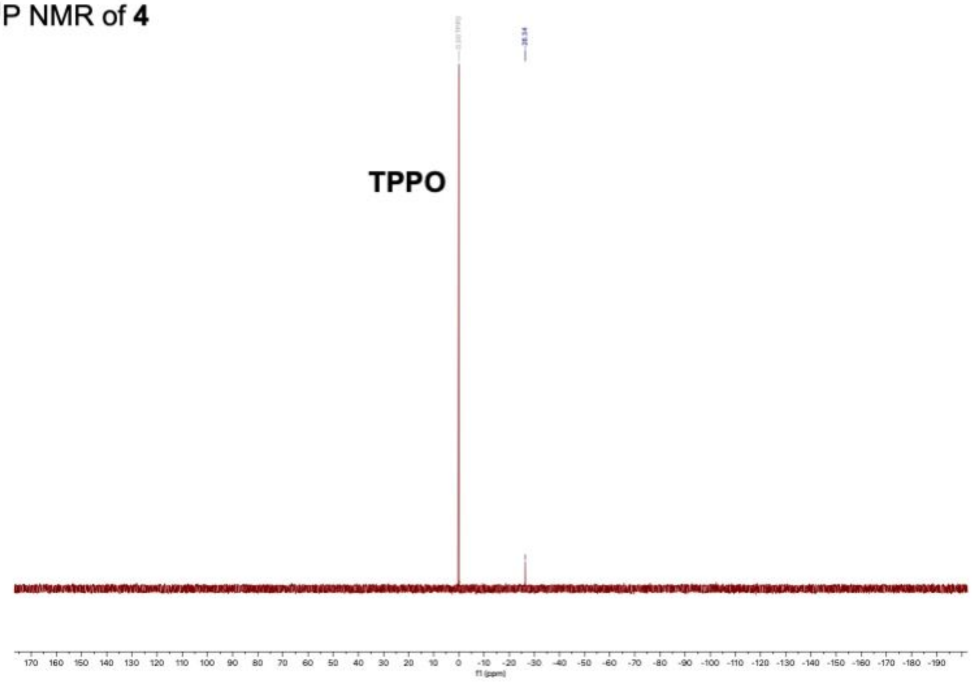

RP-HPLC of **4**

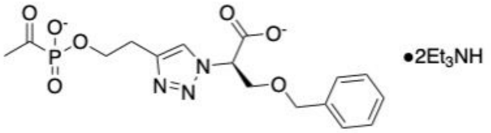

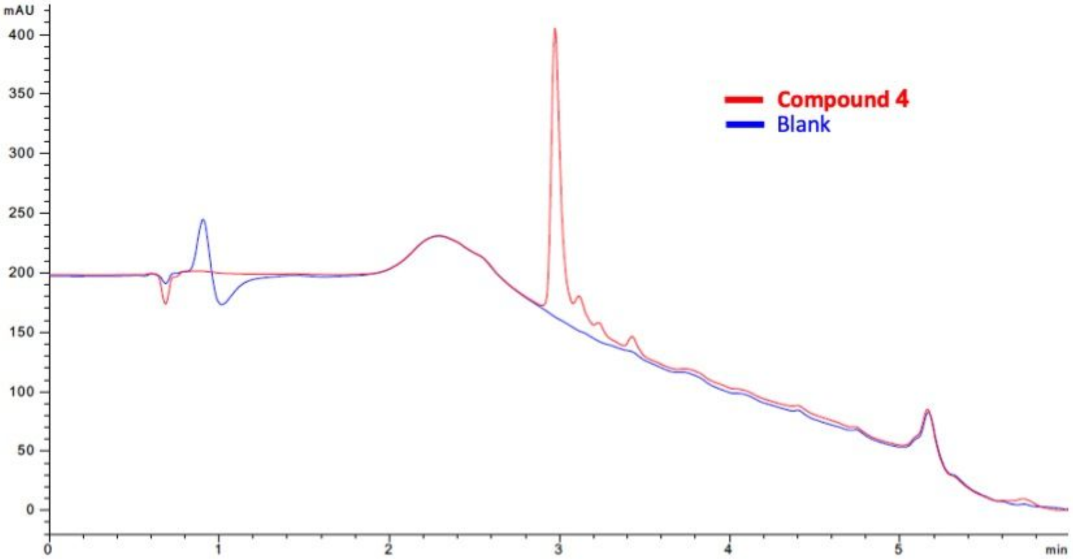

HRMS of **4**

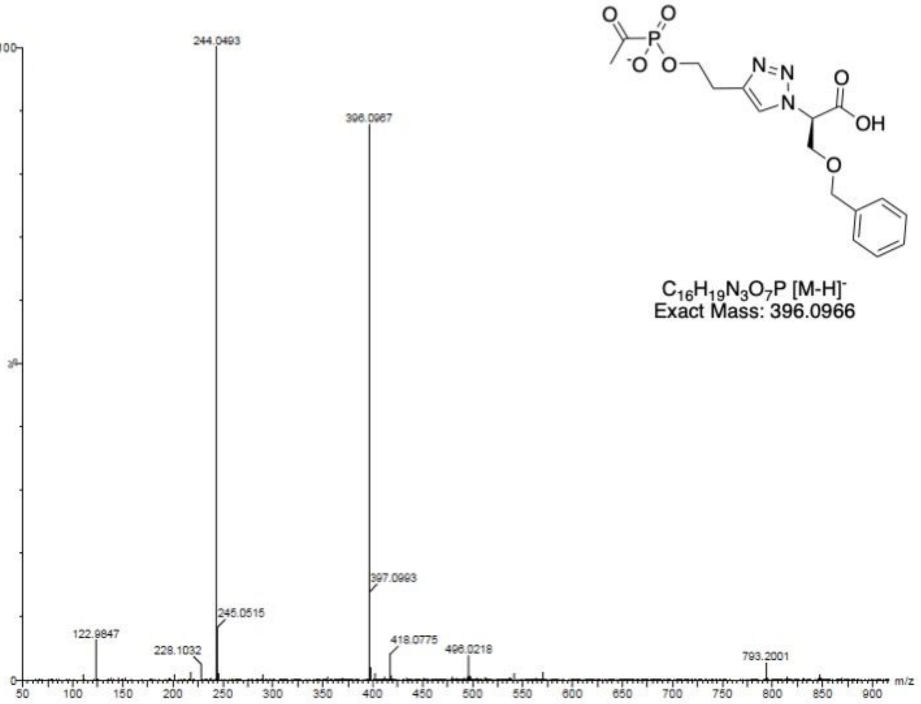

^{1}H NMR of **5**

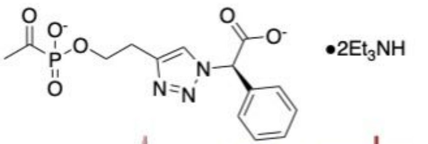

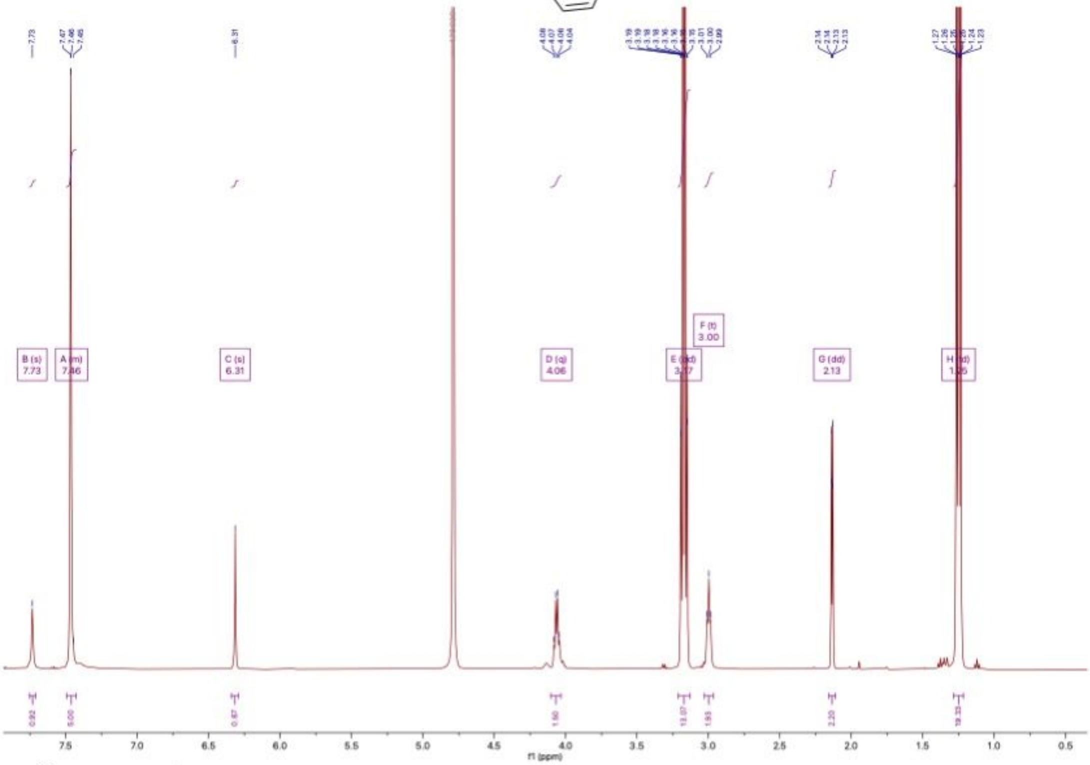

^{31}P NMR of **5**

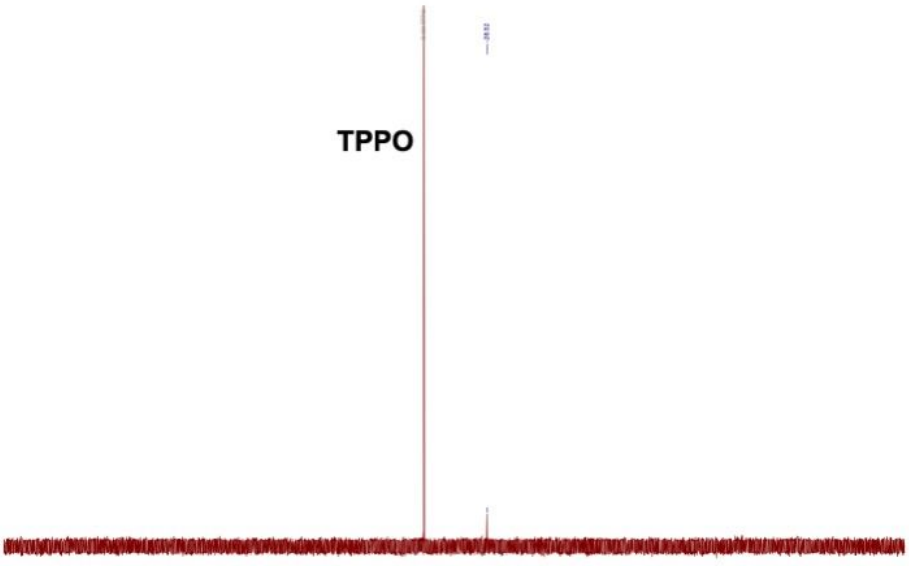

RP-HPLC of **5**

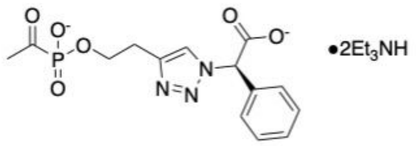

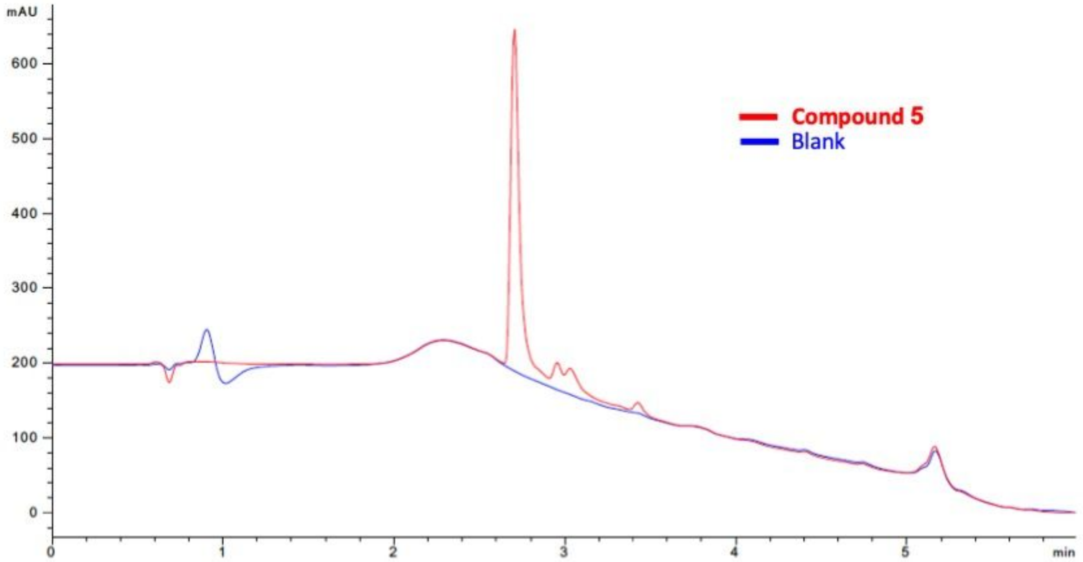

HRMS of **5**

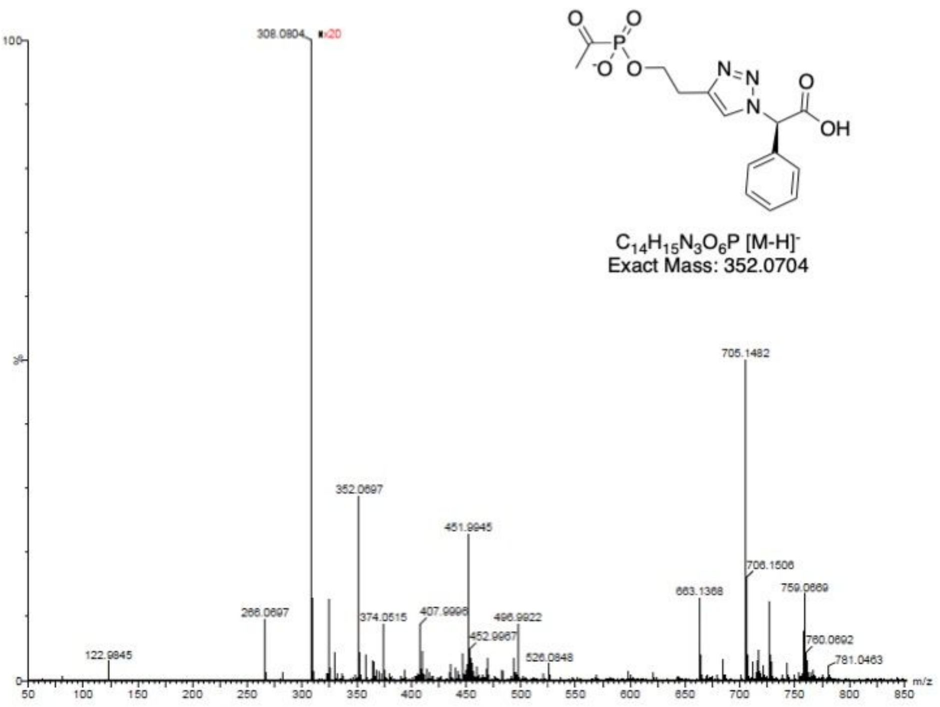

^{1}H NMR of **6**

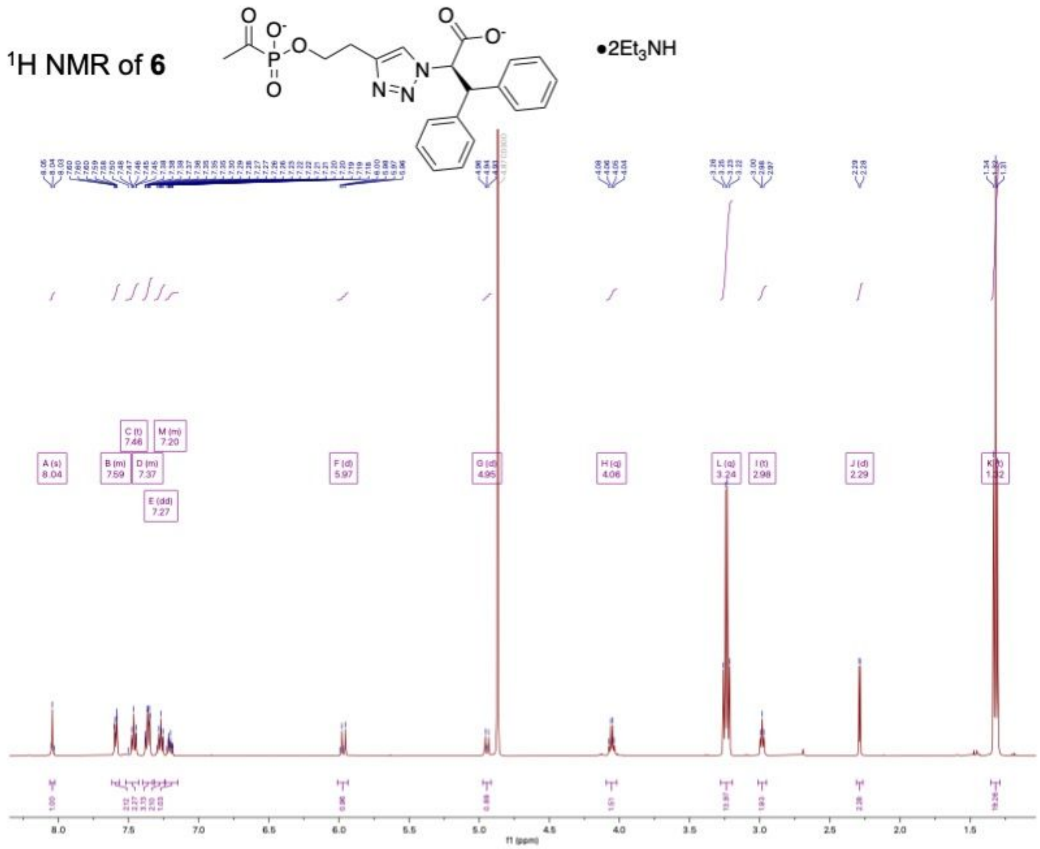

^{31}P NMR of **6**

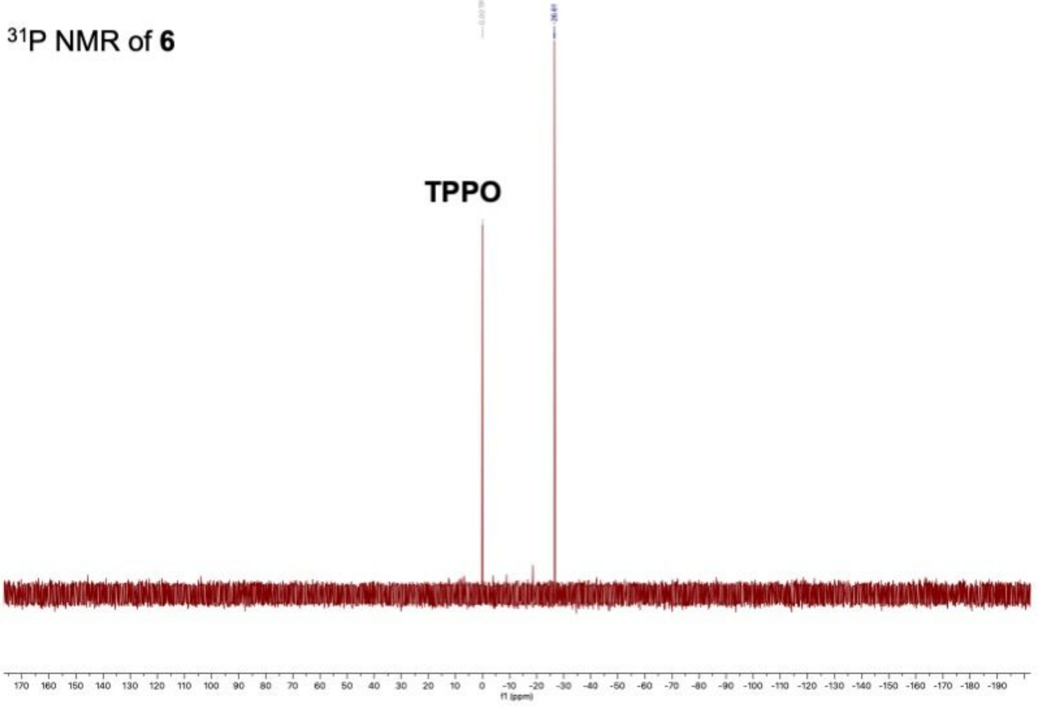

RP-HPLC of **6**

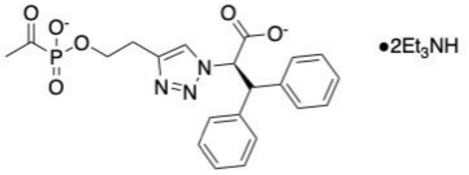

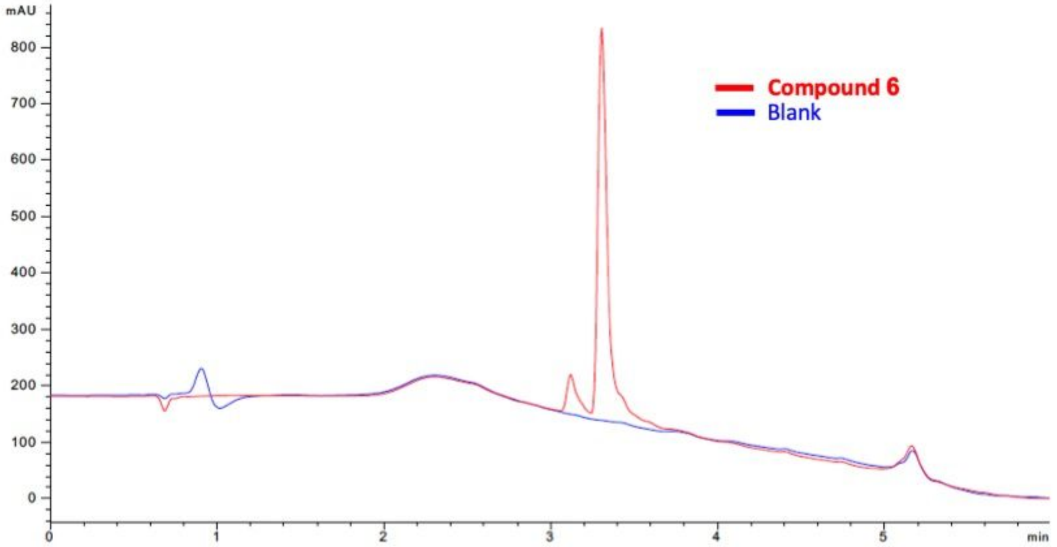

HRMS of **6**

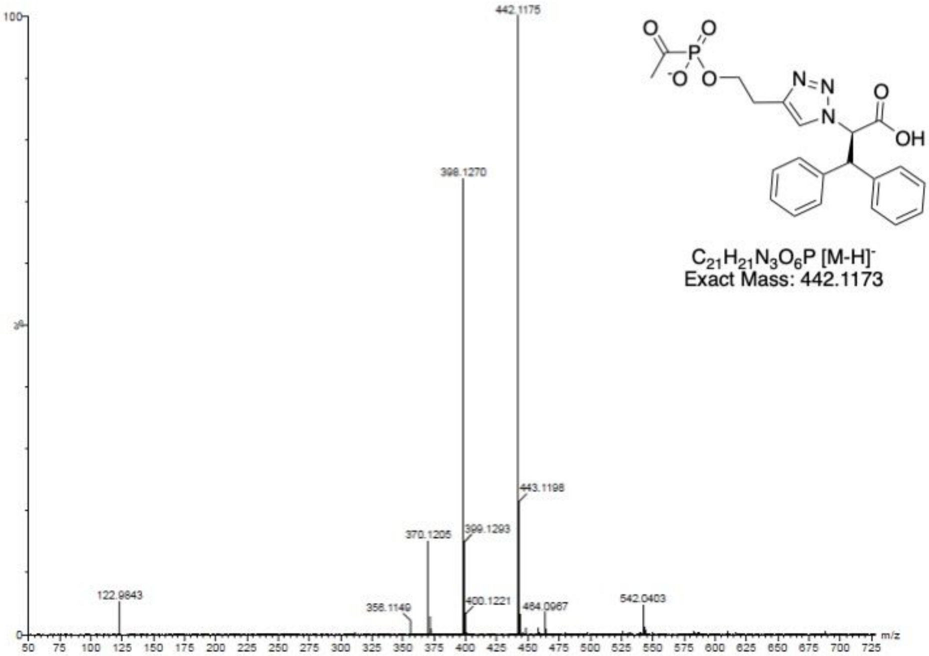

^{1}H NMR of **7**

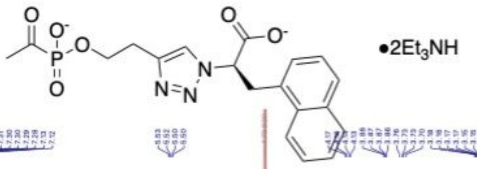

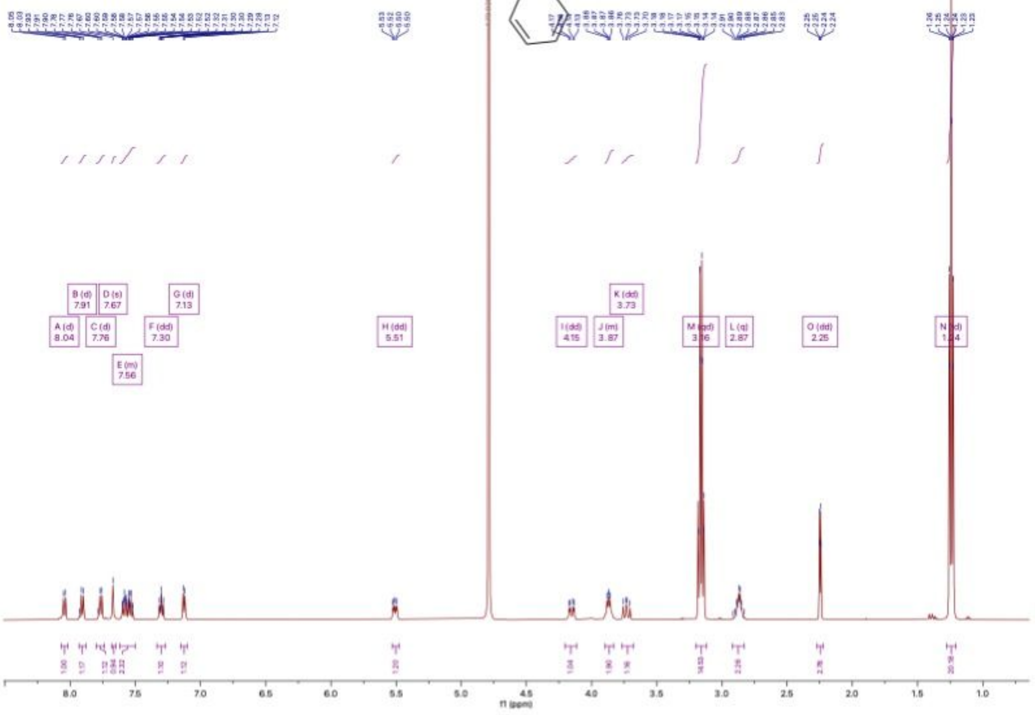

^{31}P NMR of **7**

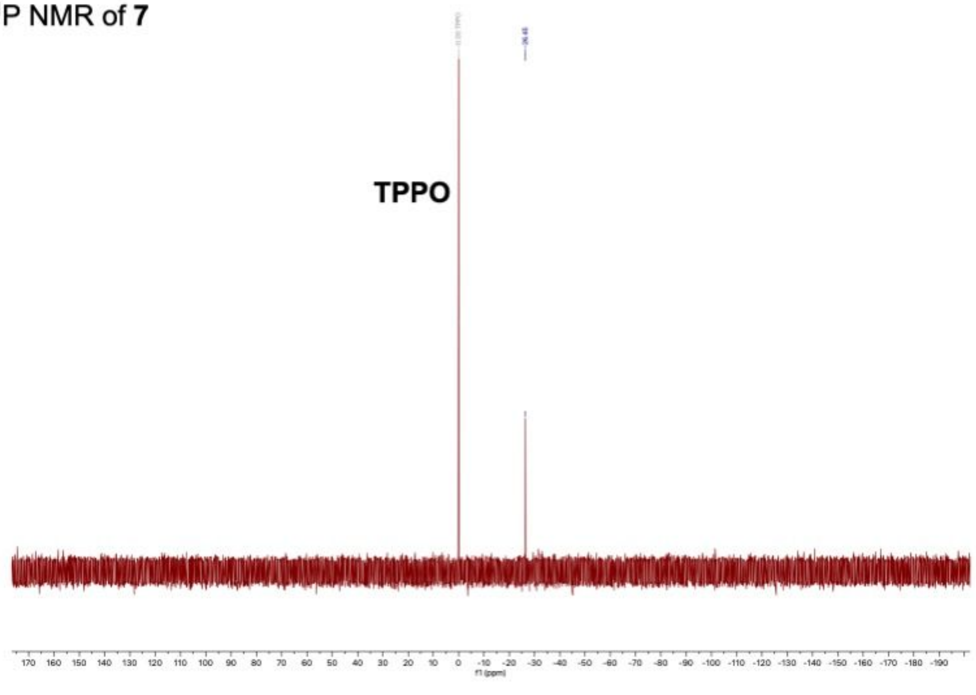

RP-HPLC of **7**

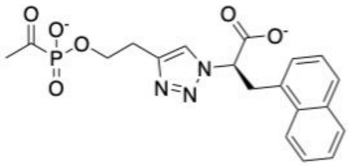

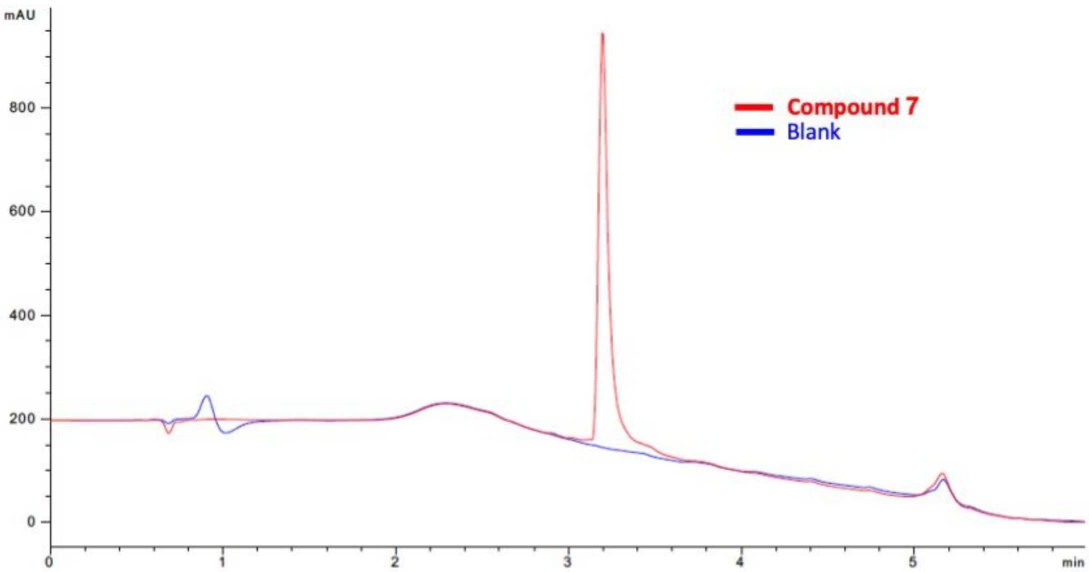

HRMS of **7**

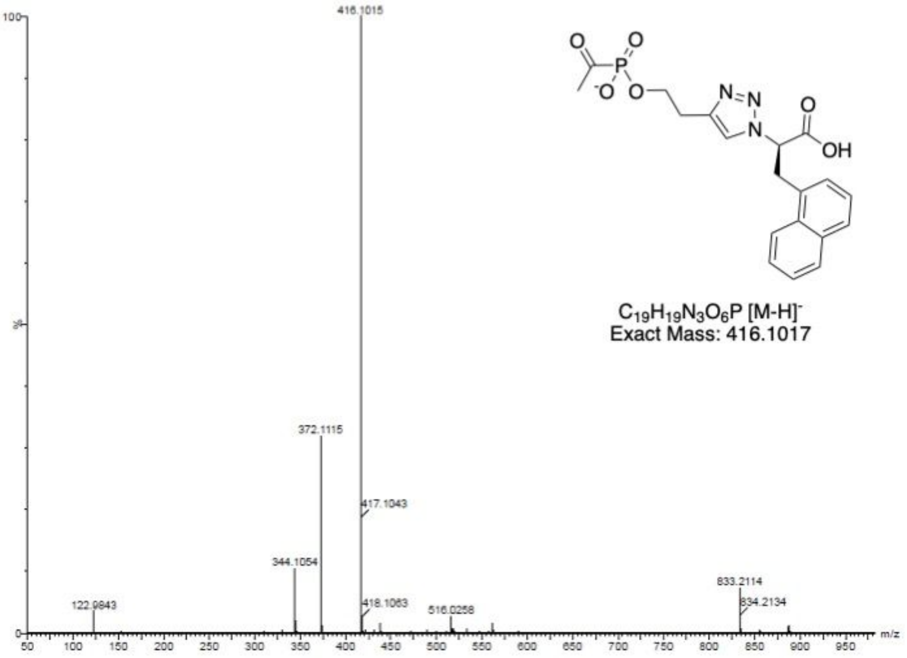

^{1}H NMR of **8**

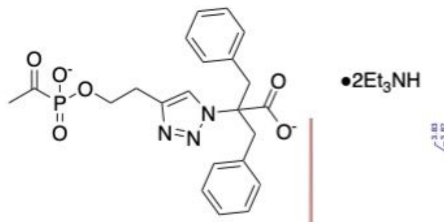

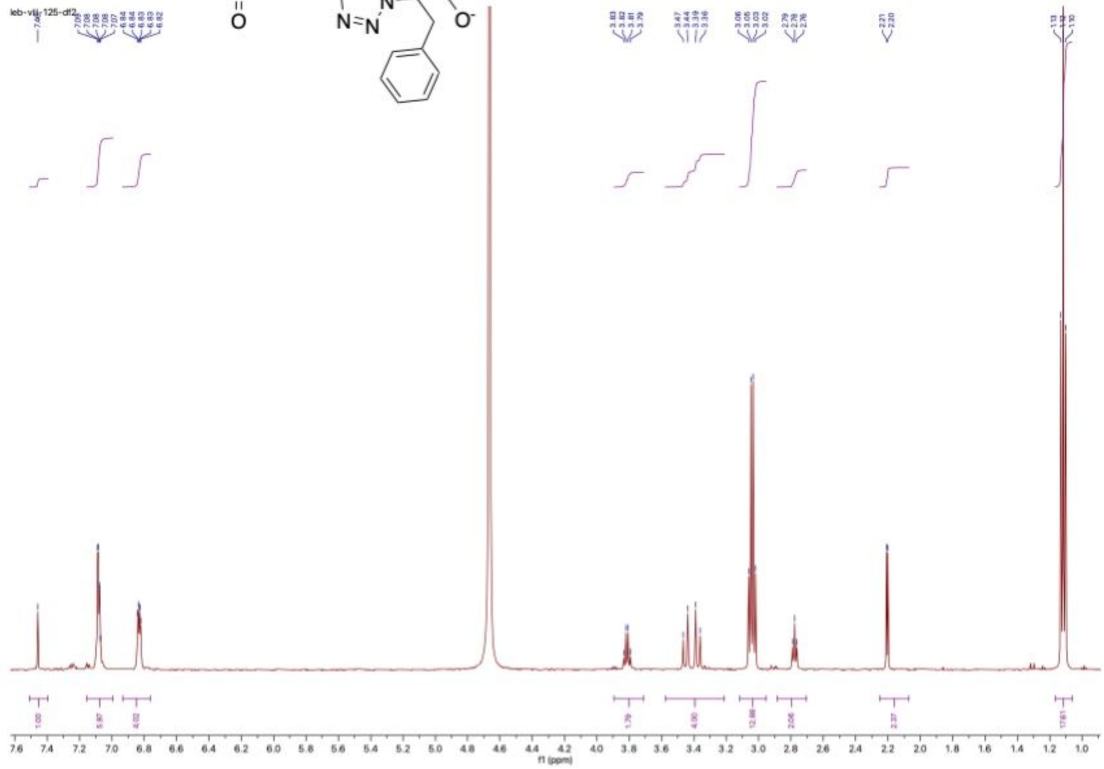

^{31}P NMR of **8**

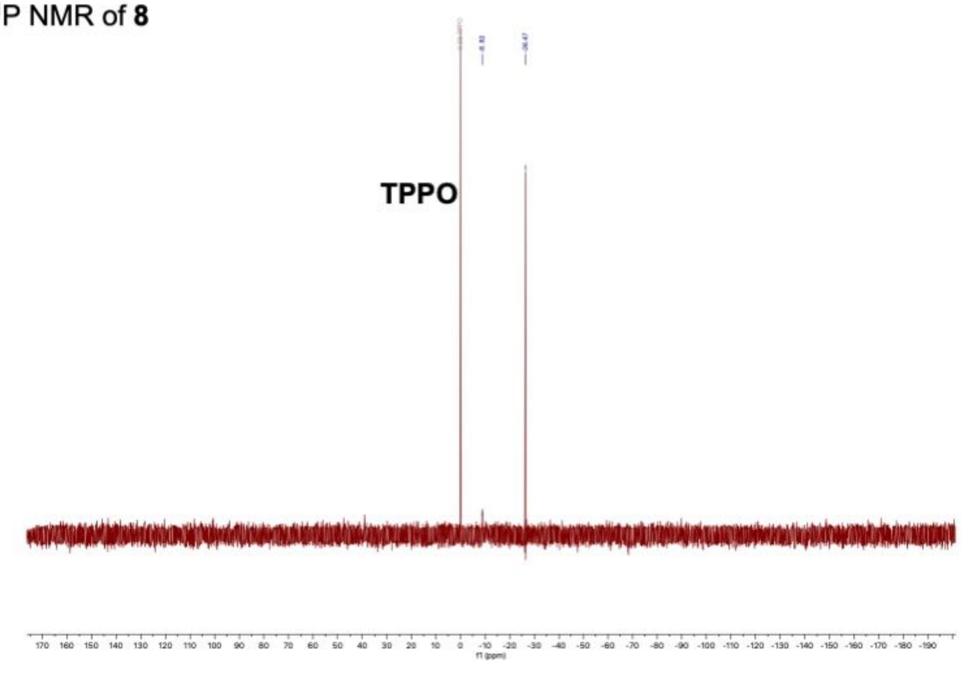

RP-HPLC of **8**

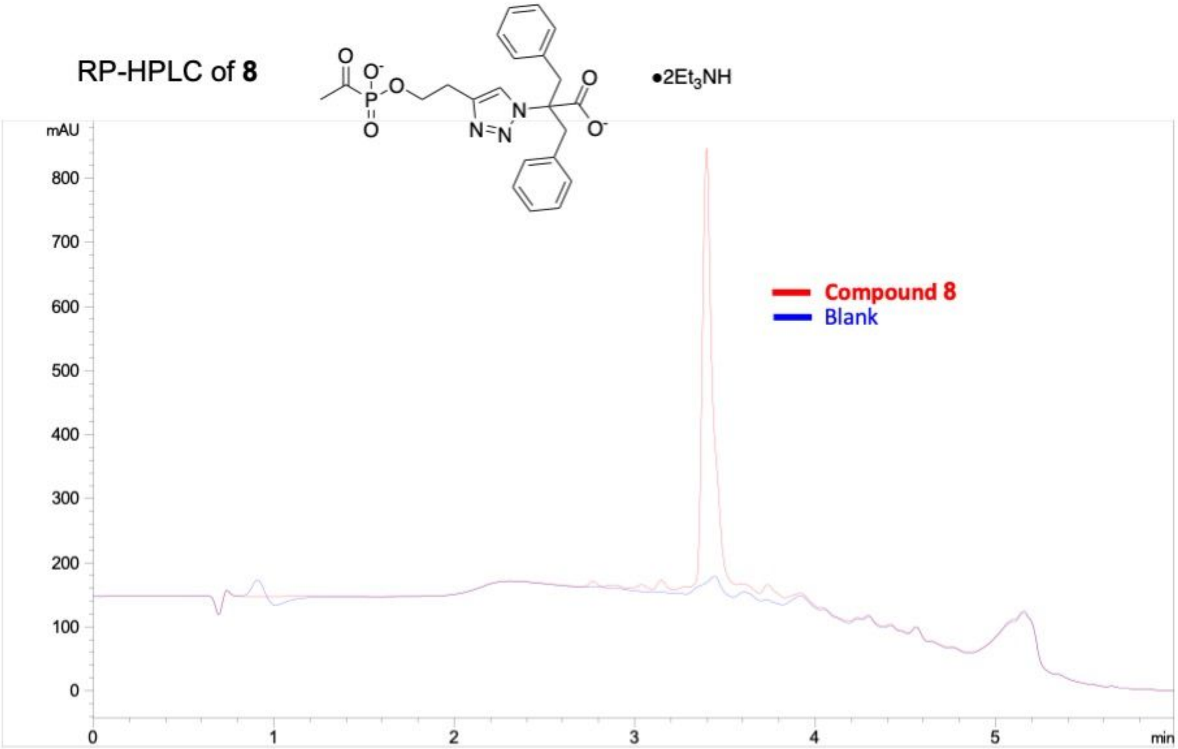

HRMS of **8**

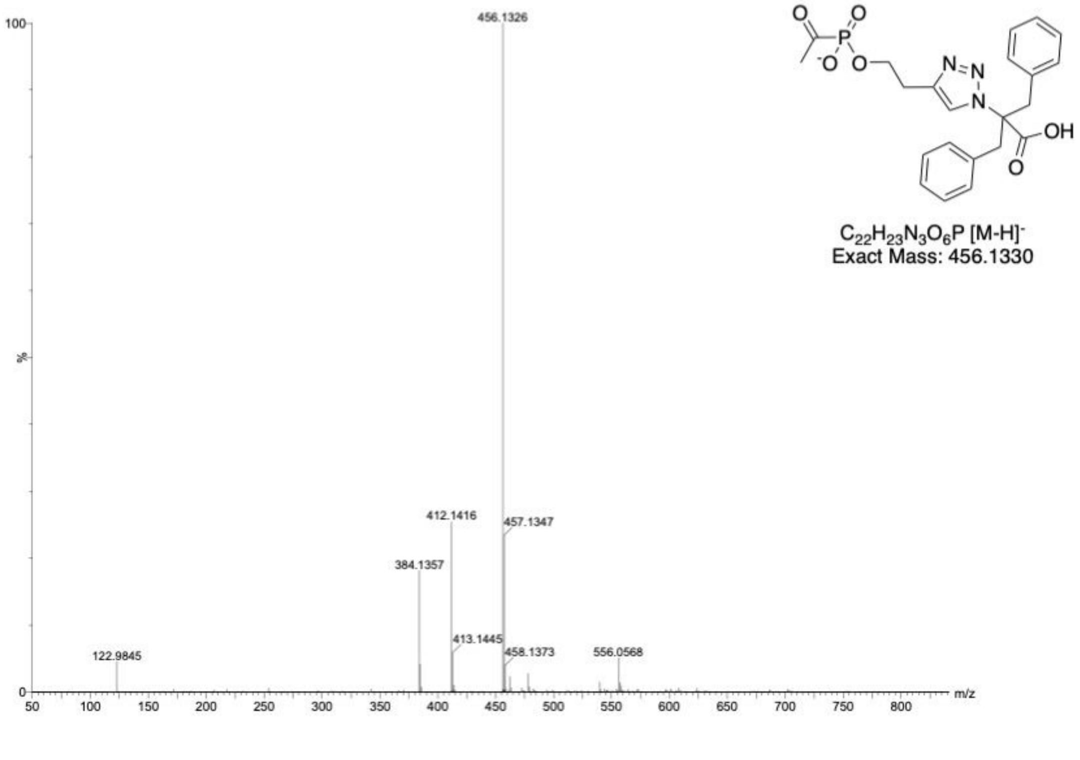

^{1}H NMR of **10**

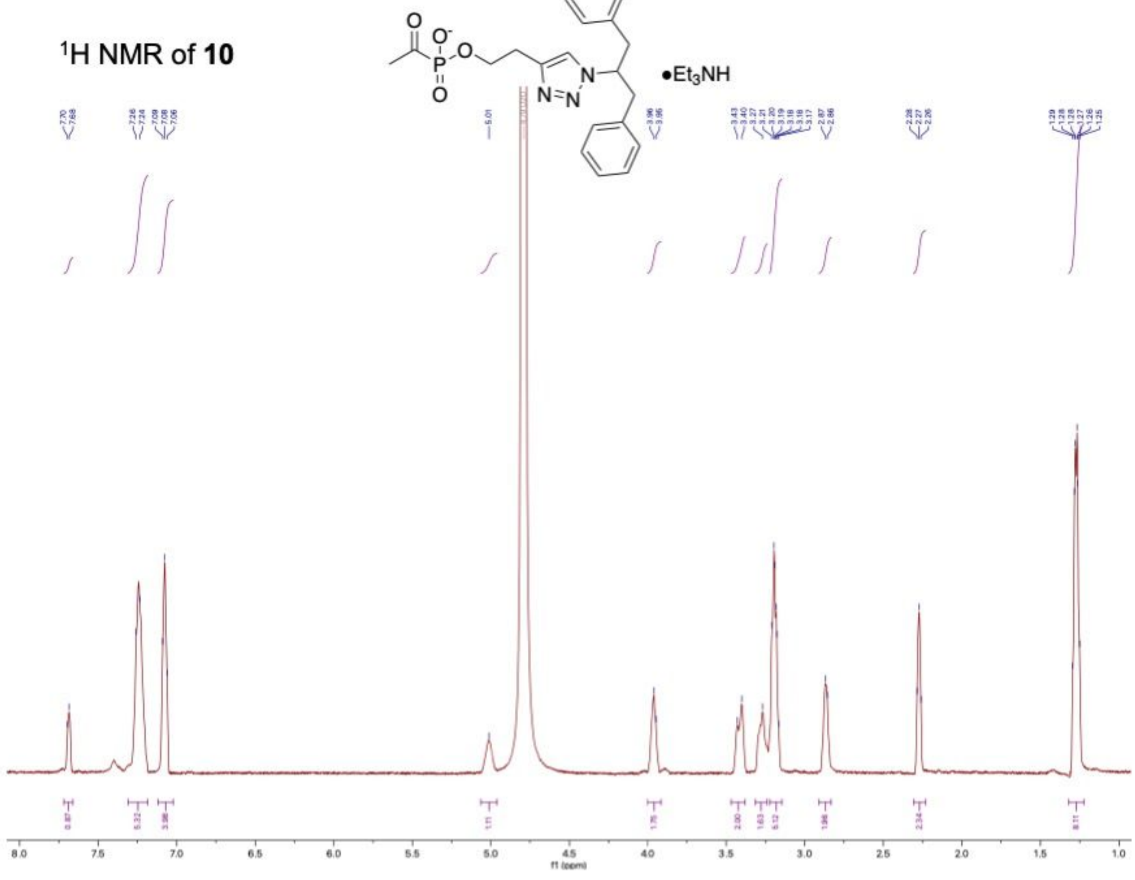

^{31}P NMR of **10**

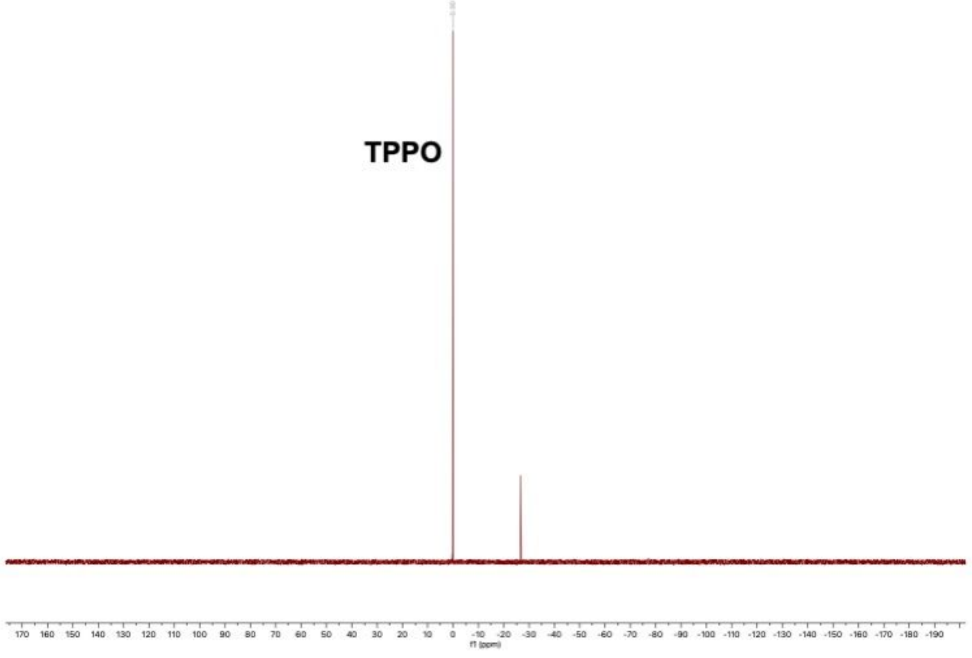

RP-HPLC of **10**

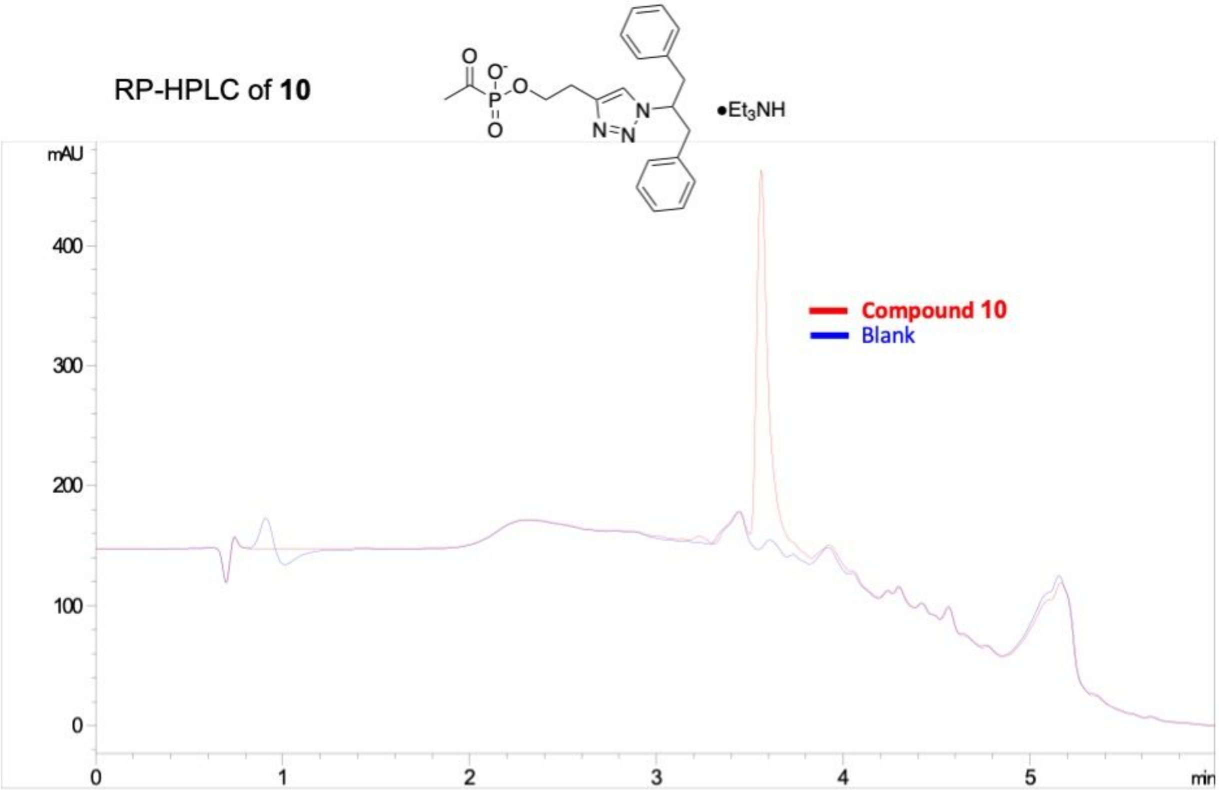

HRMS of **10**

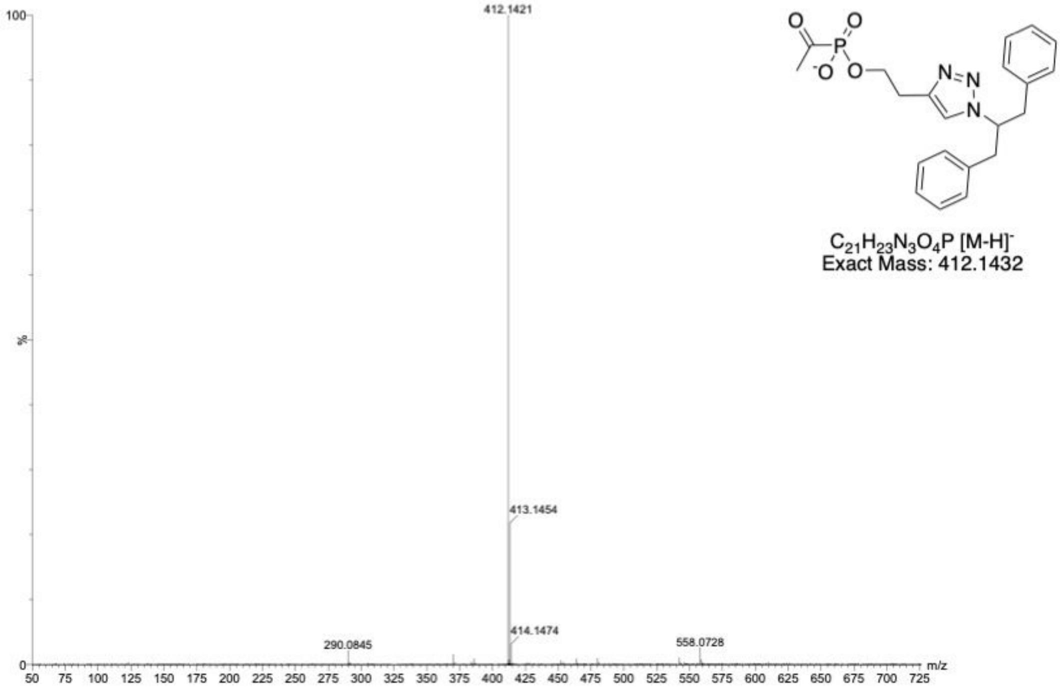

^{1}H NMR of **11**

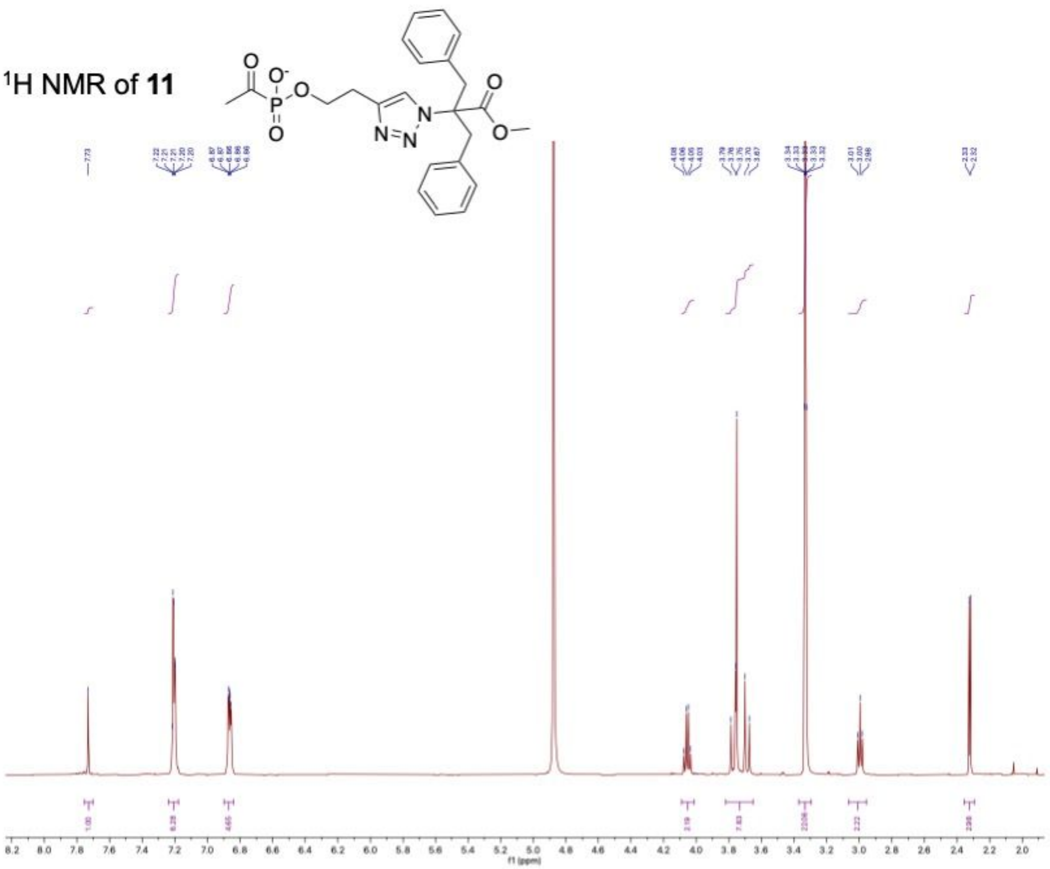

^{31}P NMR of **11**

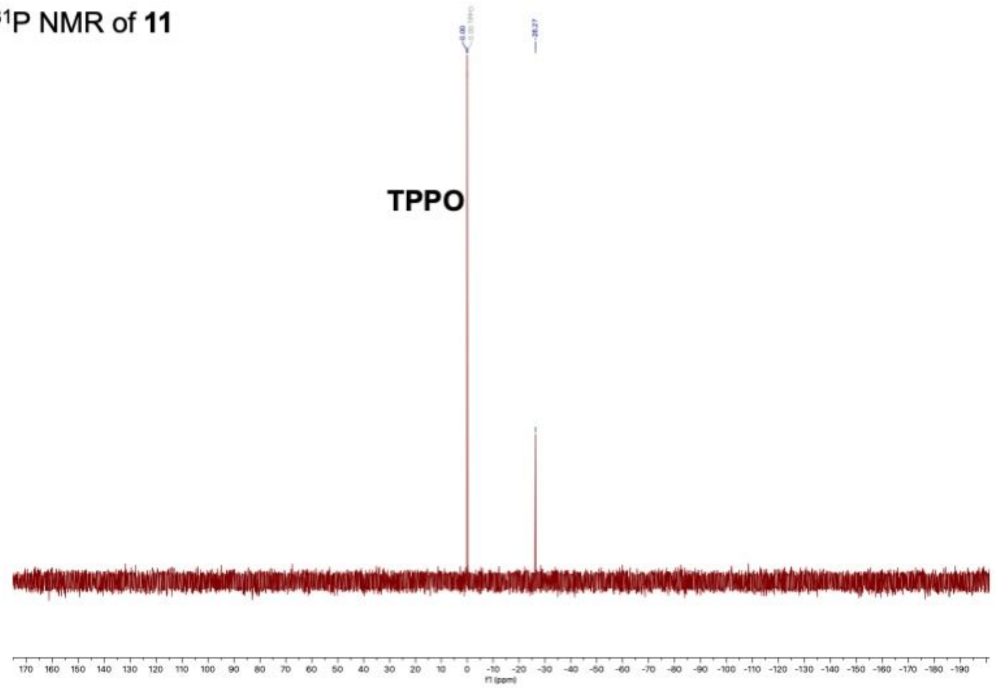

RP-HPLC of **11**

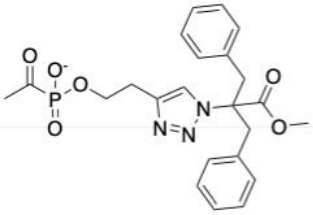

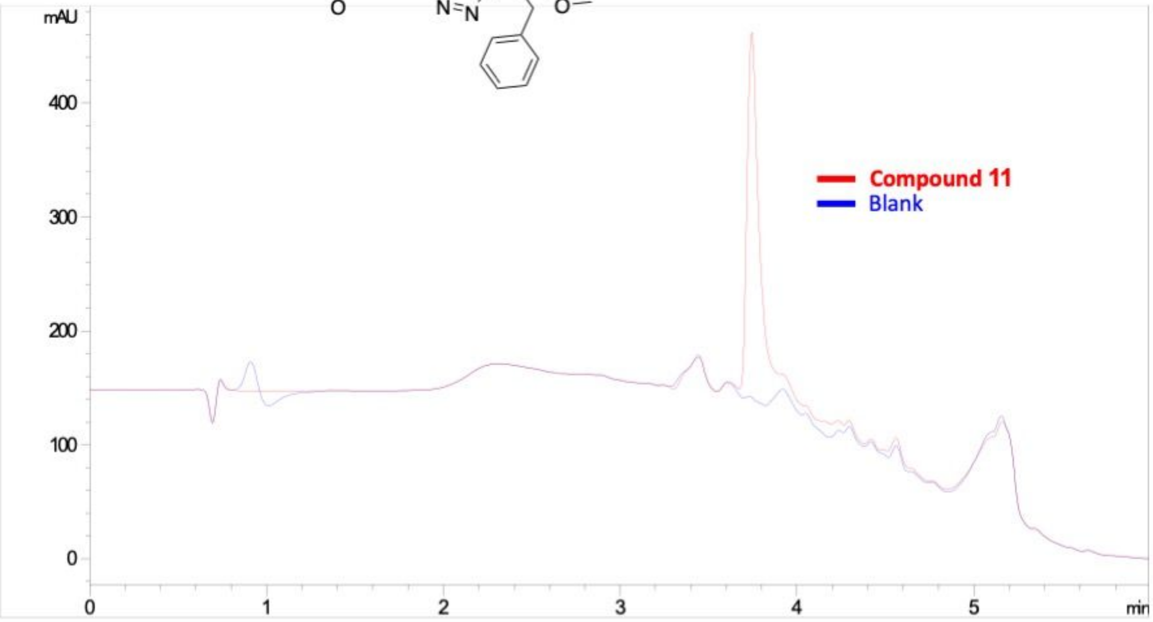

HRMS of **11**

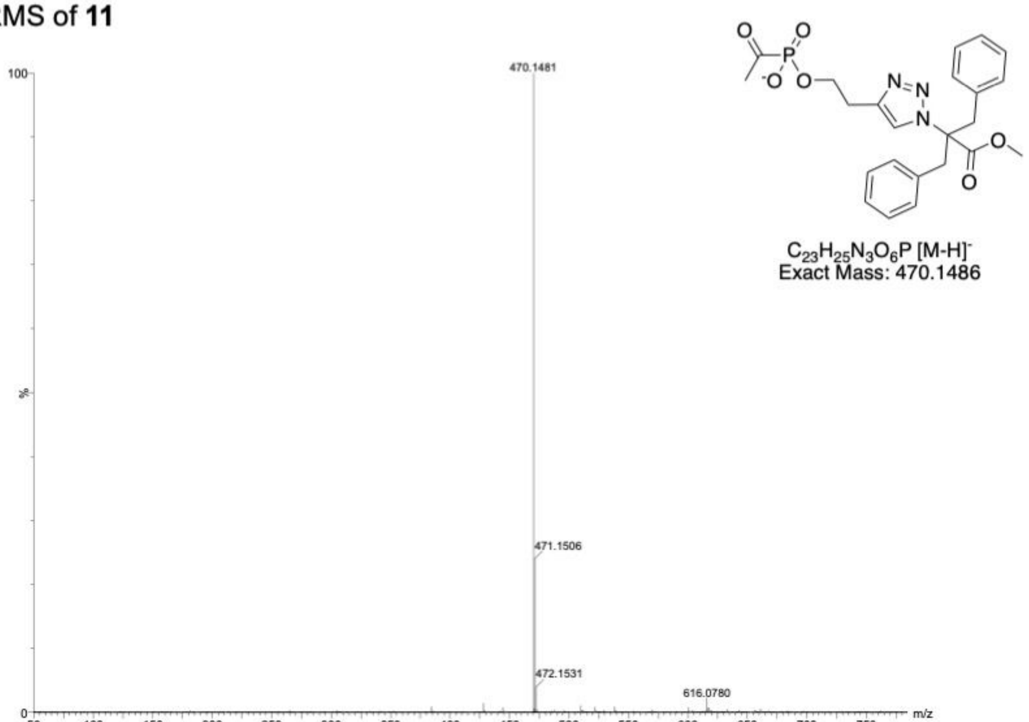

$C_{23}H_{25}N_3O_6P$ [M-H]$^-$
Exact Mass: 470.1486

^{1}H NMR of **12**

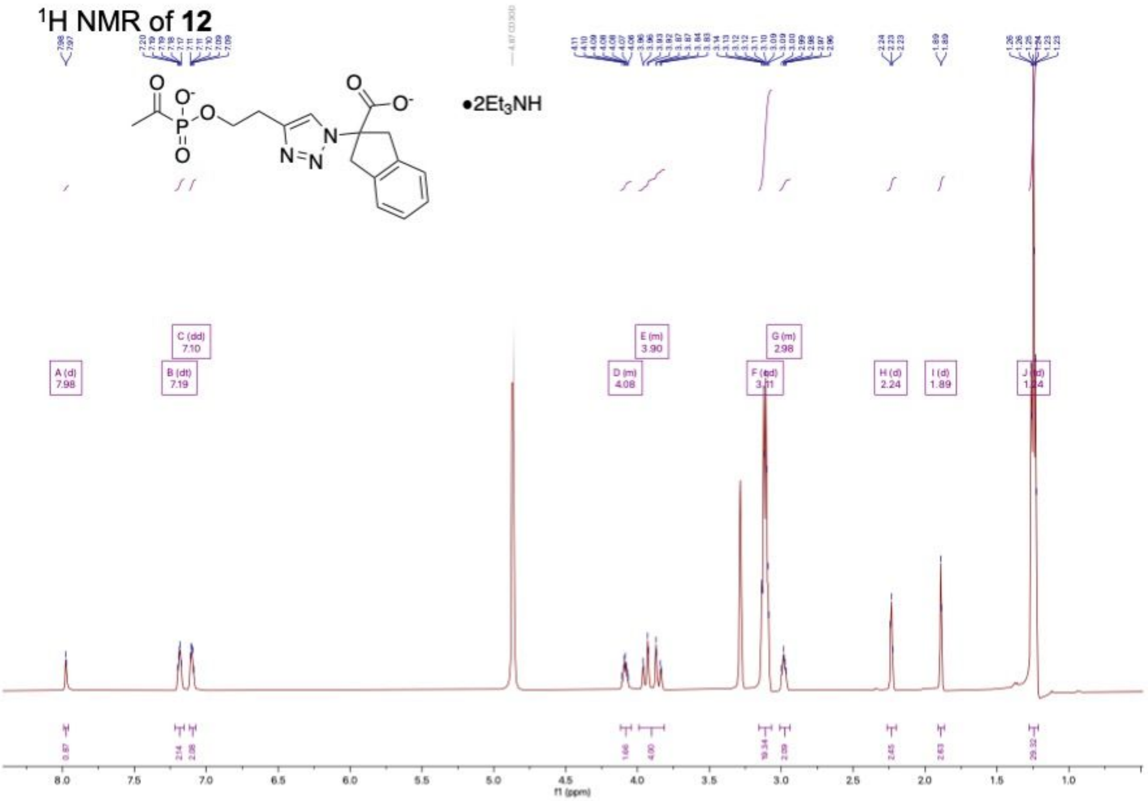

^{31}P NMR of **12**

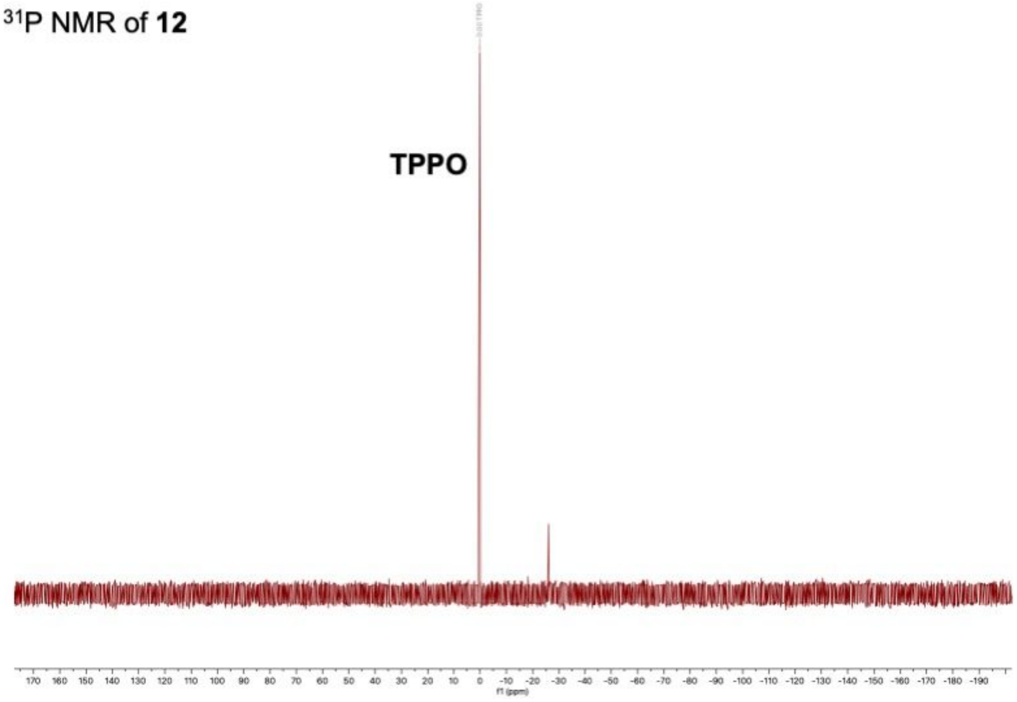

RP-HPLC of **12**

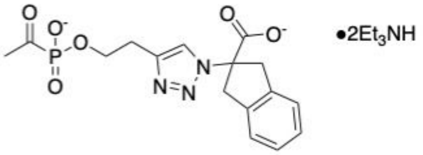

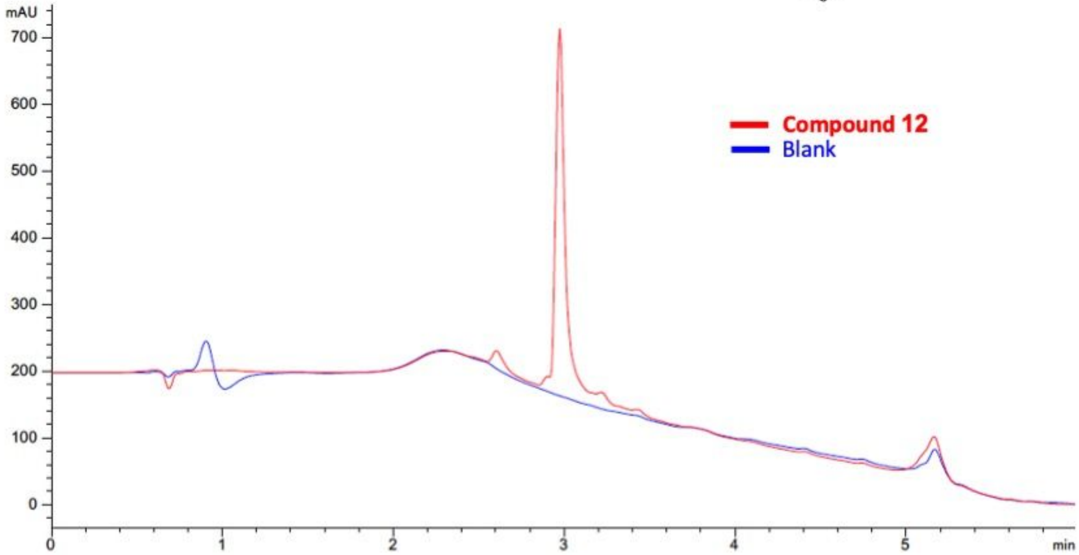

HRMS of **12**

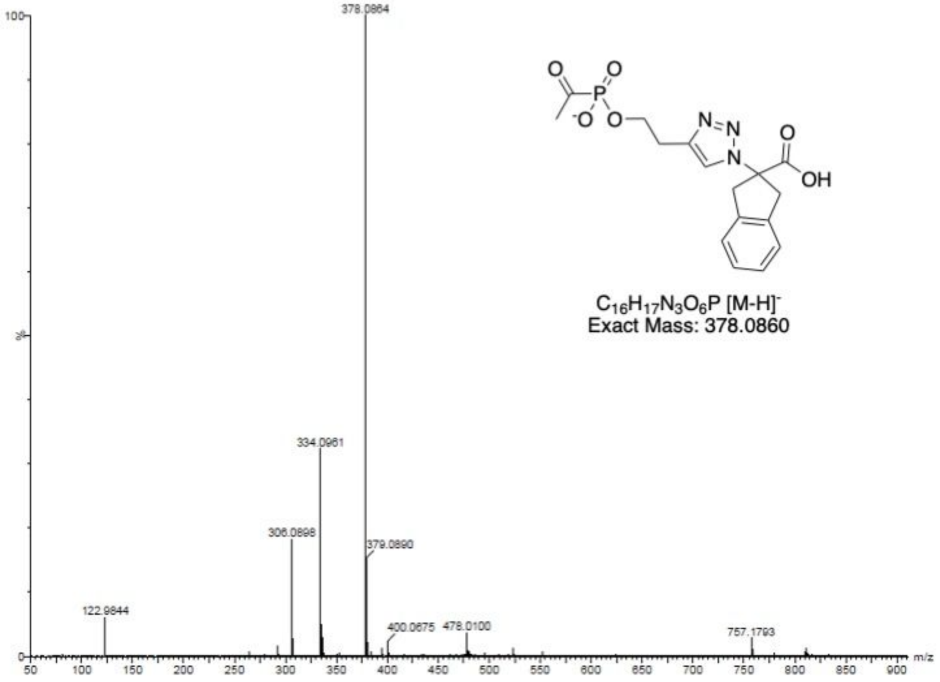

^{1}H NMR of **13**

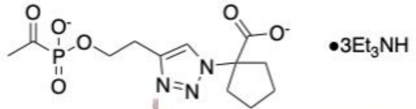

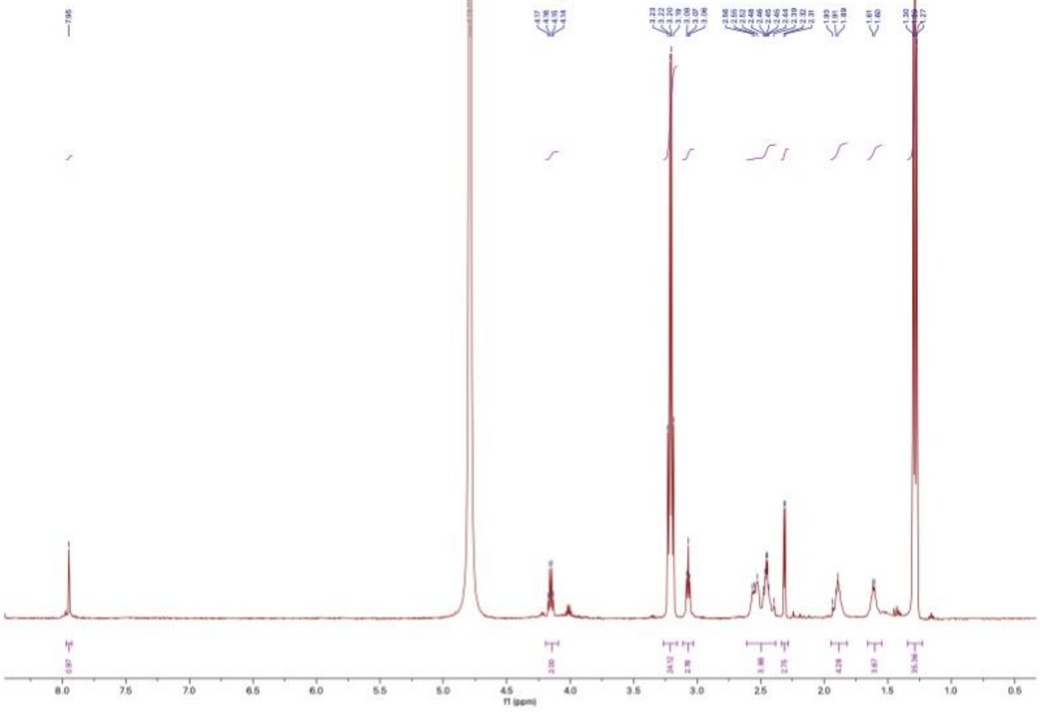

^{31}P NMR of **13**

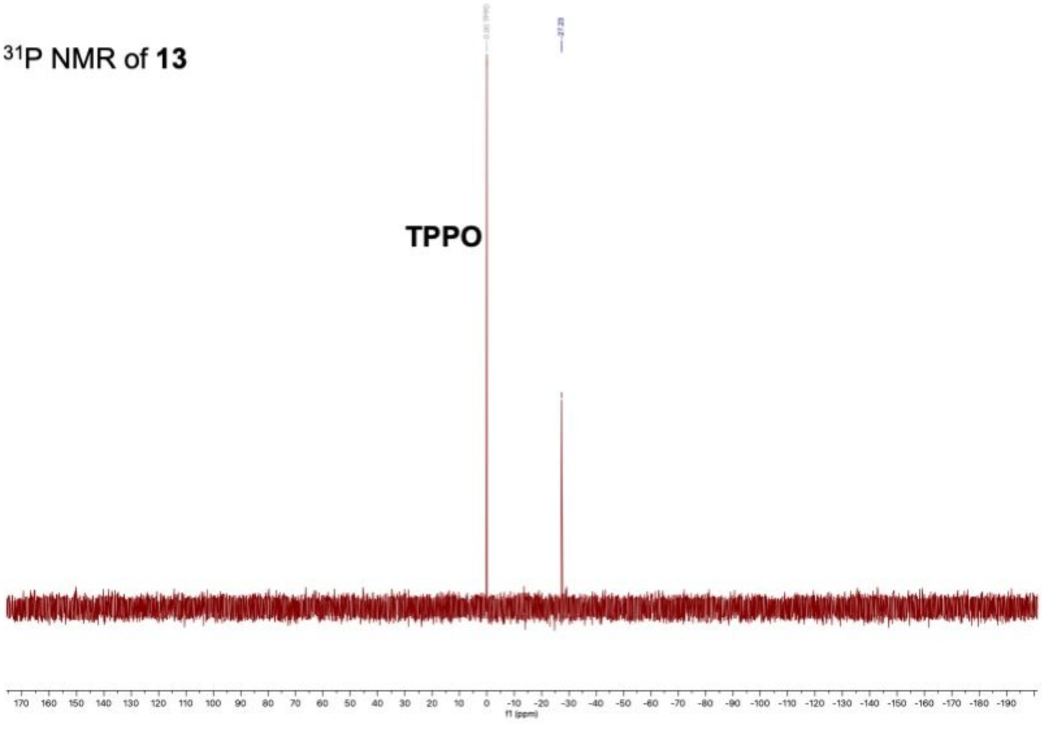

RP-HPLC of **13**

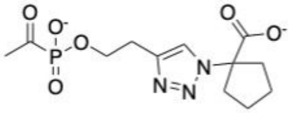

•$3Et_3NH$

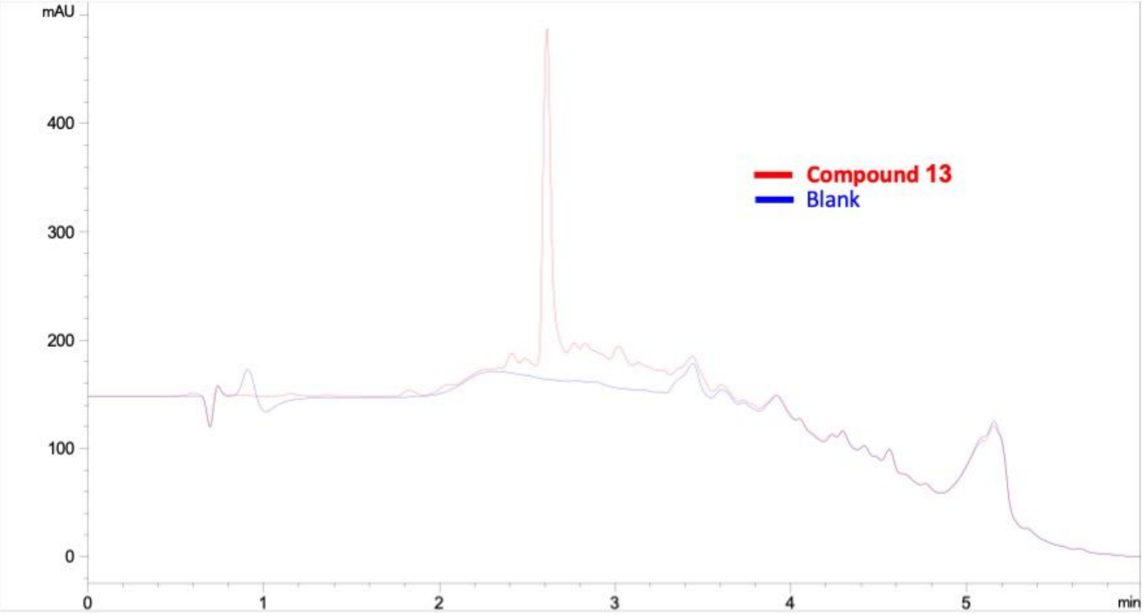

HRMS of **13**

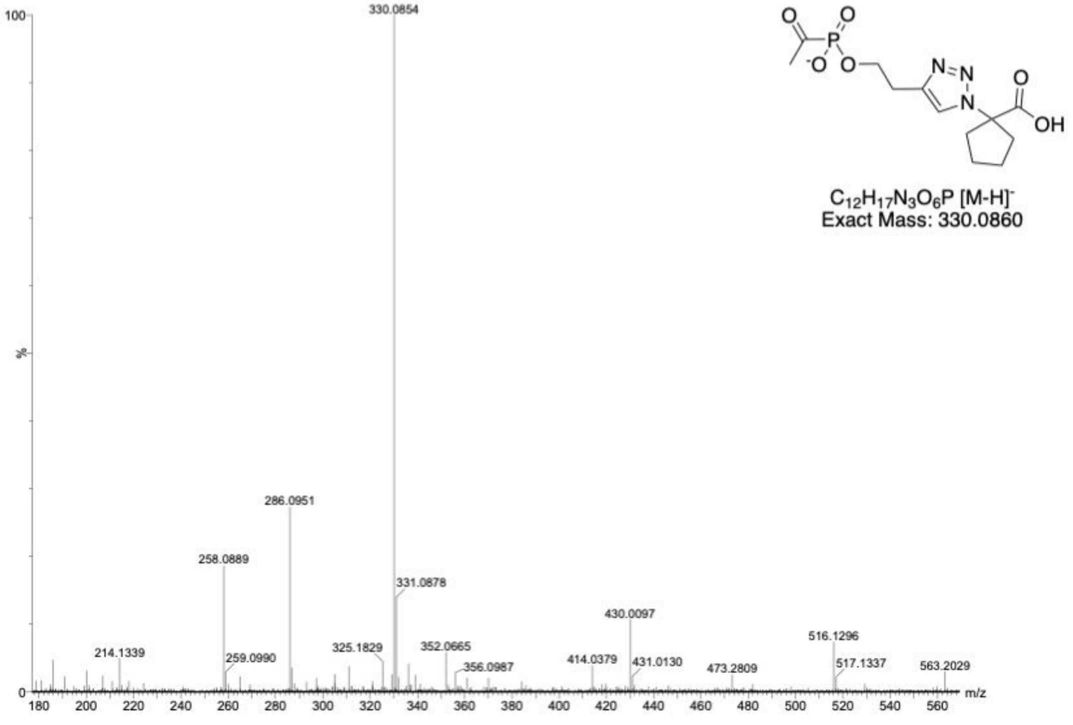

$C_{12}H_{17}N_3O_6P$ $[M\text{-}H]^-$
Exact Mass: 330.0860

^{1}H NMR of **14**

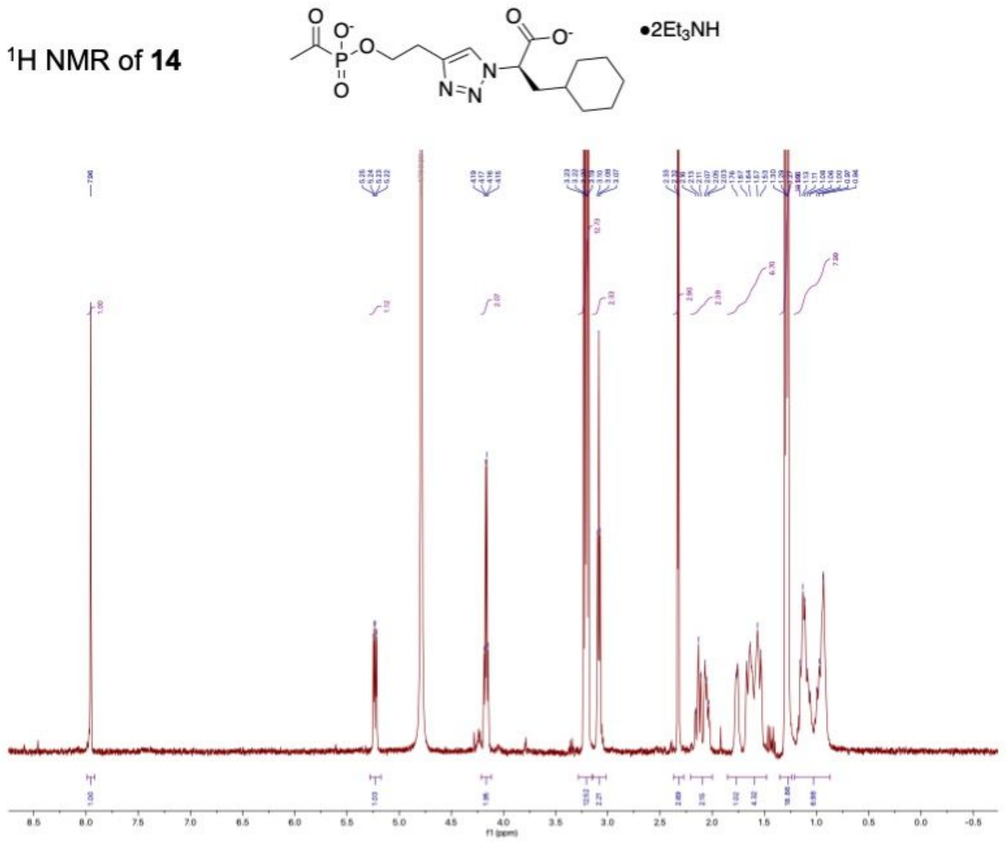

^{31}P NMR of **14**

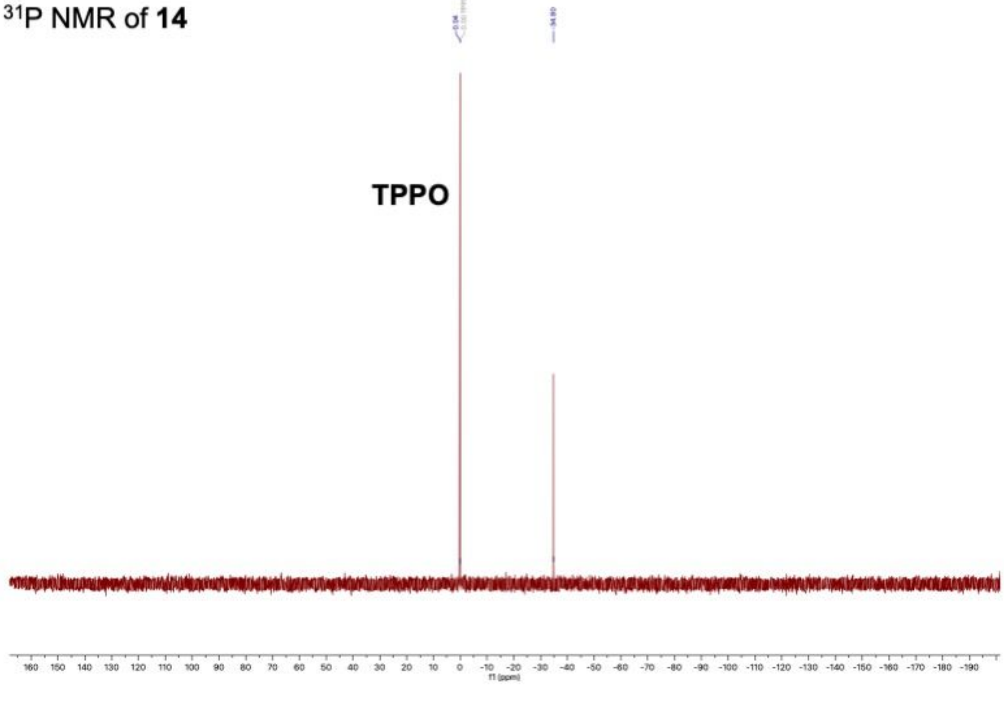

RP-HPLC of **14**

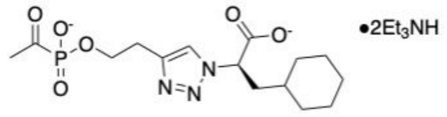

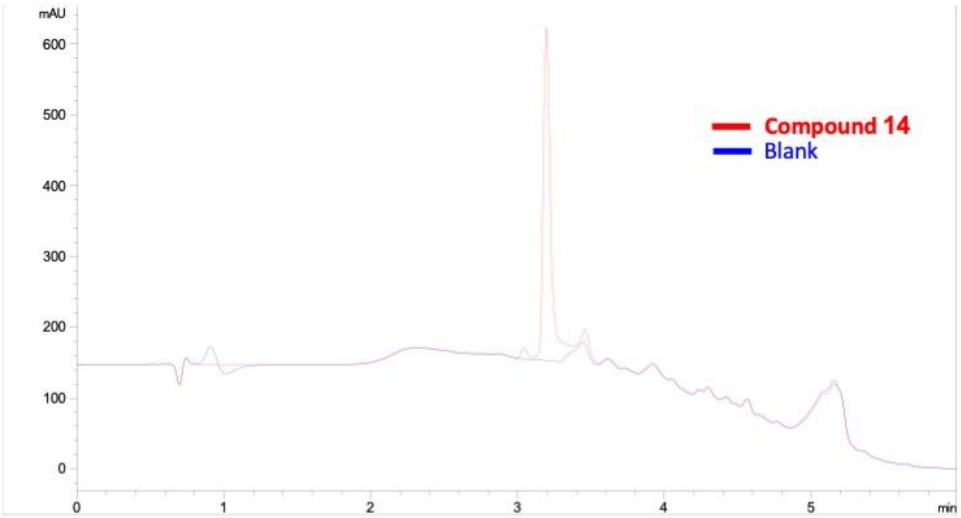

HRMS of **14**

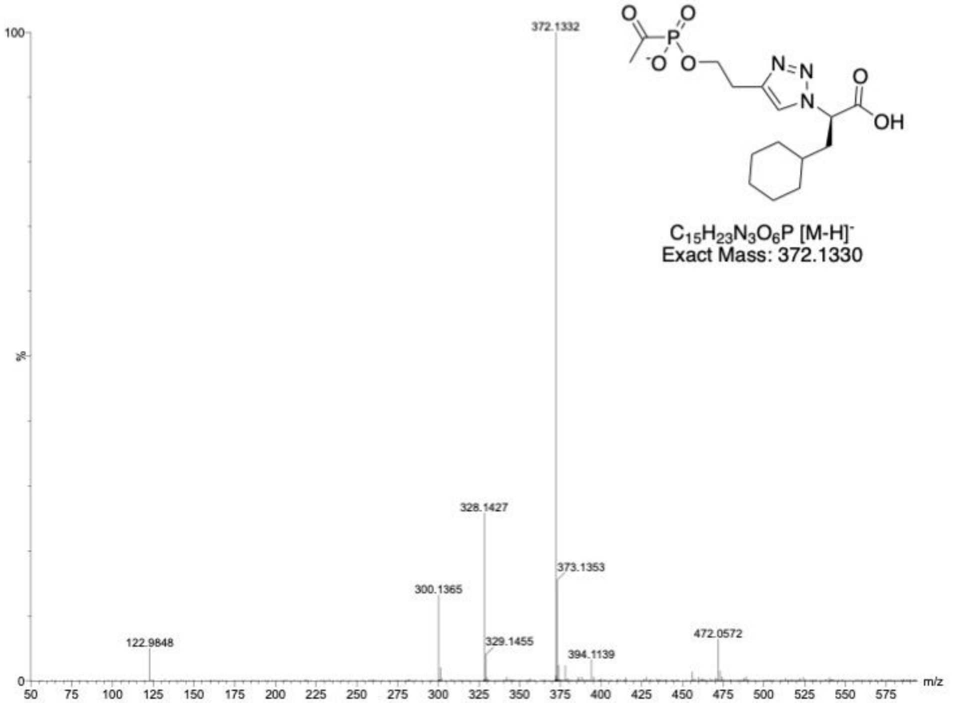

^{1}H NMR of **15**

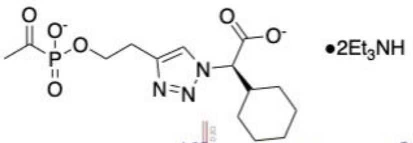

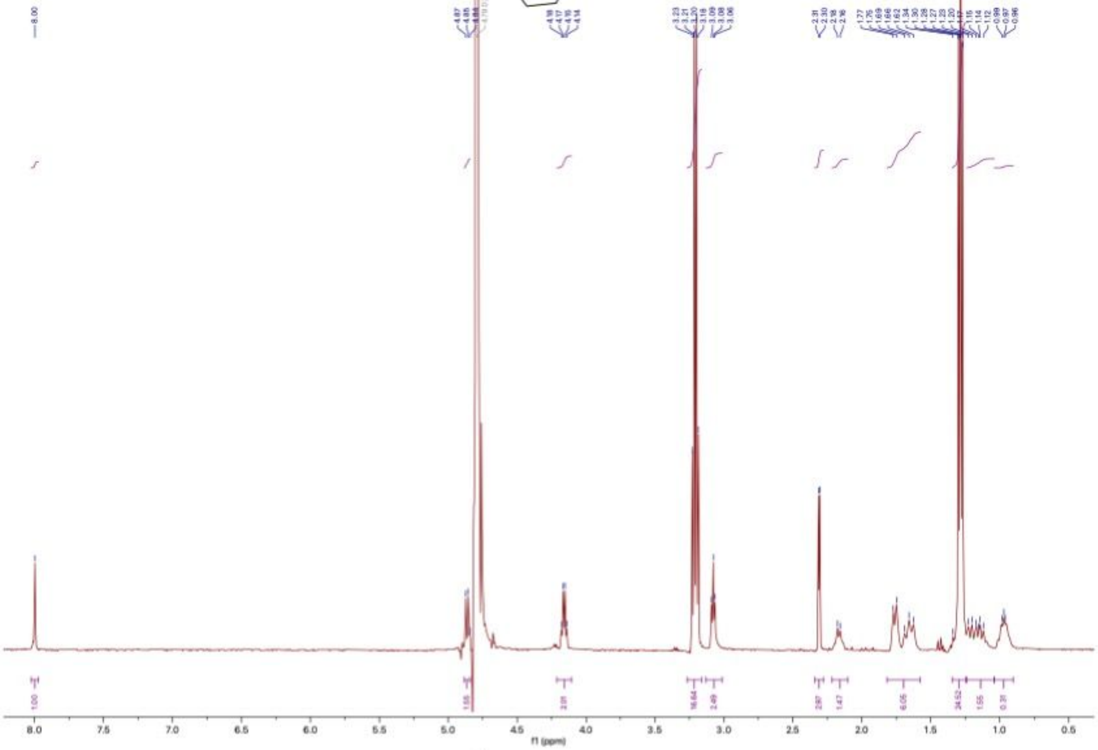

^{31}P NMR of **15**

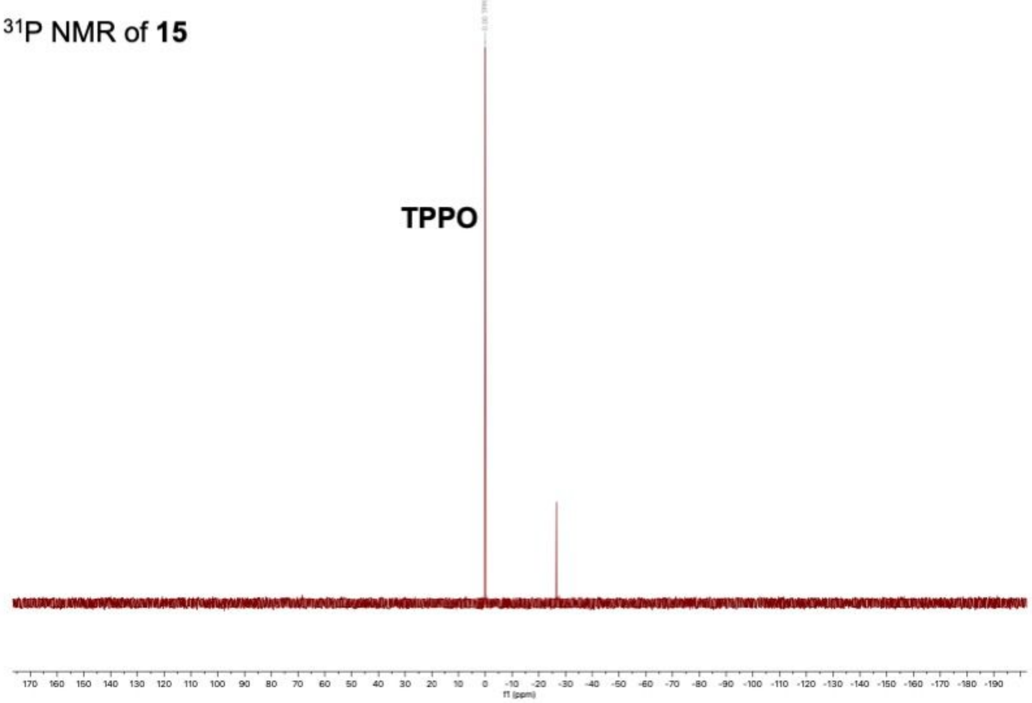

RP-HPLC of **15**

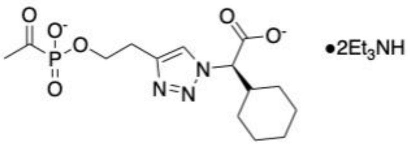

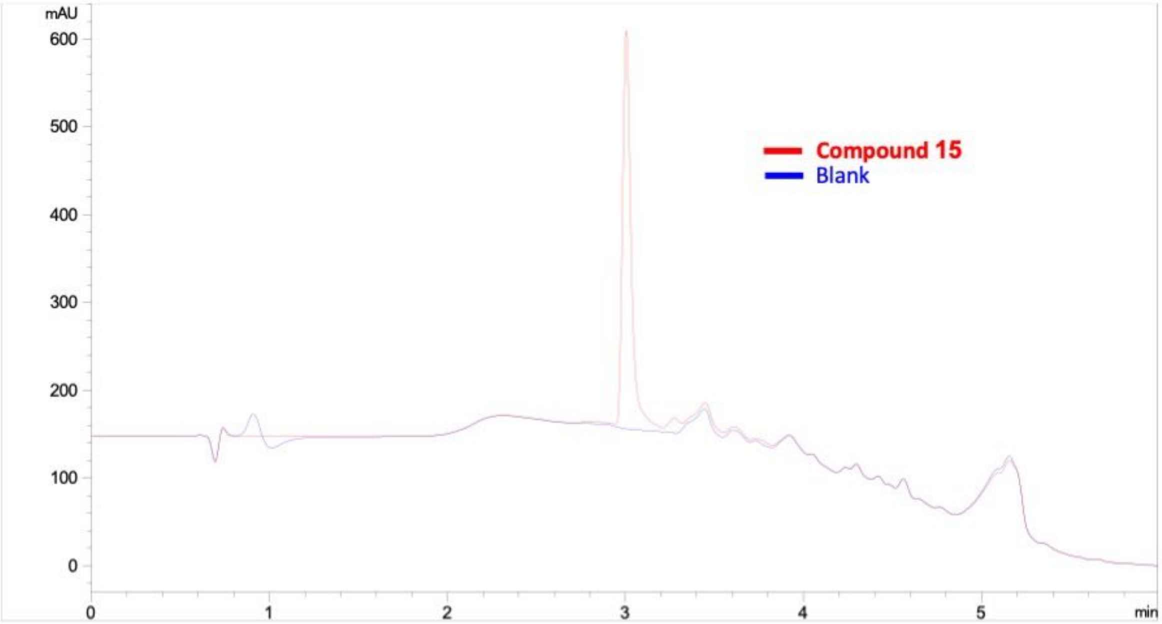

HRMS of **15**

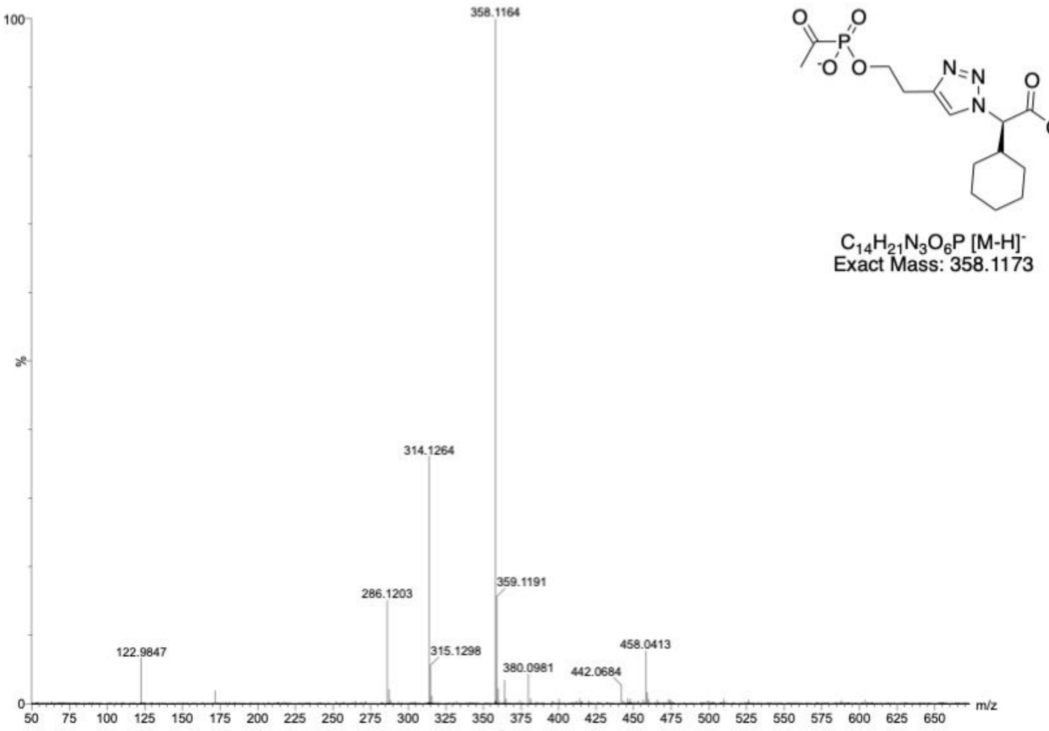

^{1}H NMR of **16**

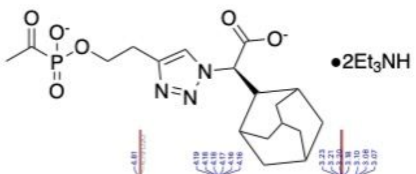

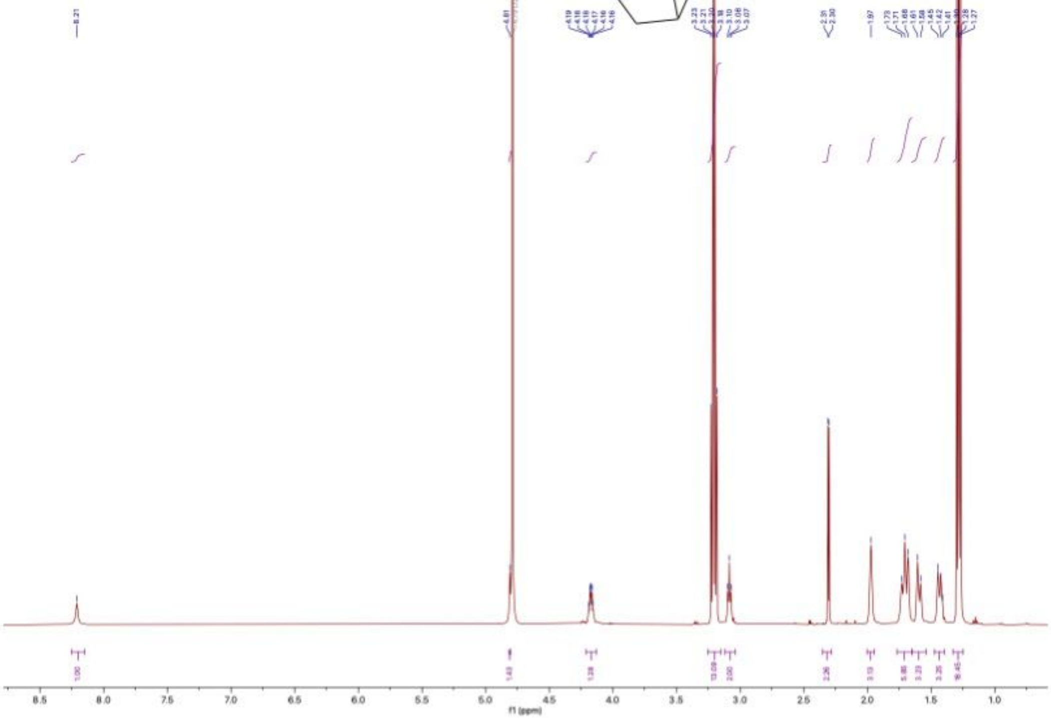

^{31}P NMR of **16**

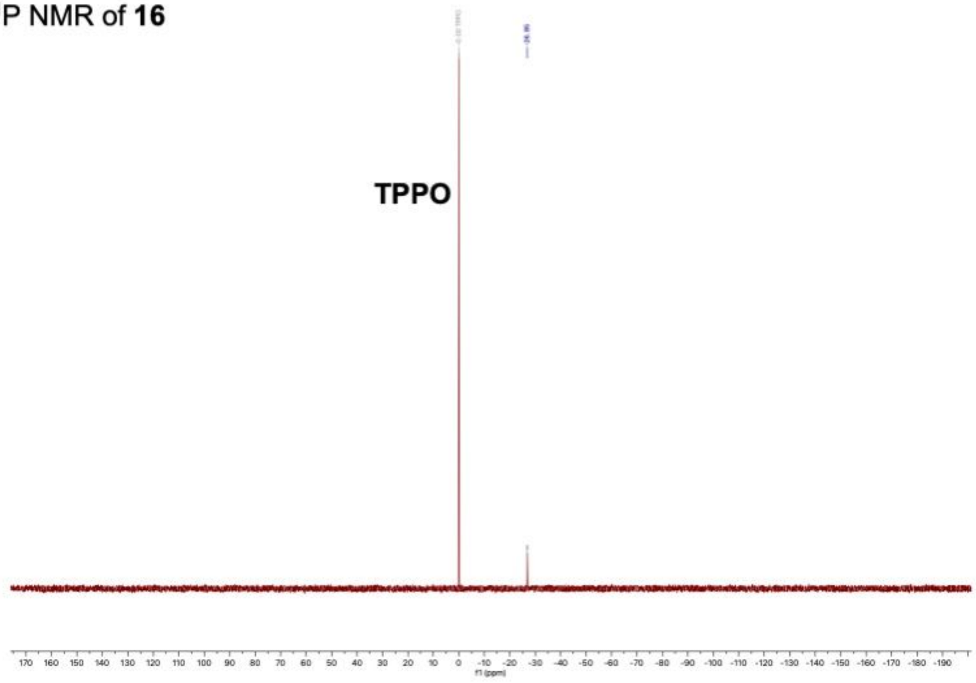

RP-HPLC of **16**

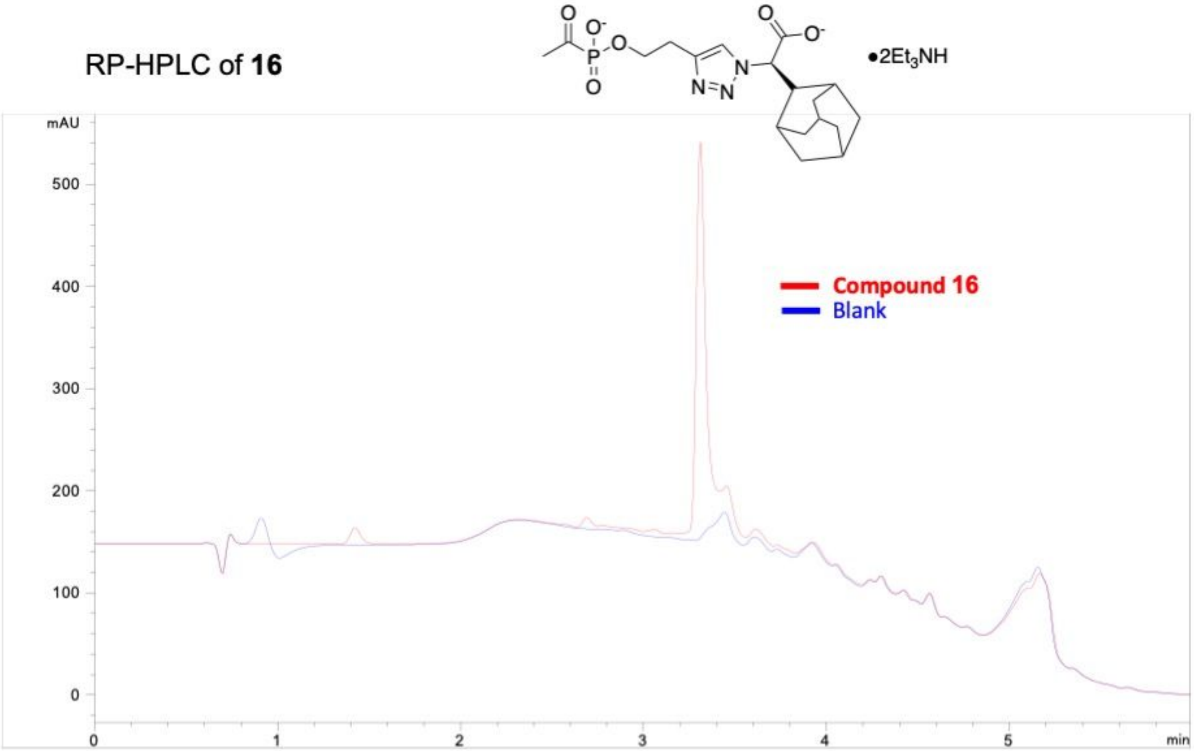

HRMS of **16**

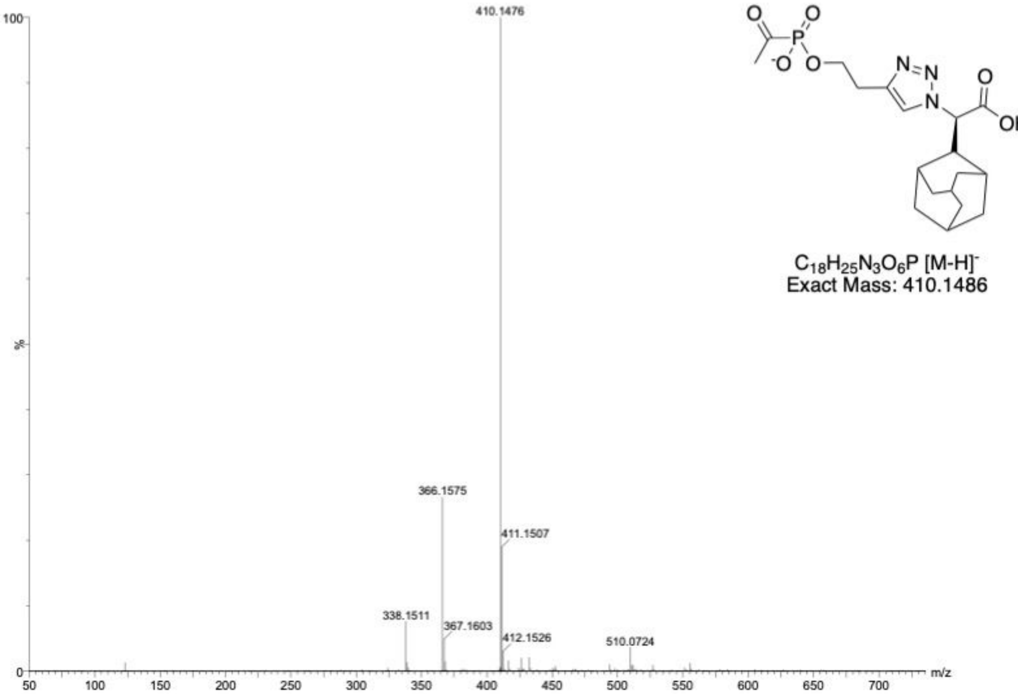

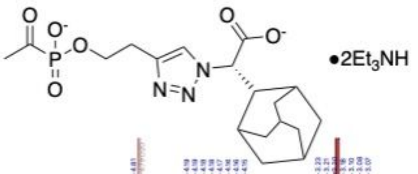

^1H NMR of **17**

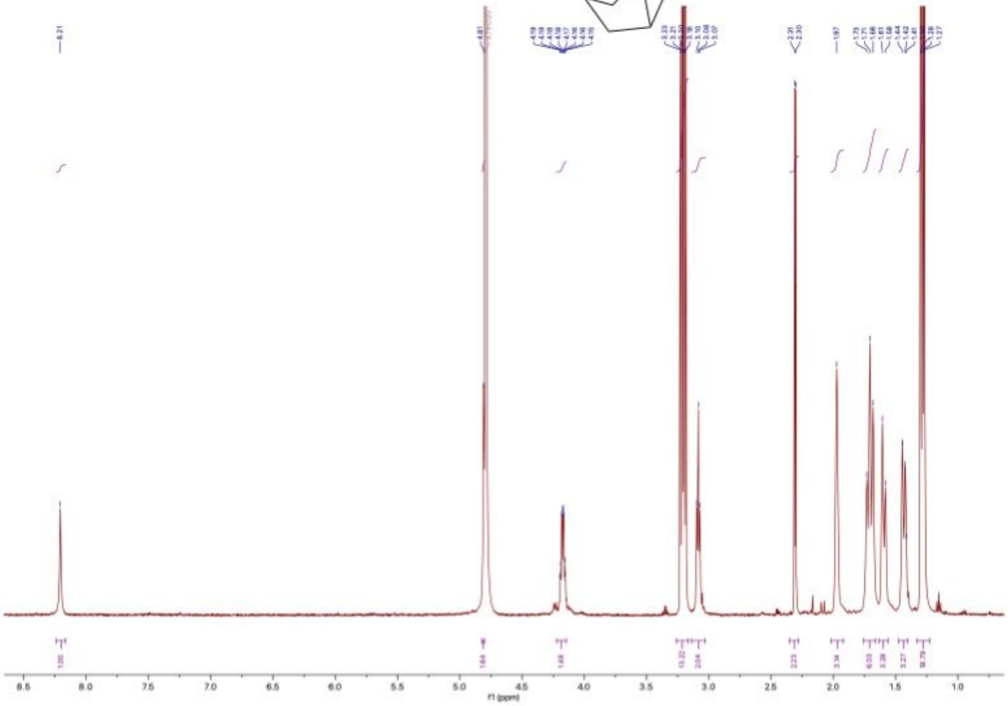

^{31}P NMR of **17**

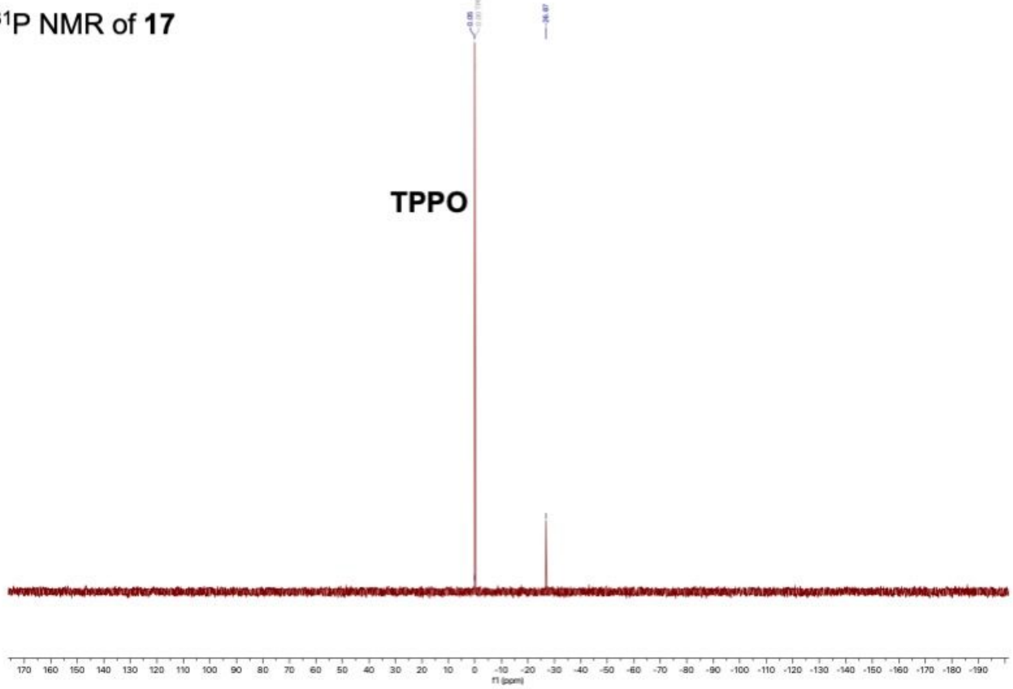

RP-HPLC of **17**

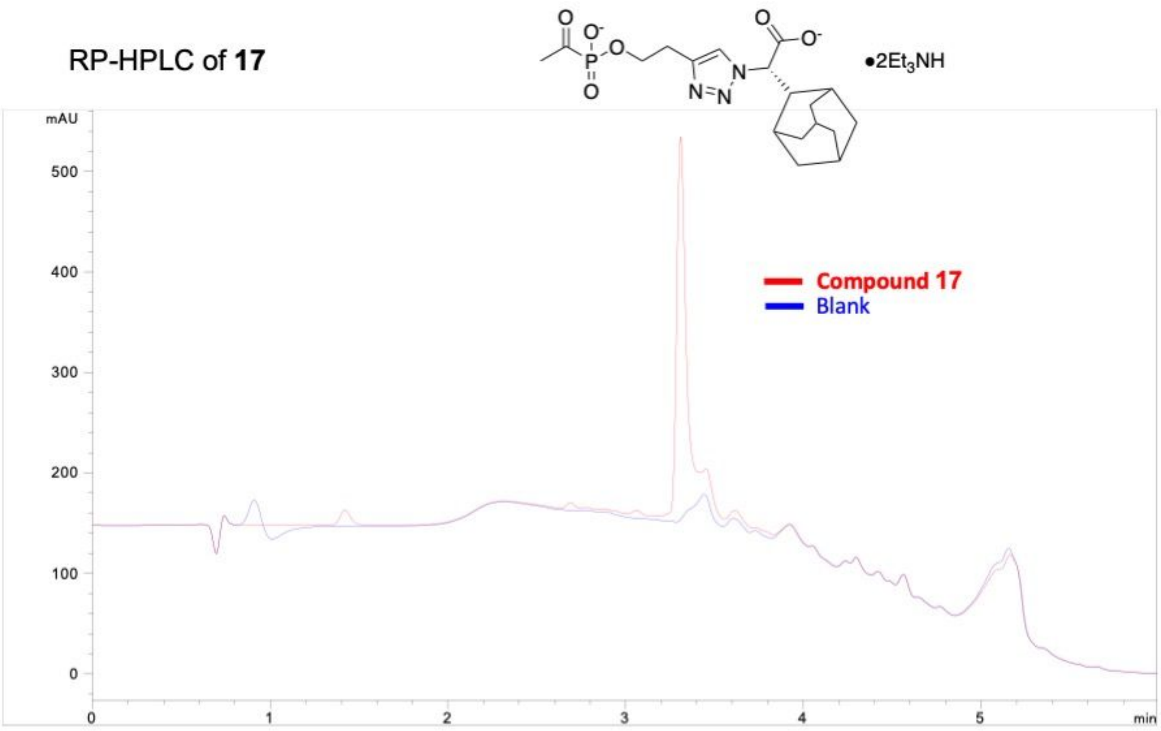

HRMS of **17**

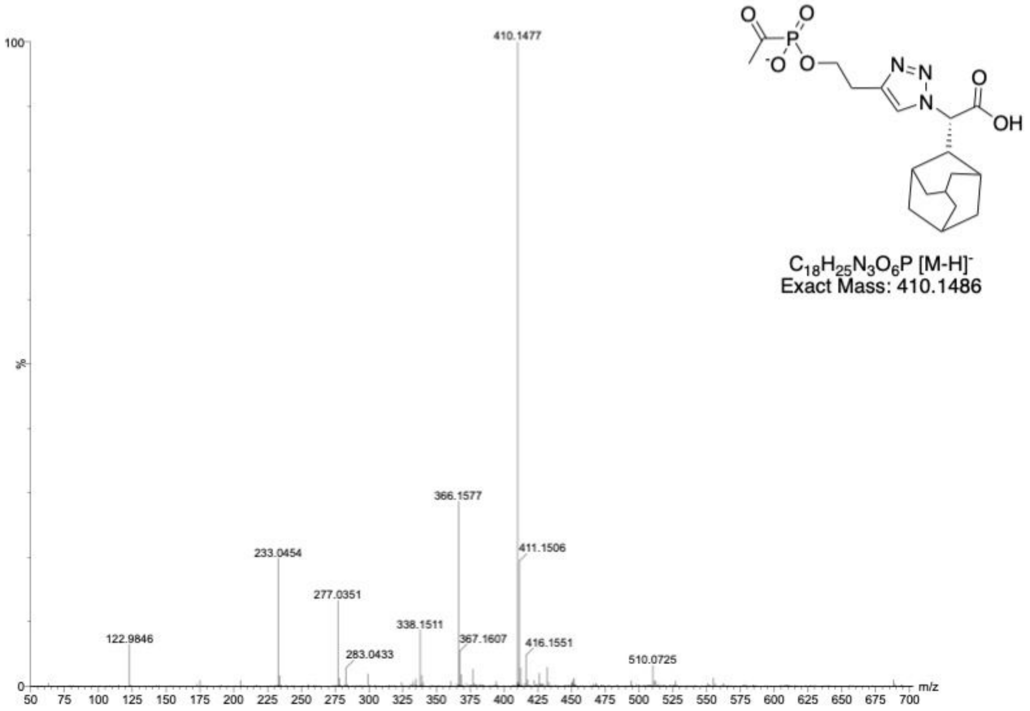

^{1}H NMR of **18**

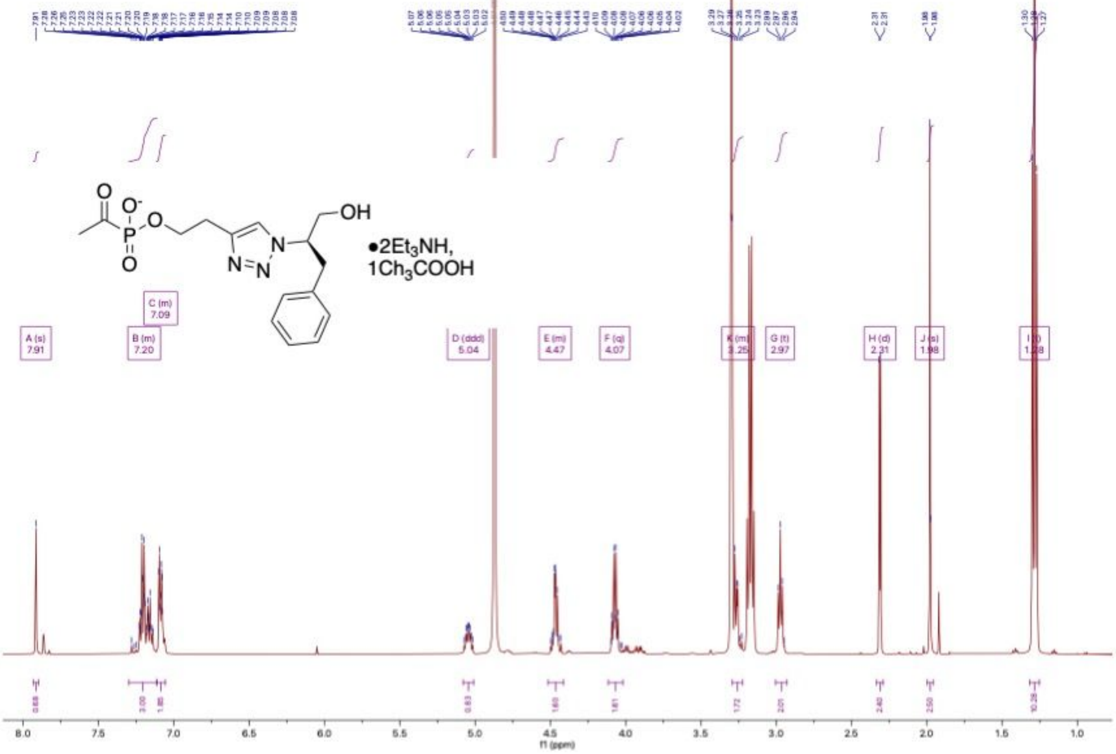

^{31}P NMR of **18**

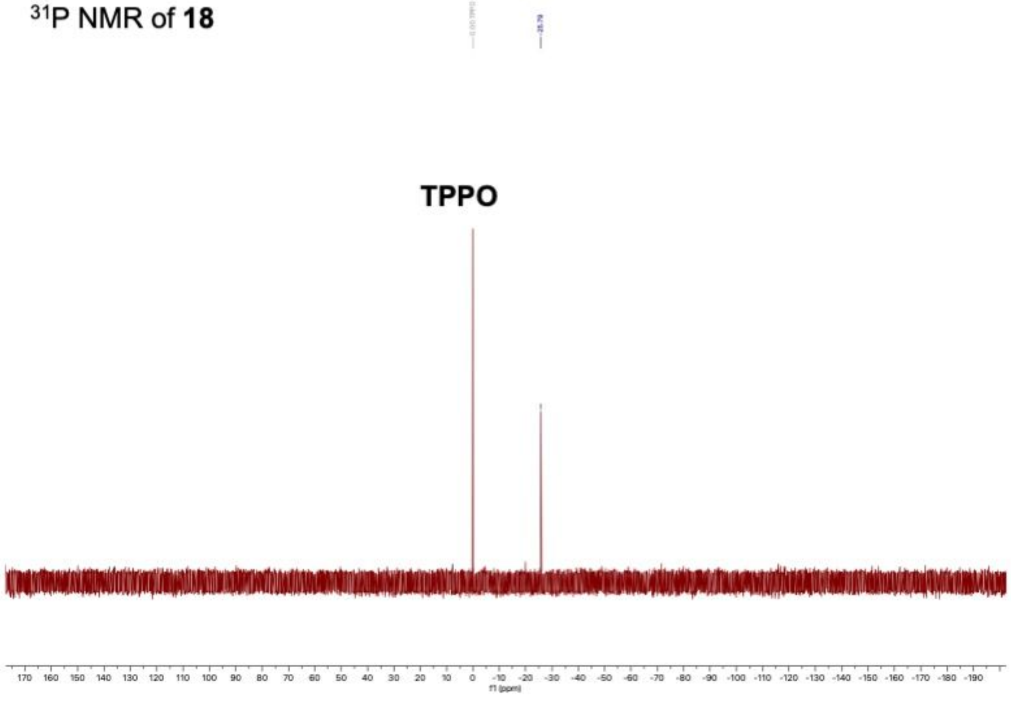

RP-HPLC of **18**

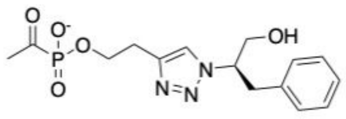

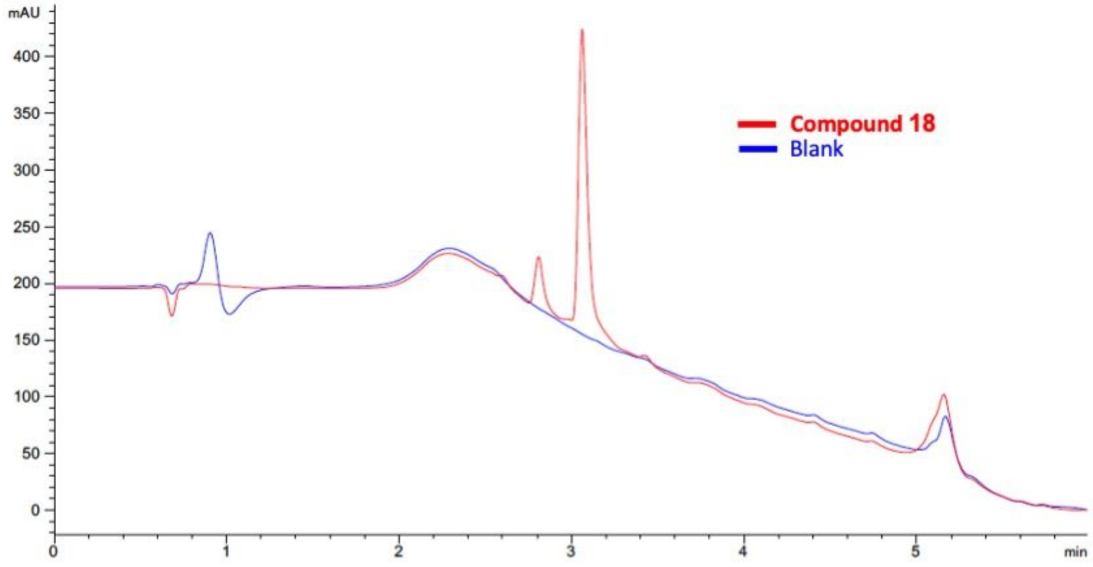

HRMS of **18**

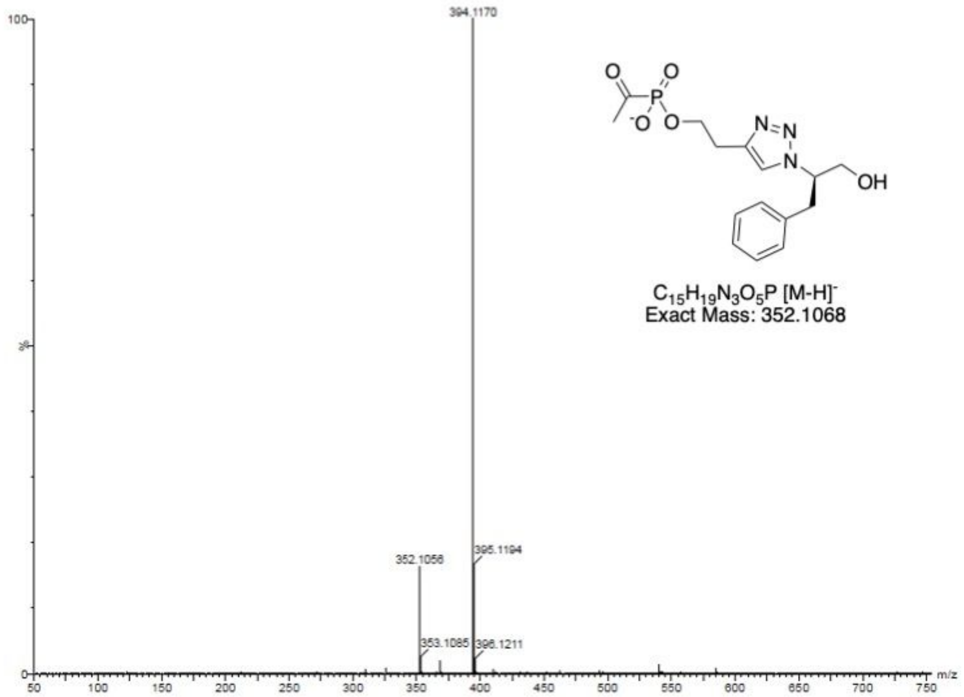

^{1}H NMR of **19**

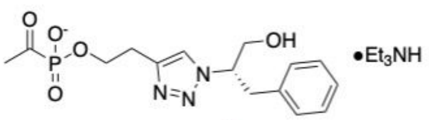

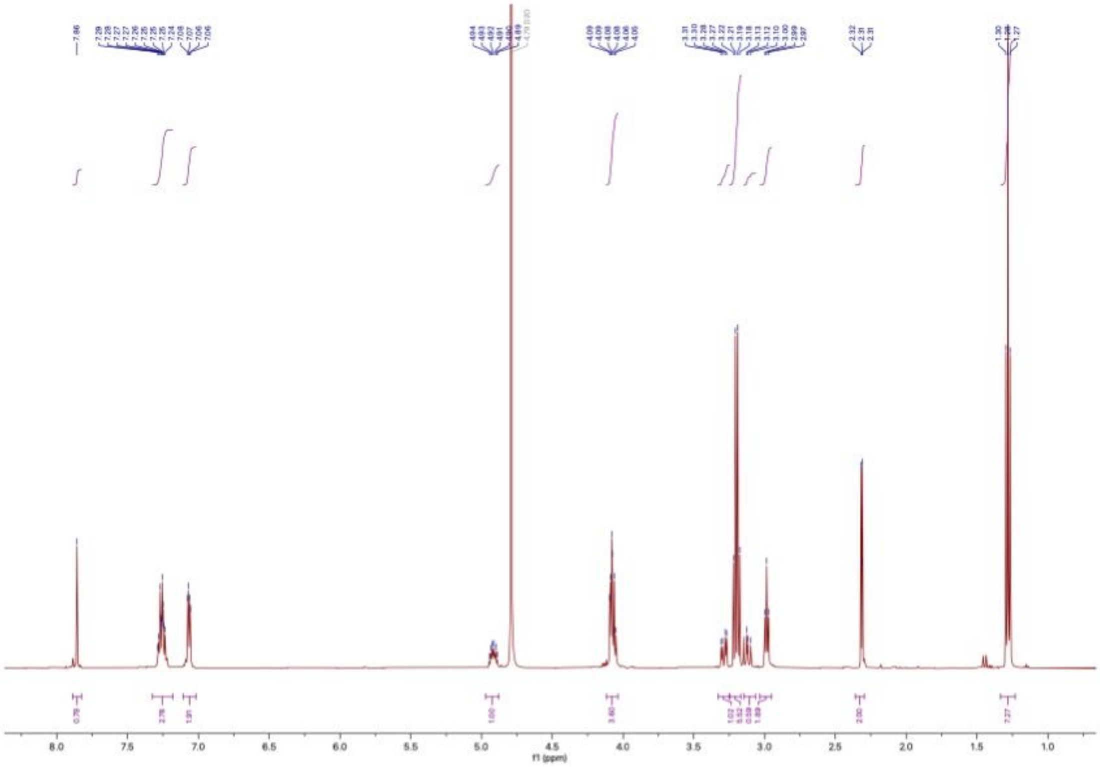

^{31}P NMR of **19**

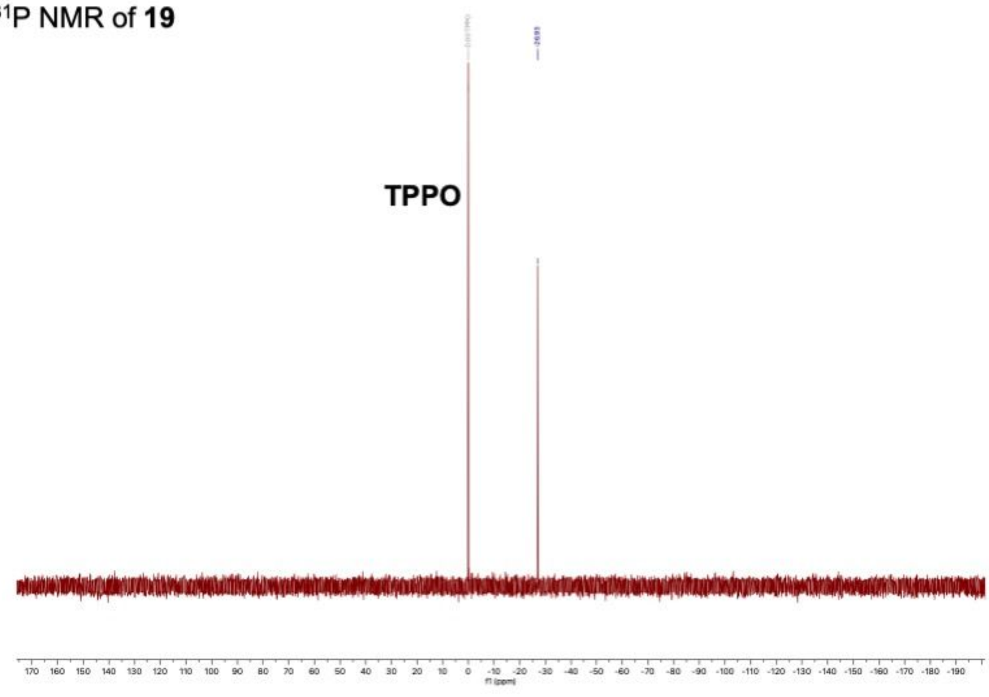

RP-HPLC of **19**

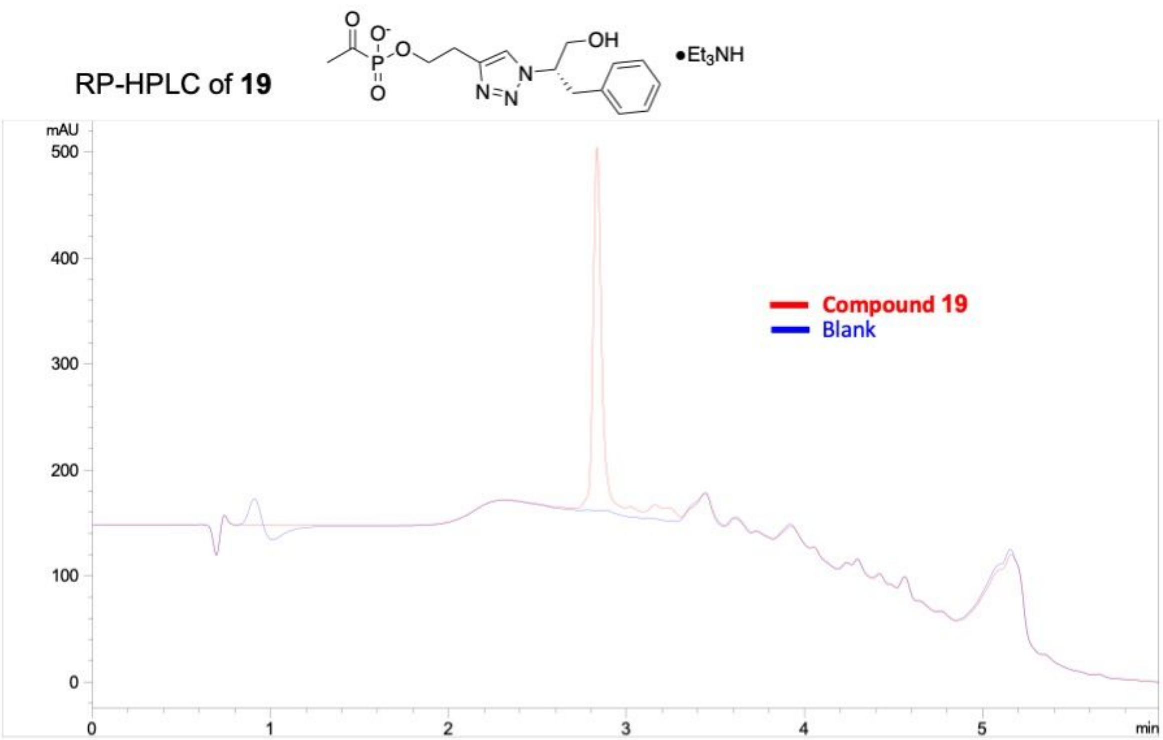

HRMS of **19**

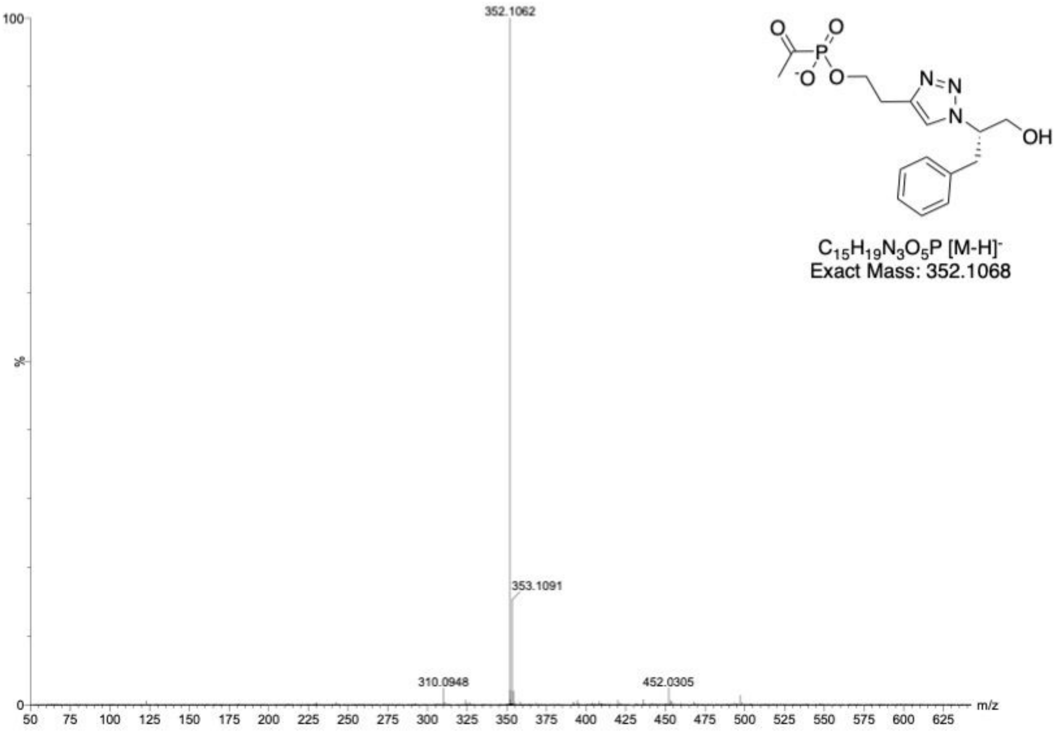

^{1}H NMR of **20**

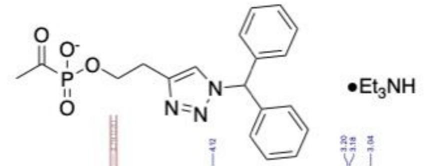

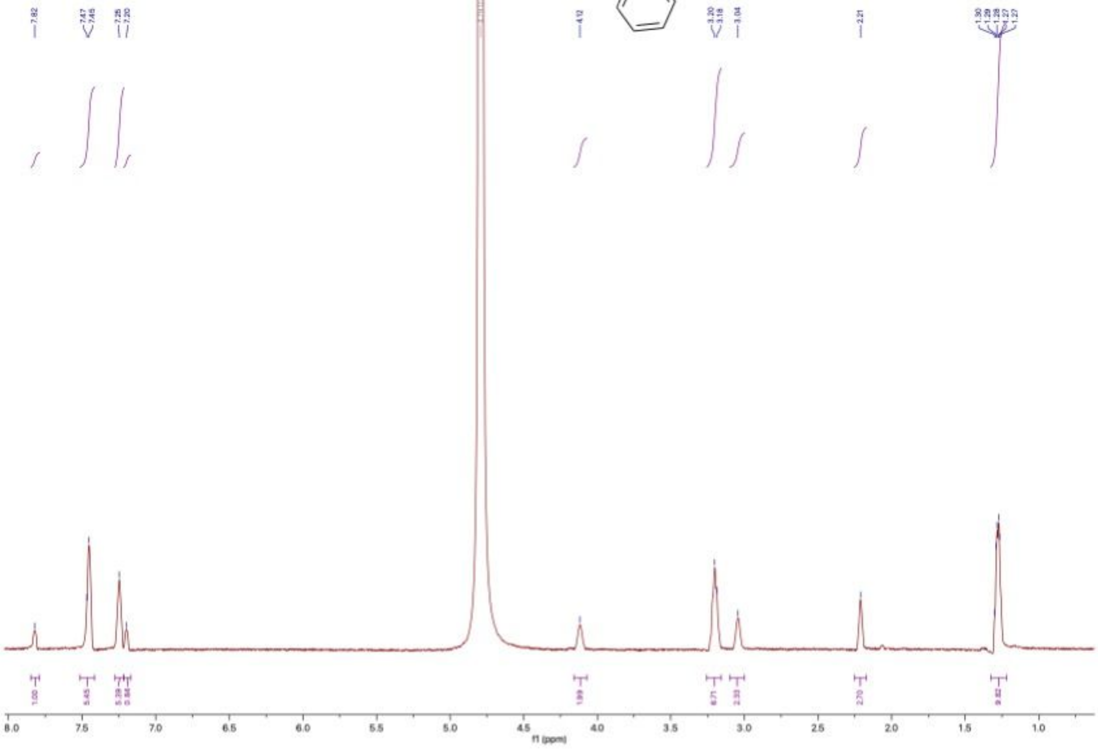

^{31}P NMR of **20**

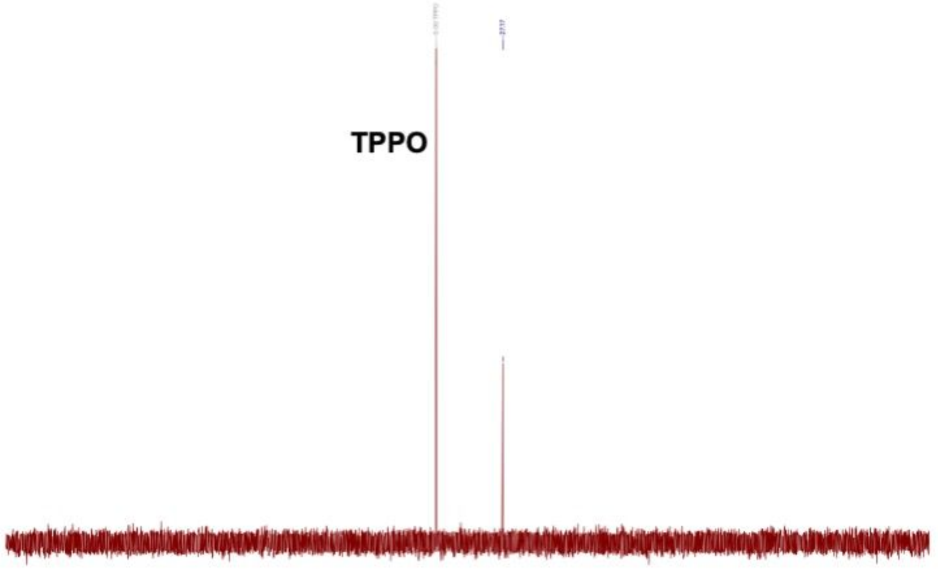

RP-HPLC of **20**

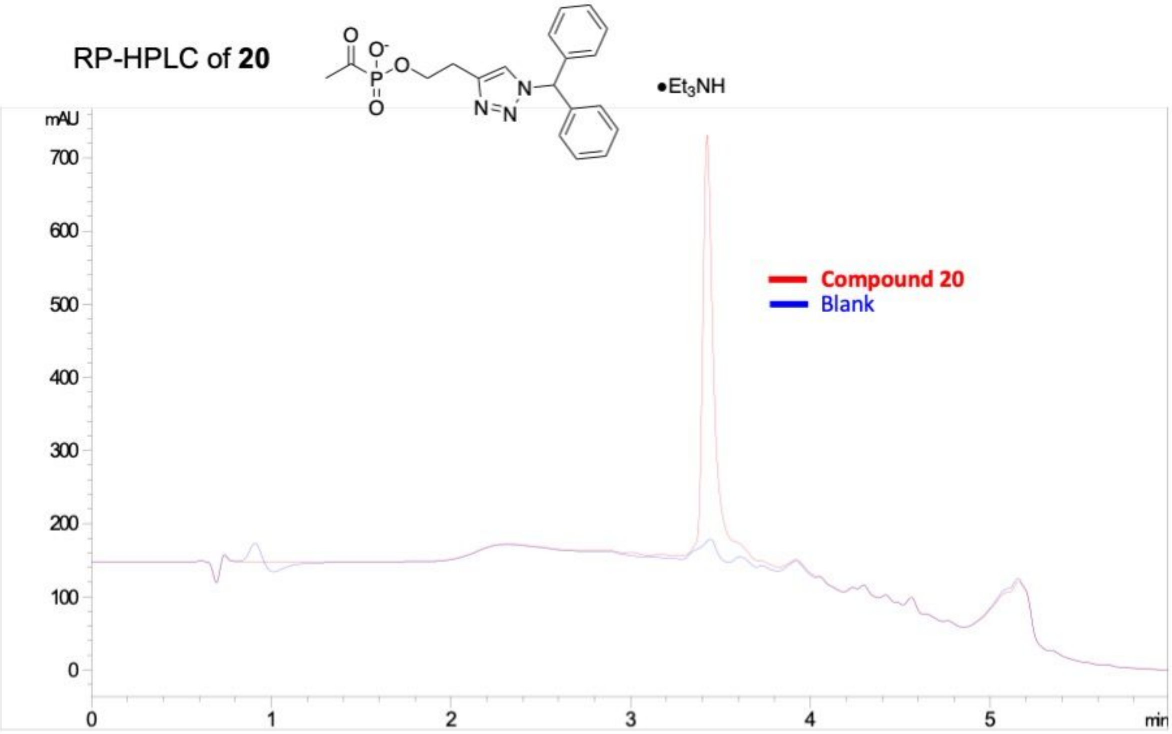

HRMS of **20**

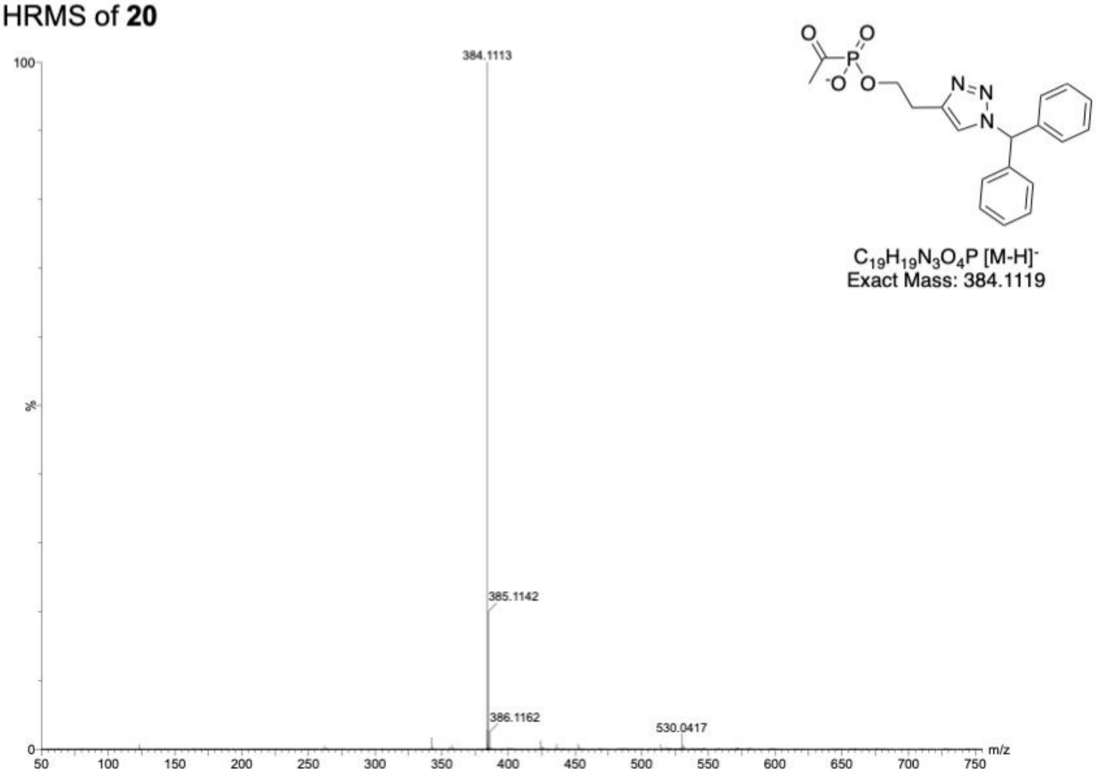

^{1}H NMR of **21**

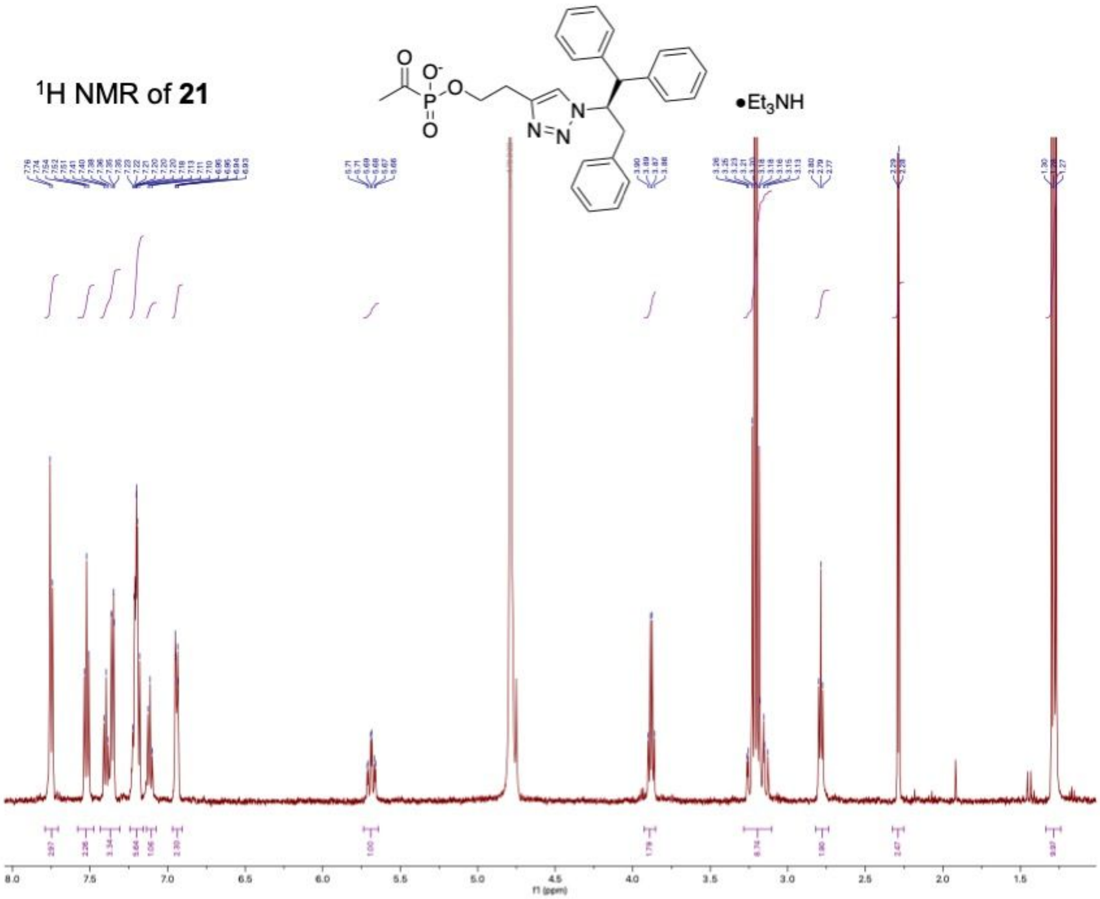

^{31}P NMR of **21**

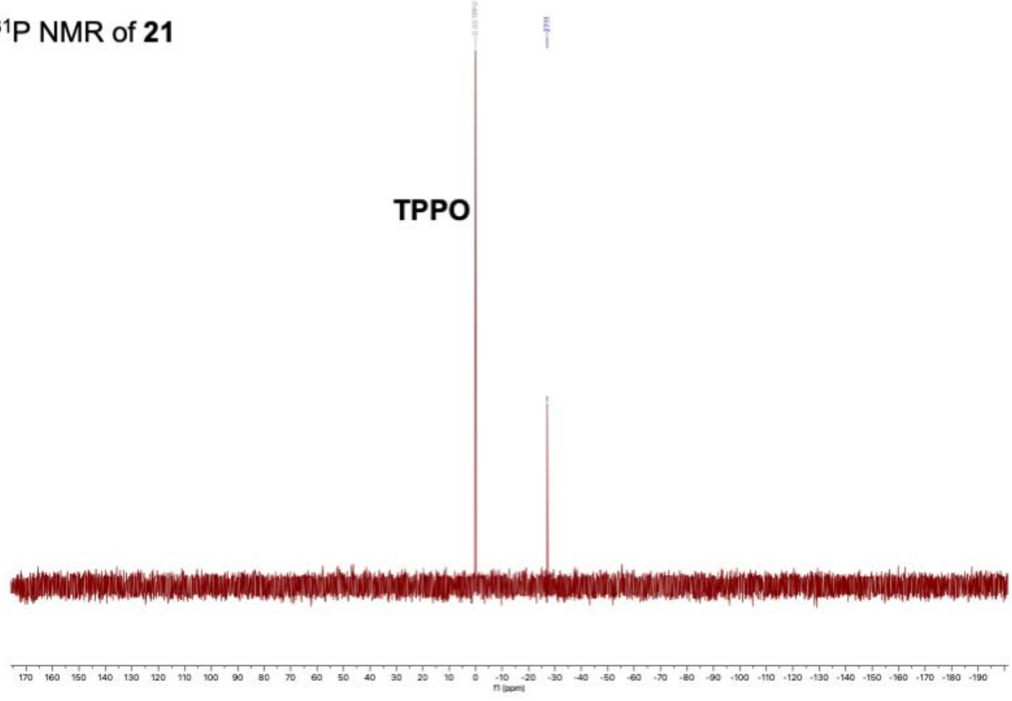

RP-HPLC of **21**

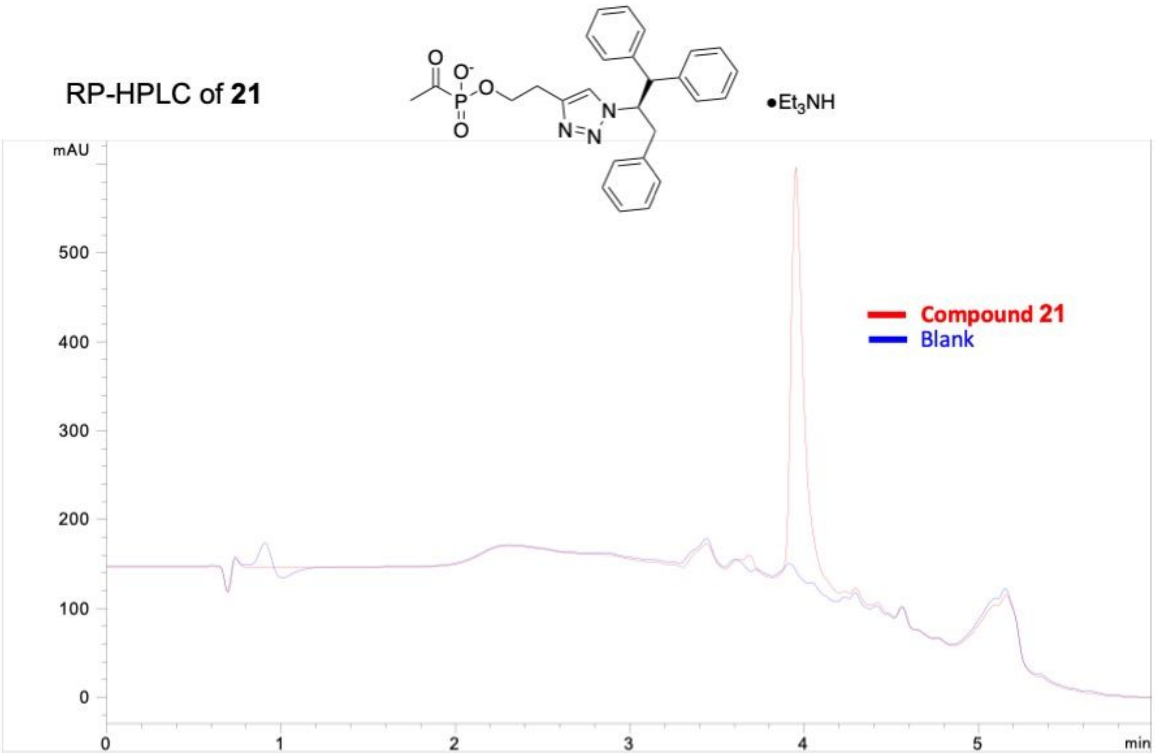

HRMS of **21**

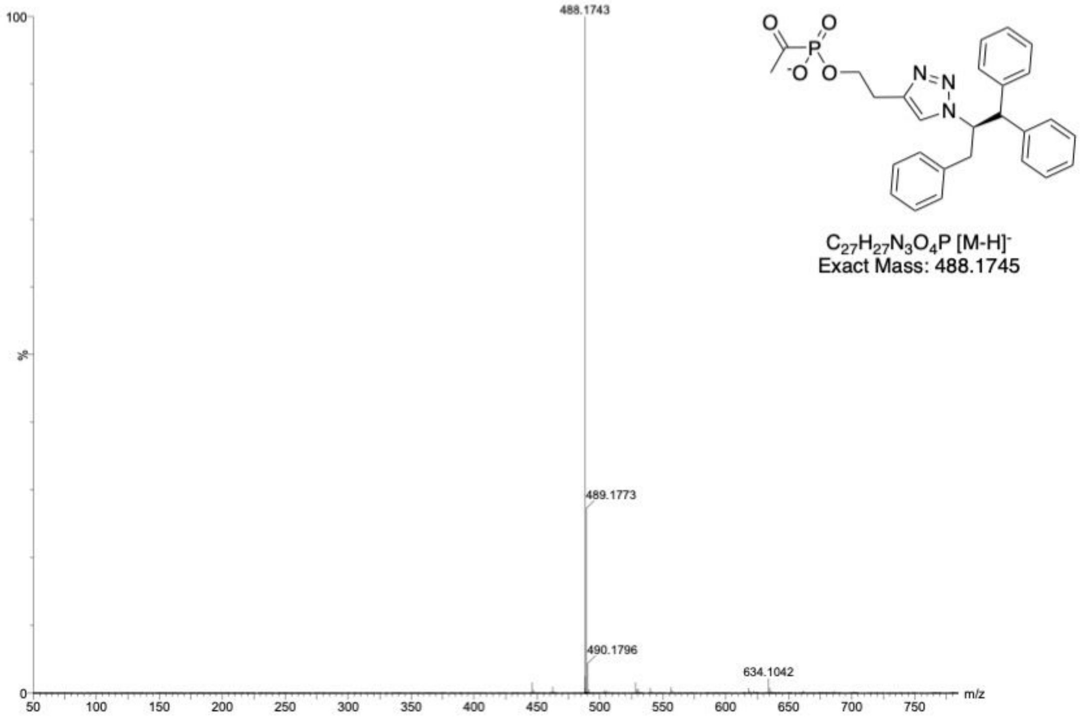

^{1}H NMR of **22**

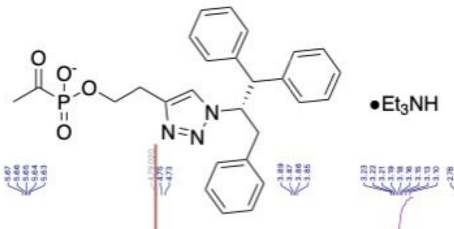

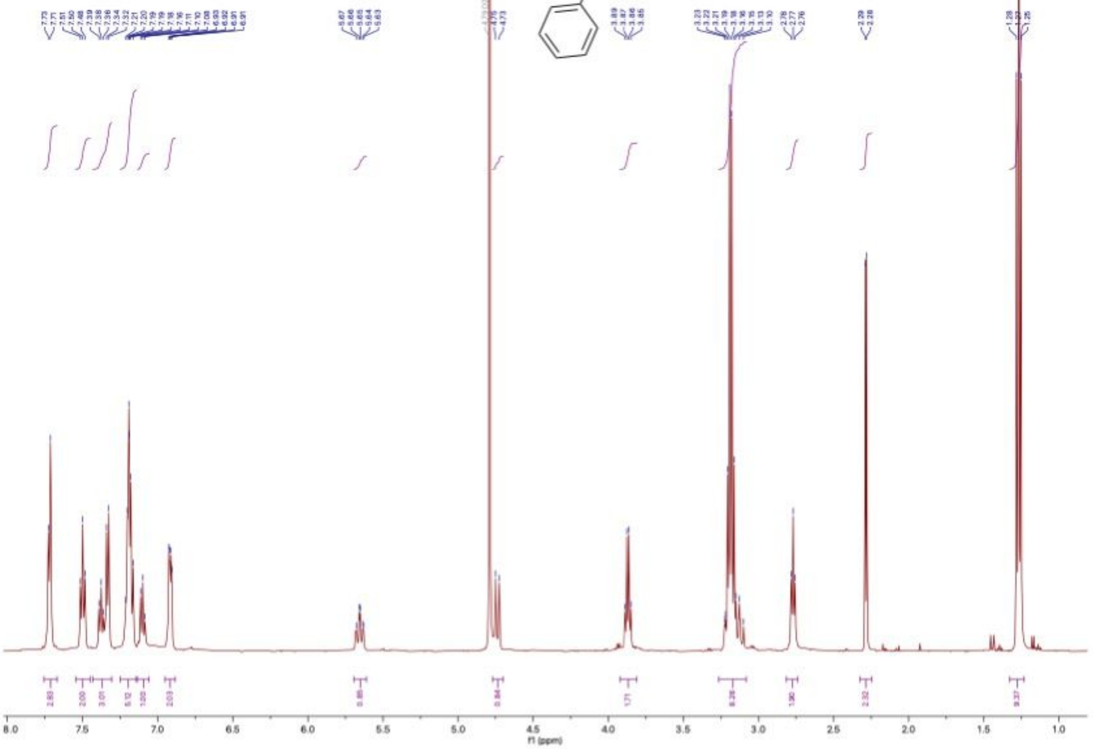

^{31}P NMR of **22**

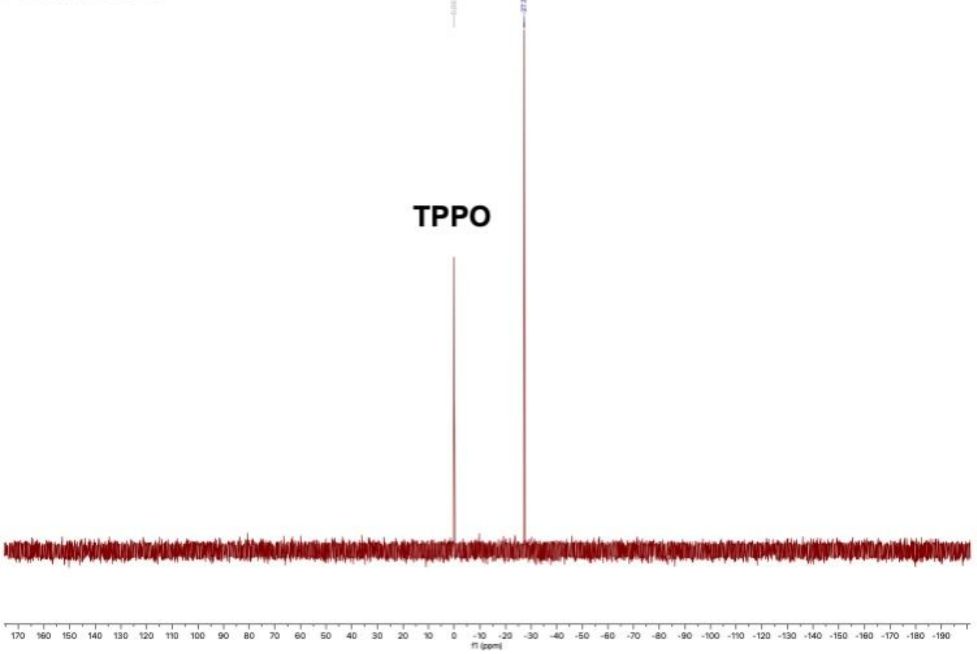

RP-HPLC of **22**

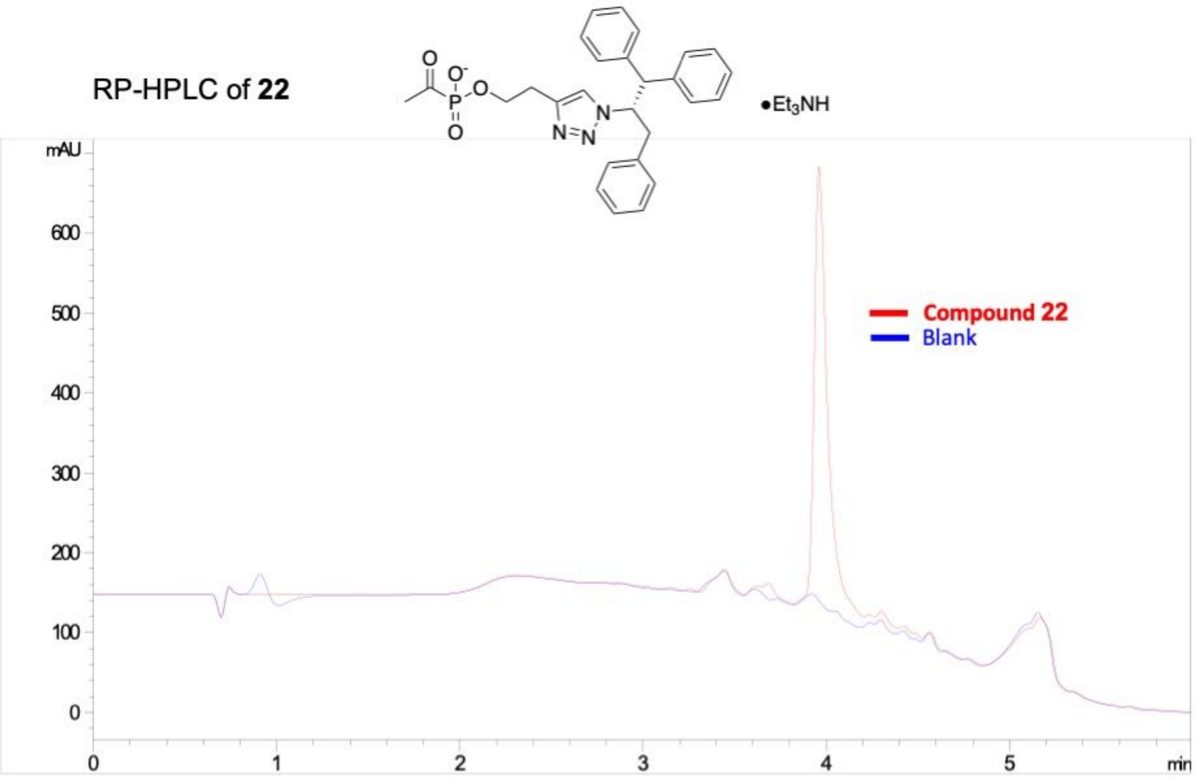

HRMS of **22**

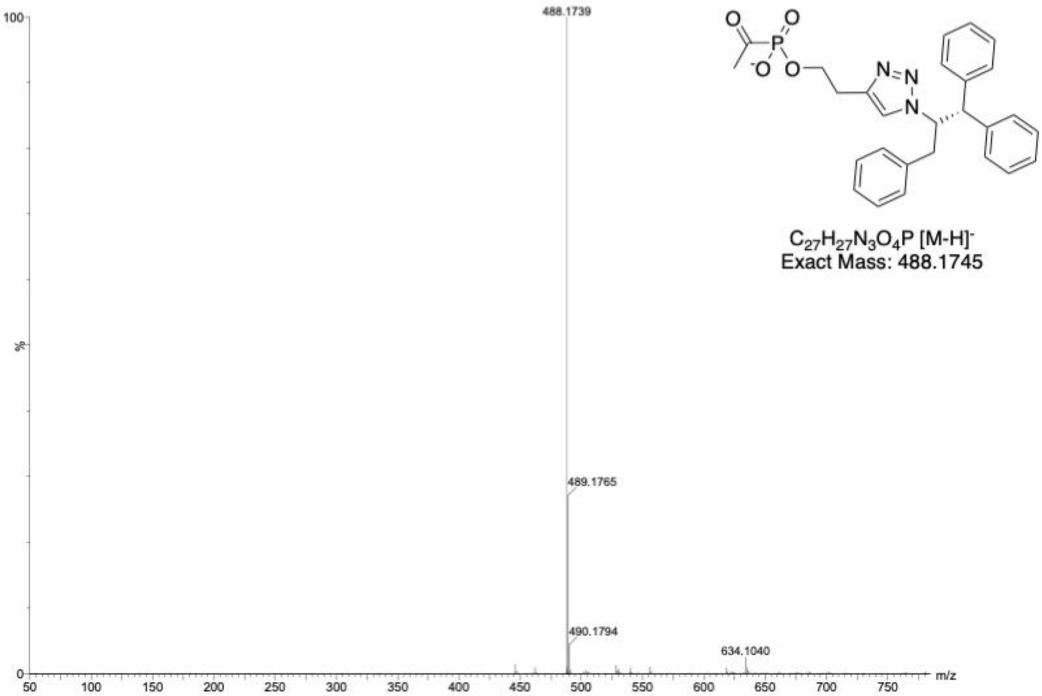

References

(1) Johnston, M. L.; Toci, E. M.; DeColli, A. A.; Freel Meyers, C. L. Antibacterial Target DXP Synthase Catalyzes the Cleavage of D-Xylulose 5-Phosphate: A Study of Ketose Phosphate Binding and Ketol Transfer Reaction. *Biochemistry* **2022**, *61* (17), 1810–1823.

(2) Copeland, R. A. *Evaluation of Enzyme Inhibitors in Drug Discovery: A Guide for Medicinal Chemists and Pharmacologists*, 2nd ed.; John Wiley & Sons: Nashville, TN, 2013.

(3) Potter, G. T.; Jayson, G. C.; Miller, G. J.; Gardiner, J. M. An Updated Synthesis of the Diazo-Transfer Reagent Imidazole-1-Sulfonyl Azide Hydrogen Sulfate. *J. Org. Chem.* **2016**, *81* (8), 3443–3446.

(4) Bartee, D.; Freel Meyers, C. L. Targeting the Unique Mechanism of Bacterial 1-Deoxy-D-Xylulose-5-Phosphate Synthase. *Biochemistry* **2018**, *57* (29), 4349–4356.

(5) Fang, M.; Toogood, R. D.; Macova, A.; Ho, K.; Franzblau, S. G.; McNeil, M. R.; Sanders, D. A. R.; Palmer, D. R. J. Succinylphosphonate Esters Are Competitive Inhibitors of MenD That Show Active-Site Discrimination between Homologous Alpha-Ketoglutarate-Decarboxylating Enzymes. *Biochemistry* **2010**, *49* (12), 2672–2679.
